# Role of phosphatase activity of soluble epoxide hydrolase in regulating simvastatin-activated endothelial nitric oxide synthase

**DOI:** 10.1038/srep13524

**Published:** 2015-08-25

**Authors:** Hsin-Han Hou, Yi-Jen Liao, Sheng-Huang Hsiao, Song-Kun Shyue, Tzong-Shyuan Lee

**Affiliations:** 1 Department of Physiology, School of Medicine, National Yang-Ming University, Taipei, Taiwan; 2 Department of Internal Medicine; National Taiwan University; Taiwan; 3 School of Medical Laboratory Science and Biotechnology, Taipei Medical University, Taipei, Taiwan; 4 Department of Surgery, Zhongxiao Taipei City Hospital, Taipei, Taiwan; 5 Cardiovascular Division, Institute of Biomedical Sciences, Academia Sinica, Taipei, Taiwan; 6 Genome Research Center, National Yang-Ming University, Taipei, Taiwan

## Abstract

Soluble epoxide hydrolase (sEH) has C-terminal epoxide hydrolase and N-terminal lipid phosphatase activity. Its hydrolase activity is associated with endothelial nitric oxide synthase (eNOS) dysfunction. However, little is known about the role of sEH phosphatase in regulating eNOS activity. Simvastatin, a clinical lipid-lowering drug, also has a pleiotropic effect on eNOS activation. However, whether sEH phosphatase is involved in simvastatin-activated eNOS activity remains elusive. We investigated the role of sEH phosphatase activity in simvastatin-mediated activation of eNOS in endothelial cells (ECs). Simvastain increased the phosphatase activity of sEH, which was diminished by pharmacological inhibitors of sEH phosphatase. In addition, pharmacological inhibition of sEH phosphatase or overexpressing the inactive phosphatase domain of sEH enhanced simvastatin-induced NO bioavailability, tube formation and phosphorylation of eNOS, Akt, and AMP-activated protein kinase (AMPK). In contrast, overexpressing the phosphatase domain of sEH limited the simvastatin-increased NO biosynthesis and eNOS phosphorylation at Ser1179. Simvastatin evoked epidermal growth factor receptor–c-Src–increased Tyr phosphorylation of sEH and formation of an sEH–Akt–AMPK–eNOS complex, which was abolished by the c-Src kinase inhibitor PP1 or c-Src dominant-negative mutant K298M. These findings suggest that sEH phosphatase activity negatively regulates simvastatin-activated eNOS by impeding the Akt–AMPK–eNOS signaling cascade.

Simvastatin (Zocor), an inhibitor of 3-hydroxy-3-methylglutaryl coenzyme A reductase, is a cholesterol-lowering drug that suppresses the biosynthesis of cholesterol and enhances the clearance of circulating low-density lipoprotein receptor (LDL) by upregulating the expression of hepatic LDR receptors^1^,^2^. Simvastatin is used to treat hyperlipidemia and related cardiovascular diseases^2^,^3^,^4^. Besides its beneficial effect on dyslipidemia, simvastatin has cholesterol-independent pleiotropic effects on the physiological function of endothelial cells (ECs) by increasing endothelial nitric oxide synthase (eNOS) activity and nitric oxide (NO) production^5^,^6^,^7^,^8^.

Endothelium-derived NO is a cricial regulator of vascular homeostasis; dysregulation of eNOS is the key event in the initiation and progression of cardiovascular diseases such as atherosclerosis and hypertension^9^,^10^. NO bioavailability is tightly controlled by eNOS activity via the complex network of kinase- and phosphatase-dependent pathways^11^,^12^,^13^,^14^,^15^. For instance, treatment with vascular endothelial growth factor (VEGF) increases the phosphoinositide 3-kinase (PI3K)/Akt-dependent phosphorylation of eNOS, which leads to eNOS activation and NO production in ECs^11^,^12^. By contrast, VEGF also activates protein phosphatase 2A (PP2A) to dephosphorylate eNOS and negatively regulate eNOS, thereby precisely modulating the enzymatic activity of eNOS^12^,^14^,^15^. However, despite extensive investigations of the molecular mechanisms of simvastatin, whether phosphatase is functionally involved in simvastatin-activated eNOS in ECs is unknown.

Soluble epoxide hydrolase (sEH) is a bifunctional enzyme with C-terminal hydrolase and N-terminal phosphatase activity^16^,^17^. sEH is expressed in several types of cells, including cardiomyocytes and ECs^17^. sEH hydrolase is responsible for the conversion of epoxyeicosatrienoic acids (EETs) to dihydroxyeicosatrienoic acids (DHETs). EETs are synthesized from arachidonic acid by cytochrome P450 epoxygenases and modulate cellular physiologic function including vascular relaxation, inflammatory response and fibrosis in the cardiovascular system^17^,^18^,^19^,^20^. Several lines of evidence suggest that inhibition of sEH hydrolase activity increases circulating levels of EETs and prevents the progression of hypertension and inflammatory diseases^20^,^21^,^22^,^23^,^24^.

In contrast to knowledge of the hyrolase activity of sEH, that of its phosphatase activity is limited. Human sEH polymorphism studies demonstrated that Arg287Gln or Lys55Arg polymorphism of sEH, encoding a mutant sEH with reduced phosphatase activity, is associated with coronary heart diseases or type 2 diabetes^25^,^26^,^27^,^28^. In addition, sEH phosphatase is involved in the cholesterol metabolism of hepatocytes, cell growth of ECs and metabolism of lysophosphatidic acids^29^,^30^,^31^,^32^. Nevertheless, whether the phosphatase activity of sEH participates in simvastatin-activated eNOS and NO production and the underlying regulatory mechanism are largely unknown.

In this study, we hypothesized that sEH phosphatase plays an important role in simvastatin-regulated eNOS activity. We investigated the change in sEH phosphatase activity with simvastatin treatment and then examined the effect of the phosphatase domain of sEH on simvastatin-induced eNOS phosphorylation and NO production as well as the Akt-AMP-activated protein kinase (AMPK) signaling pathway. We also delineated the mechanisms underlying sEH phosphatase-regulated eNOS activity in simvastatin-treated ECs. We found that sEH phosphatase is a crucial negative regulator of simvastatin-activated eNOS by activating epidermal growth factor receptor (EGFR)–c-Src signaling.

## Results

### Simvastatin increases sEH phosphatase activity

**Figure 1:**
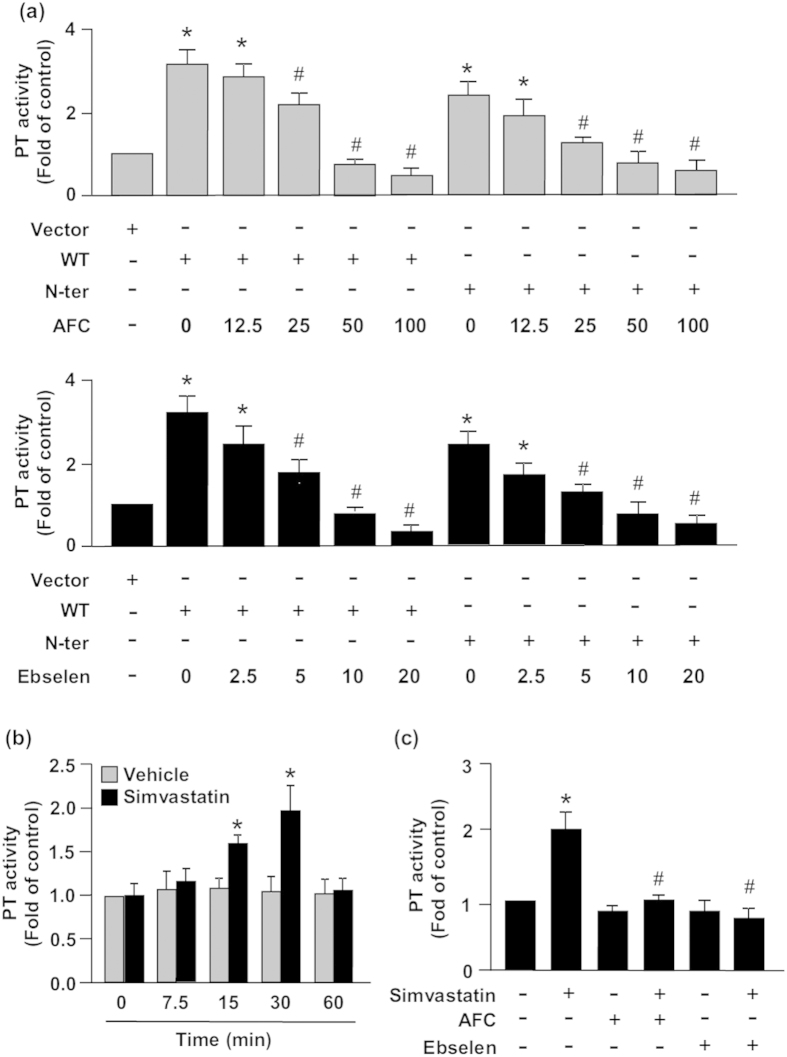
Simvastatin increases soluble epoxide hydrolase (sEH) phosphatase activity. Phosphatase activity (PT) assay of bovine aortic endothelial cells (BAECs) (**a**) transfected with vector, full-length sEH (WT) or phosphatase domain of sEH (N-ter) for 48 h, then treated with or without an sEH phosphatase inhibitor, N-acetyl-S-farnesyl-L-cysteine (AFC, 0, 12.5, 25, 50, 100 μM) or ebselen (0, 2.5, 5, 10, 20 μM), for 2 h; (**b**) treated with vehicle or simvastatin (10 μM) for the indicated times; or (**c**) pretreated with AFC or ebselen (10 μM) for 2 h, then simvastatin (10 μM) for 30 min. Data are mean ± SEM from 5 independent experiments. ^*^P < 0.05 vs. vehicle- or vector-treated cells, ^#^*P* < 0.05 vs. simvastatin-treated alone cells.

We first validated the inhibitory effect of the sEH phosphatase inhibitors AFC or ebselen on sEH phosphatase activity in BAECs cells transfected with full-length sEH (WT) or the phosphatase domain of sEH (N-ter). These transfected BAECs were then treated with various concentrations of AFC (0, 12.5, 25, 50, 100 μM) or ebselen (0, 2.5, 5, 10, 20 μM) for 2 h. Our data showed that treatment with AFC or ebselen could dose-dependent inhibit the activity of sEH phosphatase activity (Fig. 1a), suggesting the two inhibitors have excellent inhibitory efficacy on phosphatase activity. However, treatment with 100 μM of AFC or 20 μM of ebselen showed cytotoxic effects to BAECs (data not shown). We therefore chose AFC at concentration 50 μM or ebselen at concentration 10 μM, respectively, for our further studies. We then evaluated the role of sEH phosphatase in regulating simvastatin-mediated effects in ECs. Time-course experiments demonstrated a significant increase in sEH phosphatase activity with 10 μM simvastatin as early as 15 min, with peak level at 30 min (Fig. 1b). Pre-treatment with AFC or ebselen totally abrogated the simvastatin-increased sEH phosphatase activity (Fig. 1c).

### sEH phosphatase negatively regulates simvastatin-induced NO production, tube formation and eNOS phosphorylation in ECs

**Figure 2:**
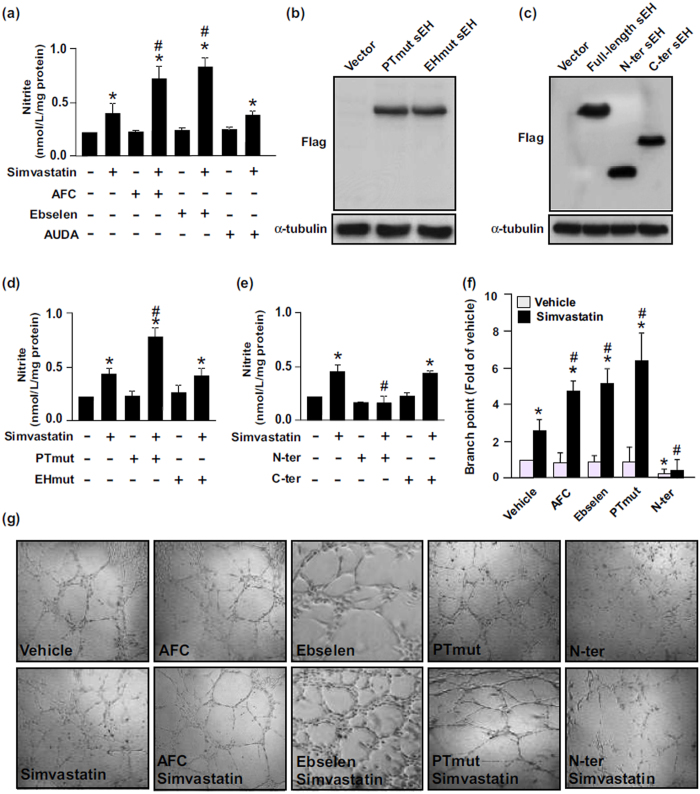
Phosphatase activity of sEH negatively regulates simvastatin-induced NO production and tube formation in ECs. (**a**) BAECs were pretreated with the sEH phosphatase inhibitor AFC (50 μM) or ebselen (10 μM) for 2 h or sEH hydrolase inhibitor 12-(3-adamantan-1-yl-ureido)-dodecanoic acid (AUDA, 10 μM), for 2 h and then simvastatin (10 μM) for 24 h. Level of nitrite in culture media was measured by Griess assay. (**b**–**e**) BAECs transfected with vector, Flag-tag sEH with PTmut or hydrolase mutant (EHmut); or Flag-tag N-ter or hydrolase domain of sEH (C-ter) for 48 h, then simvastatin (10 μM) for 24 h. (**b**,**c**)Western blot analysis of Flag and α-tubulin. (**d**,**e**) Level of nitrite in culture media. (**f**,**g**) Tube formation with BAECs pretreated with AFC (50 μM) or ebselen (10 μM) for 2 h or transfected with PTmut or N-ter for 48 h, then seeded on pre-coated ECL Cell Attachment Matrix with or without simvastatin (10 μM) for 24 h. Data are mean ± SEM fold branch points from 5 randomly selected microscopy views. ^*^P < 0.05 vs. vehicle- or vector-treated cells, ^#^*P* < 0.05 vs. simvastatin-treated alone cells.

**Figure 3:**
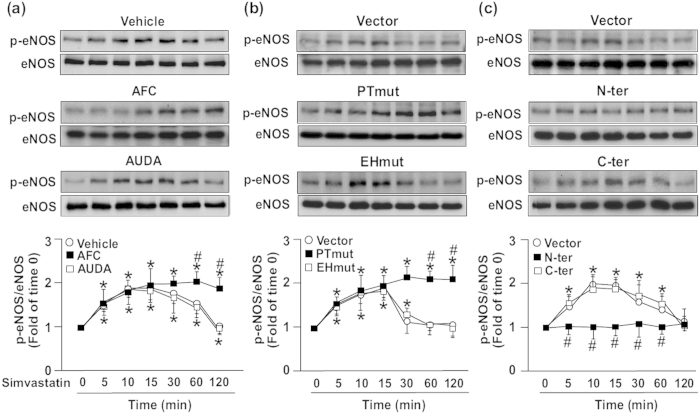
Role of sEH phosphatase in regulating simvastatin-induced eNOS phosphorylation. Western blot analysis of phosphorylated eNOS (p-eNOS) at Ser1179 and eNOS level in BAECs pretreated with (**a**) vehicle, AFC (50 μM) or AUDA (10 μM) for 2 h or (**b**,**c**) transfected with vector, Ptmut, Ehmut, N-ter or C-ter for 48 h, then simvastatin (10 μM) for 0–120 min. Data are mean ± SEM from 5 independent experiments. ^*^P < 0.05 vs. vehicle- or vector-treated cells, ^#^*P* < 0.05 vs. simvastatin-treated alone cells.

Simvastatin can induce eNOS phosphorylation and NO production in ECs^6^,^33^. Therefore, we explored the effect of sEH phosphatase on simvastatin-mediated NO production and tube formation as well as phosphorylation of eNOS, Akt and AMPK in bovine aortic endothelial cells (BAECs). To evaluate the specific role of sEH phosphatase in these key simvastatin-induced events, BAECs were pre-treated with AFC, ebselen, or AUDA (an inhibitor of sEH hydrolase) or transfected with PTmut, inactive EH domain (EHmut) (EHmut), N-ter or C-terminal hydrolase domain (C-ter). Treatment with AFC or ebselen or transfection with PTmut further increased the simvastatin-increased NO bioavailability and tube formation (Fig. 2a,d). However, treatment with AUDA or transfection with EHmut failed to produce such effects (Fig. 2a,b). In addition, overexpression of N-ter prevented the simvastatin-induced NO production and tube formation (Fig. 2e–g). eNOS phosphorylation status is critical for its enzymatic activity in BAECs^10^,^11^. We found that treatment with AFC but not AUDA prolonged the simvastatin-mediated eNOS phosphorylation at Ser1179 (Fig. 3a).

To provide further evidence that sEH phosphatase is crucial in eNOS activation, we depleted the activity of sEH phosphatase by overexpressing a full-length sEH with catalytic-inactive phosphatase. Simvastatin-mediated eNOS phosphorylation at Ser1179 was further augmented with overexpression of the inactive phosphatase domain (PTmut), whereas overexpression of the inactive EH domain (EHmut) had no effect (Fig. 3b). In contrast, overexpression of the phosphatase domain of sEH (N-ter) totally abrogated the simvastatin-increased eNOS phosphorylation at Ser1179 (Fig. 3c). sEH phosphatase may be important in regulating simvastatin-activated eNOS.

### Overexpression of sEH phosphatase diminishes simvastatin-induced phosphorylation of AMPK and Akt

**Figure 4:**
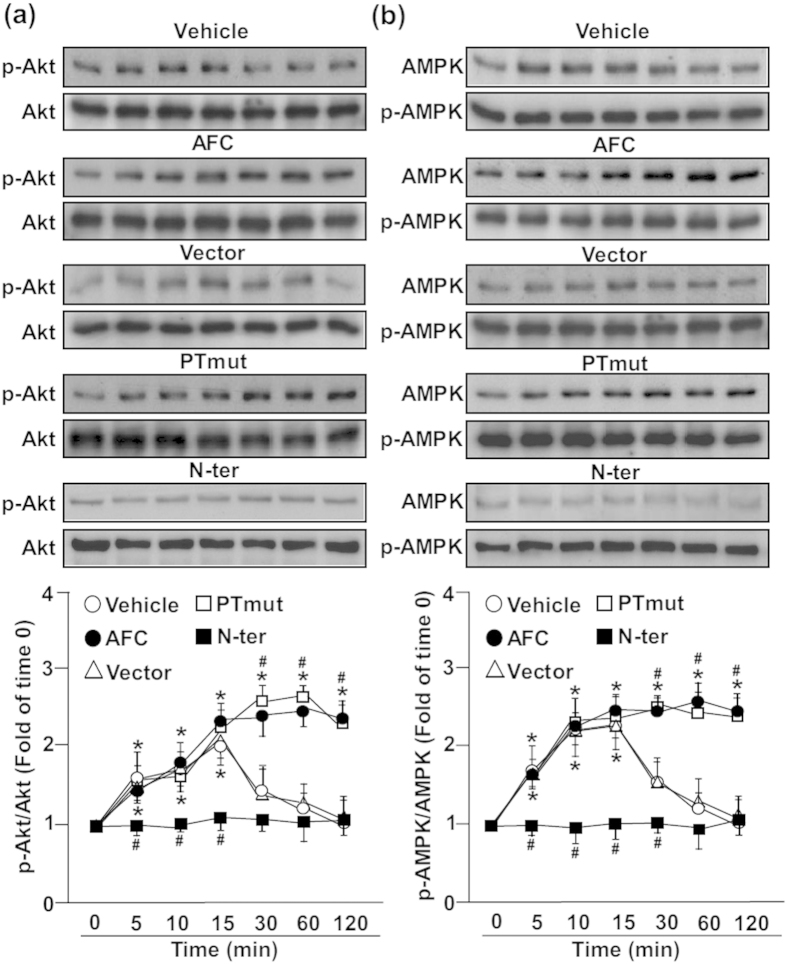
sEH phosphatase plays a crucial role in regulating simvastatin-induced Akt and AMPK phosphorylation. Western blot analysis of (**a**) phosphorylated Akt (p-Akt) at Ser473 and Akt and (**b**) phosphorylated AMP-activated protein kinase (p-AMPK) at Thr172 and AMPK in BAECs pretreated with vehicle or AFC (50 μM) or transfected with vector, PTmut or N-ter for 48 h, then simvastatin (10 μM) for 0–120 min. Data are mean ± SEM from 5 independent experiments. ^*^P < 0.05 vs. vehicle- or vector-treated cells, ^#^*P* < 0.05 vs. simvastatin-treated alone cells.

The Akt–AMPK signaling pathway plays a crucial role in simvastatin-activated eNOS^33^. We analyzed whether sEH phosphatase is involved in activation of the Akt–AMPK pathway. Treatment with AFC or transfection with PTmut sustained simvastatin-induced Akt phosphorylation at Ser473 and AMPK phosphorylation at Thr172 (Fig. 4a,b). Moreover, overexpression of the phosphatase domain of sEH (N-ter) totally abolished these simvastatin-increased activities (Fig. 4a,b). Therefore, sEH phosphatase participates in simvastatin-activated Akt–AMPK signaling in ECs.

### Simvastatin increases the formation of an sEH–eNOS–Akt–AMPK complex

**Figure 5:**
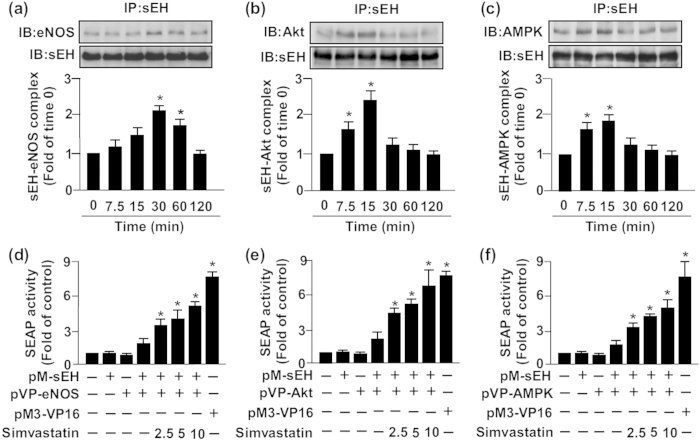
Simvastatin promotes the formation of an sEH–eNOS–Akt–AMPK complex. BAECs were treated with simvastatin (10 μM) for 0–120 min. Cellular lysates was immunoprecipitated (IP) with anti-sEH Ab and then immunoblotted (IB) with (**a**) anti-eNOS, (**b**) anti-Akt, (**c**) anti-AMPK or anti-sEH Abs. Secreted human alkaline phosphatase (SEAP) activity in culture media from BAECs co-transfected with pM-sEH and (**d**) pVP-eNOS, (**e**) pVP-Akt or (**f**) pVP-AMPK for 48 h, then indicated concentrations of simvaststin (2.5, 5, 10 μM) for another 24 h. pM3-VP16–transfected cells were a positive control. Data are mean ± SEM from 5 independent experiments. ^*^P < 0.05 vs. vehicle-treated group.

The physical interaction of eNOS with intracellular proteins such as Akt and AMPK is a key molecular mechanism in regulating eNOS activity^14^,^34^. However, whether sEH could interact directly with eNOS, Akt or AMPK remains elusive. We next investigated whether sEH phosphatase is involved in the simvastatin-induced association of eNOS, Akt and AMPK. IP assay revealed that simvastatin time-dependently increased the interaction between sEH and eNOS, Akt and AMPK (Fig. 5a–c). In addition, mammalian two-hybrid assay findings supported this notion that sEH can bind directly with eNOS, Akt and AMPK (Fig. 5d–f). sEH phosphatase might participate in simvastatin-activated Akt–AMPK–eNOS signaling by protein–protein interaction in ECs.

### EGFR–c-Src signaling is required for simvastatin-increased sEH Tyr phosphorylation and formation of an eNOS–Akt–AMPK complex

**Figure 6:**
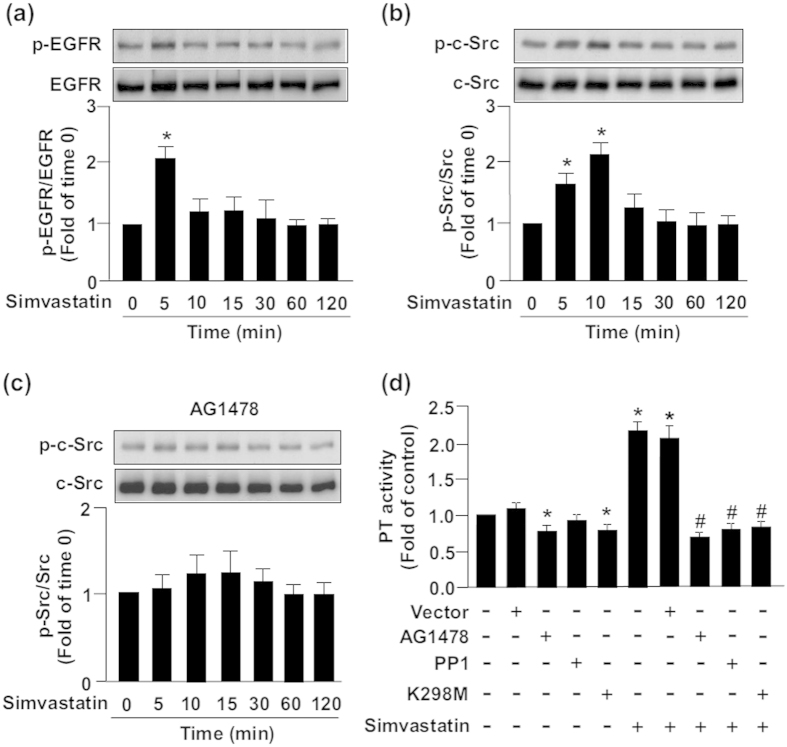
Activation of epidermal growth factor receptor (EGFR)–c-Src signaling is required for simvastatin-induced increase in sEH phosphatase activity. BAECs were treated with simvastatin (10 μM) for 0–120 min. Cellular lysates were IB with (**a**) anti-phospho-epidermal growth factor receptor (p-EGFR) and EGFR and (**b**) anti-phospho-c-Src and c-Src. (**c**) Western blot analysis of anti-phosphor-c-Src and c-Src levels in BAECs pretreated with AG1478 (1 μM, a EGFR inhibitor) for 2 h, then simvastatin (10 μM) for 0–120 min. (**d**) Phosphatase activity (PT) assay of cells pretreated with AG1478 (1 μM) or protein phosphatase 1 (PP1; 10 μM) or transfected with K298M (a c-Src dominant-negative mutant) for 48 h, then simvastatin for 30 min. Data are mean ± SEM from 5 independent experiments. ^*^P < 0.05 vs. vehicle- or vector-treated cells, ^#^*P* < 0.05 vs. simvastatin-treated alone cells.

**Figure 7:**
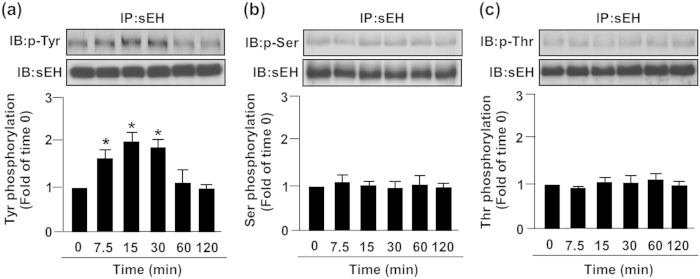
Simvastatin induces tyrosine phosphorylation of sEH. BAECs were treated with simvastatin (10 μM) for 0–120 min. Cellular lysates were IP with anti-sEH Ab, then IB with Ab for anti-sEH and (**a**) anti-Tyr phosphorylation, (**b**) anti-Ser phosphorylation or (**c**) anti-Thr phosphorylation. Data are mean ± SEM from 5 independent experiments. ^*^P < 0.05 vs. vehicle-treated group.

EGFR–c-Src signaling was predicted to be a pathway mediating sEH Tyr phosphorylation by use of the web tools NetPhos 2.0 and NetPhosK 1.0 (http://www.cbs.dtu.dk/services/NetPhos/)^35^. In addition, c-Src kinase plays a key role in eNOS activation^36^. Therefore, we examined whether the simvastatin-induced formation of sEH–Akt–AMPK–eNOS complex requires Tyr phosphorylation of sEH by EGFR–c-Src signaling. Treatment with simvastatin time-dependently increased the phosphorylation of EGFR and c-Src (Fig. 6a,b). Pretreatment with AG1478, an inhibitor of EGFR signaling, abolished the simvastatin-increased phosphorylation of c-Src (Fig. 6c). Mammalian two-hybrid assay further revealed that simvastatin increased the interaction of EGFR and c-Src, which was prevented by treatment with AG1478, PP1 (a c-Src inhibitor) or K298M (a c-Src dominant-negative mutant) (Fig. 6d). Furthermore, simvastatin induced transient Tyr phosphorylation of sEH but not Ser or Thr phosphorylation of sEH within 7.5 to 30 min after treatment, which returned to the basal level at 60 min after treatment (Fig. 7a–c).

**Figure 8:**
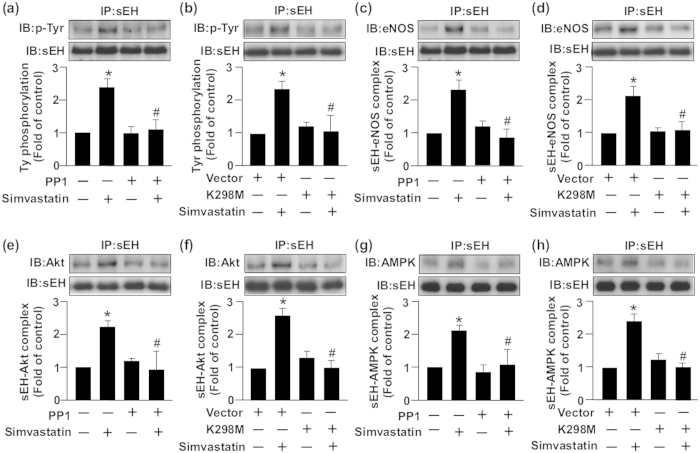
c-Src signaling is essential for simvastatin-induced sEH tyrosine phosphorylation and the formation of an sEH–Akt–AMPK–eNOS complex. BAECs were pretreated with or without PP1 (10 μM) for 2 h (**a**,**c**,**e**,**g**) or transfected with K298M for 48 h (**b**,**d**,**f**,**h**), then incubated with simvastatin (10 μM) for 15 min (**a**,**b**,**e**–**g**) or 30 min (**c**,**d**). Cellular lysates were IP with anti-sEH Ab and then IB with Ab for anti-sEH, (**a**,**b**) anti-Tyr phosphorylation, (**c**,**d**) eNOS, (**e**,**f**) Akt or (**g**,**h**) AMPK. Data are mean ± SEM from 5 independent experiments. ^*^P < 0.05 vs. vehicle- or vector-treated group, ^#^*P* < 0.05 vs. simvastatin-treated alone group.

**Figure 9:**
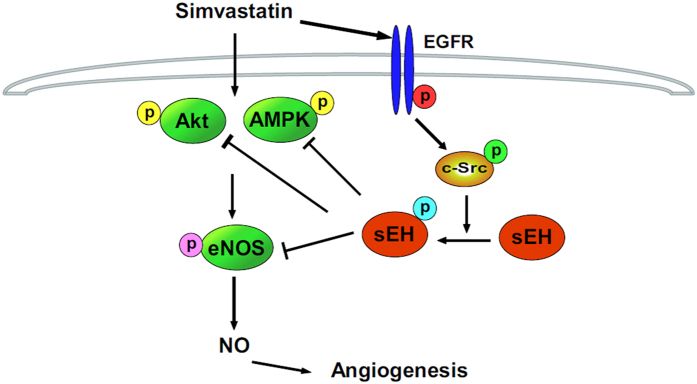
Illustration of proposed mechanism of sEH phosphatase regulating simvastatin-induced eNOS activation and NO production in ECs. Simvastatin may activate EGFR–c-Src signaling to increase sEH Tyr phosphorylation, thus activating sEH phosphatase. This key event may negatively regulate simvastatin-activated Akt-AMPK-eNOS signaling and NO production in ECs.

We further evaluated whether c-Src is involved in simvastatin-altered Tyr phosphorylation of sEH and the formation of an sEH–Akt–AMPK–eNOS complex. Simvastatin-induced Tyr phosphorylation of sEH was abrogated by PP1 or K298M treatment (Fig. 8a,b). In addition, blocking the activation of c-Src signaling with PP1 or K298M abolished the simvastatin-induced association of sEH with eNOS (Fig. 8c,d), Akt (Fig. 8e,f) and AMPK (Fig. 8g,h). Thus, EGFR–c-Src signaling is essential for the negative regulatory mechanism of simvastatin on eNOS activation (Fig. 9).

## Discussion

In this study, we characterized a new role for sEH phosphatase in simvastatin-mediated eNOS activation and NO production. Exposing ECs to simvastatin increased sEH phosphatase activity, which, in turn, negatively modulated the simvastatin-induced phosphorylation of Akt, AMPK and eNOS, thus leading to maintained NO production. Treatment with pharmacological inhibitors or genetic manipulation further revealed that the simvastatin-increased sEH phosphatase activity depended on activation of EGFR–c-Src signaling. Along with eNOS activation, exposing ECs to simvastatin rapidly induced Tyr phosphorylation of sEH and profoundly increased the physical interaction of sEH with Akt, AMPK and eNOS. Notably, the increased Tyr phosphorylation of sEH and sEH–Akt–AMPK–eNOS interaction was inhibited by the c-Src kinase antagonist PP1 or c-Src kinase dominant-negative mutant K298M. These results agreed with our previous findings that c-Src–sEH phosphatase signaling negatively regulates VEGF-activated eNOS^37^. Thus, the negative regulation of eNOS activity by sEH phosphatase may not be limited to simvastatin stimulation alone. The phosphatase activity of sEH may be pivotal in regulating eNOS activity and the physiological function of ECs.

Besides sEH phosphatase, protein serine/threonine phosphatase 1 (PP1), PP2A and PP2B are also involved in regulation of eNOS activity^14^. Therefore, we assessed whether PP1, PP2A or PP2B contributes to simvastatin-induced eNOS phosphorylation. Simvastatin did not affect the activity of PP1, PP2A or PP2B (data not shown). In addition, pretreatment with a PP1-, PP2A- or PP2B-specific inhibitor did not alter the status of eNOS phosphorylation elicited by simvastatin (data not shown). Thus, sEH phosphatase indeed has a unique role in regulating simvastatin-induced eNOS activation. The phosphatase activity was lower for sEH N-ter than full-length sEH in cell lysates. This result agrees with Nelson *et al.*, who found lower phosphatase activity with purified N-ter than WT sEH *in vitro*^38^. Under physiologic conditions, sEH is a homodimer with two domains, the C-terminal epoxide hydrolase and N-terminal phosphatase activity domain^16^. The entire homodimer structure of sEH is important for maintaining sEH enzymatic activity^32^. Therefore, loss of the hydrolase domain of sEH may be attributed to decreased phosphatase activity of sEH N-ter.

sEH phosphatase is thought to have isoprenoid mono- and pyrophosphate activity and prefer hydrolysis phosphates of lipophilic compounds in cells^39^,^40^. Indeed, lysophosphatidic acids are biological substrates for sEH phosphatase^31^,^32^. Therefore, sEH phosphatase presumably is a key regulator for lipid signaling-related cellular function. However, the biological significance of sEH phosphatase activity and the detailed signaling pathways underlying its regulation of eNOS activity remain unclear. In our previous and current studies, we targeted sEH phosphatase as a regulator of eNOS activation. Inhibiting the phosphatase activity of sEH augmented VEGF-37 or simvastatin-induced eNOS phosphorylation and NO production, whereas overexpression of sEH phosphatase abolished these effects. sEH may be a crucial negative modulator of eNOS phosphorylation and NO production.

The biological role of sEH hydrolase activity and its substrate EETs in the functioning of the cardiovascular system and the development of cardiovascular diseases is well established^17^,^18^,^19^,^20^. Inhibition of sEH hydrolase activity by pharmacological antagonists or genetic deletion of sEH increases EET levels in blood, thereby alleviating the progression of angiotensin II-mediated hypertension, atherosclerosis and cardiac hypertrophy in experimental animal models^24^,^41^,^42^. Therefore, inhibition of sEH hydrolase by pharmacological antagonism may be a therapeutic strategy for treating cardiovascular diseases. However, sEH-knockout mice lack both phosphatase and hydrolase domains, so determining whether sEH phosphatase contributes to the beneficial effects with deletion of sEH *in vivo* is difficult. Indeed, several lines of evidence suggest that genetic ablation of sEH exacerbates the progression of many diseases, albeit with an unclear mechanism^43^,^44^. Further investigation delineating the exact role and molecular mechanisms of sEH phosphatase in the regulation of cardiovascular function and pathogenesis of cardiovascular diseases is warranted.

In addition to the kinase- and phosphatase-dependent regulation of eNOS activity, physical interaction of eNOS with intracellular proteins is a crucial mechanism in regulating eNOS activation with stimuli^14^,^34^. In this study, simvastatin increased the association of sEH and Akt, AMPK and eNOS in ECs and increased the Tyr phosphorylation of sEH. More importantly, c-Src kinase, a key regulator in eNOS activation by simvastatin or other agonists in ECs^36^,^37^, was the upstream signaling molecule for Tyr phosphorylation of sEH and the formation of the sEH–Akt–AMPK–eNOS complex. Our findings indicate that both kinase-dependent regulation and protein–protein interaction are involved in the simvastatin-mediated Tyr phosphorylation of sEH and interaction with kinases and eNOS as well as NO bioavailability. However, the exact site(s) of Tyr phosphorylation of sEH and the underlying molecular mechanisms in the cross-talk among c-Src, sEH Akt, AMPK and eNOS remain to be uncovered.

ECs play a crucial role in regulating vascular homeostasis, including blood cells and nutritional trafficking to tissues, coagulation and vascular tone^9^,^10^. Endothelial dysfunction is known to be a key event in the development of atherosclerosis. Statins are known to improve endothelial dysfunction by increasing eNOS activation and thus reduce cardiovascular morbidity and mortality in patients with coronary heart disease^5^,^6^,^7^,^8^. In this study, our findings suggest sEH phosphatase serves as a negative regulator in the simvastatin-induced eNOS activation. Inhibition of sEH phosphatase activity can sustain the simvastatin-induced phosphorylation of Akt, AMPK and eNOS, all of which lead to further augmented NO production. On the other hand, EETs have been reported to have protective effects on endothelial dysfunction and cardiovascular diseases^18^,^19^. Presumably, these findings imply that statin-sEH phosphatase inhibitor or sEH hydrolase-phosphatase inhibitor combination therapy might be of high therapeutic values in treating eNOS-related cardiovascular diseases.

In summary, we provide new evidence that simvastatin activates EGFR–c-Src signaling, which increases Tyr phosphorylation of sEH and sEH–Akt–AMPK–eNOS interaction. These events may work in concert to modulate simvastatin-induced eNOS activation and NO production in ECs. The molecular mechanisms we reveal may provide new information for better understanding the regulation of eNOS activation and for suggesting novel pharmacological targets for treating eNOS-related cardiovascular diseases.

## Methods

### Reagents

Rabbit antibody (Ab) for phosphor-eNOS at Ser1177, phosphor-Akt at Ser473, phosphor-AMPK at Thr172, phospho-epidermal growth factor receptor (EGFR) at Tyr845, phosphor-c-Src at Tyr416 and mouse Ab for phosphor-Ser and phosphor-Thr were from Cell Signaling Technology (Beverly, MA, USA). Rabbit Abs for eNOS, sEH, Akt, and AMPK; mouse Ab for EGFR; and protein A/G PLUS-Agarose were from Santa Cruz Biotechnology (Santa Cruz, CA, USA). Mouse Ab for phosphor-Tyr was from BD Biosciences (San Jose, CA, USA) and for α-tubulin, simvastatin, PP1, 4-nitrophenyl phosphate, ebselen and Griess reagent were from Sigma-Aldrich (St Louis, MO, USA). ECL Cell Attachment Matrix was from Millipore (Bedford, MA, USA). N-acetyl-S-farnesyl-L-cysteine (AFC) was from Enzo Life Sciences (Farmingdale, NY, USA). 12-(3-Adamantan-1-yl-ureido)-dodecanoic acid (AUDA) and AG1478 were from Cayman (Ann Arbor, MI, USA).

### Cell culture

Bovine aortic endothelial cells (BAECs) were purchased from Cell Applications (San Diego, CA, USA) and cultured in Dulbecco’s modified Eagle’s medium with 10% fetal bovine serum, 100 unit/ml penicillin and 100 mg/ml streptomycin (HyClone, Logan, UT, USA) in a humidified 95% air and 5% CO_2_ incubator at 37 °C.

### Phosphatase activity assay

Treated BAECs were collected in phosphate buffered saline (PBS), which was sonicated, and supernatant was collected by brief centrifugation. The cell lysate supernatant was added to 4-nitrophenyl phosphate to 2 mM and incubated in 37 °C for 1 h. The yellow color product was detected by OD 405 nm to determine phosphatase activity.

### Detection of nitrite production

Griess reagent was used to evaluate accumulated nitrite, the stable breakdown product of NO, in culture media. After incubation of an equal volume of Griess reagent with medium from treated cells at room temperature for 15 min, the solution was evaluated by SP-8001 UV/VIS spectrophotometry (Taipei) to detect azo dye production at OD 540 nm. Sodium nitrite was used as a standard.

### Plasmid construction

Coding regions for the *sEH* N-terminal phosphatase domain (N-ter) and C-terminal hydrolase domain (C-ter) were amplified from mouse cDNA by PCR with the primers 5′-TTA CGC GTG CGC TGC GTG TAG CCG-3′ and 5′-GGT CTA GAC TAC CCT GTG ACC TTC TCC A-3′ for N-ter and 5′-TTA CGC GTG TCA GCC ATG GAT ATG TGA C-3′ and 5′-GGT CTA GAC TAA ATC TTG GAG GTC ACT G-3′ for the C-ter. The protocol was 2 min at 94 °C, then 15 sec at 94 °C, 30 sec at 58 °C and 2 min at 72 °C for 35 cycles. PCR products were digested with *Mlu*I and *Xba*I for cloning into pCMV5N-Flag vector. The *sEH* full-length cDNA and sEH with mutant hydrolase or phosphatase domain {WT, Ehmut, or Ptmut, respectively; kindly provided by Dr. S. Imaoka (Kwansei Gakuin University, Japan)^31^ were amplified by PCR (2 min at 94 °C, then 15 sec at 94 °C, 30 sec at 61 °C and 2 min at 72 °C for 35 cycles) with the primers 5′-TTA CGC GTA TGA CGC TGC GCG CGG-3′ and 5′-GGT CTA GAC TAC ATC TTT GAG ACC ACC G -3′ for the WT, EHmut or PTmut plasmid. *Mlu*I and *Xba*I-digested PCR products were cloned into the pCMV5 N-Flag vector. The c-Src mutant plasmid, K298M, was kindly provided by Dr. KL Guan (University of Michigan, USA).

### Protein extraction and immunoblot analysis

BAECs were lyzed with SDS lysis buffer (1% Triton, 0.1% SDS, 0.2% sodium azide, 0.5% sodium deoxycholate and proteinase inhibitors [1 mM phenylmethylsulfonyl fluoride (PMSF), 10 μg/ml aprotinin, 1 μg/ml leupeptin], then centrifuged at 12,000 *g* for 5 min and supernatant was collected. Aliquots (50 μg) of cell lysates were separated by 8% SDS-PAGE and transblotted onto Immobilon-P membrane (Millipore, Bedford, MA, USA). After being blocked with 5% skim milk in Tween/PBS, blots were incubated with primary Abs, then with horseradish peroxidase-conjugated secondary Abs. The protein bands were detected by use of an enhanced chemiluminescence kit and quantified by use of ImageQuant 5.2 (Healthcare Bio-Sciences, Philadelphia, PA).

### Immunoprecipitation (IP)

BAECs were lyzed with IP lysis buffer (50 mM Tris, pH 7.5, 5 mM EDTA, 300 mM NaCl, 1% Triton X-100, 1 mM PMSF, 10 μg/ml aprotinin, 1 μg/ml leupeptin, Tyr phosphatase cocktail I and Ser/Thr phosphatase cocktail II). After brief sonication on ice, cellular debris was removed by centrifugation at 10,000 *g* for 10 min. Aliquots (1000 mg) were incubated with anti-sEH Ab overnight at 4 °C, then 20 μL Protein A/G PLUS-Agarose was added for 2 h in 4 °C. Immune complexes were collected by centrifugation and washed 3 times with ice-cold PBS. The supernatant was discarded and the pellet was resuspended in SDS lysis buffer, then boiled with 1X SDS loading dye for 5 min. Protein was separated by SDS-PAGE and transferred to PVDF membranes and incubated with various Abs.

### Mammalian two-hybrid assay

Full-length *sEH* was obtained from mouse cDNA and sub-cloned into the pM vector (Clontech, CA, USA) with *Mlu*I and *Xba*I cutting sites. eNOS, Akt and AMPK cDNA were obtained from human cDNA and sub-cloned into the pVP16 vector (Clontech, CA, USA) with *Hind*III, *EcoR*I and *EcoR*I cutting sites, respectively, and confirmed by sequencing. Secreted human alkaline phosphatase (pG5SEAP) and plasmids were co-transfected into BAECs by use of Lipofectamin 2000 for 48 h, then treated with or without simvastatin (2.5, 5, 10 μM) for an additional 24 h. Culture media was collected and underwent chemiluminescence assays by use of GreatEscAPe SEAP chemiluminescence detection kits (Clontech, CA).

### Tube formation assay

Tube formation measurement was performed as described^45^. Briefly, ECL Cell Attachment Matrix was added to 24-well plates for overnight incubation at 37 °C. Cells were seeded onto the layer of matrix gel with indicated treatments for 4 h. Tube formation was evaluated by microscopy and quantified by counting the number of branch points in 5 random areas.

### Statistical analysis

Data are shown as mean ± SEM from 5 independent experiments. Mann-Whitney test was used to compare two independent groups and Kruskal-Wallis analysis was used with Bonferroni post-hoc correction to account for multiple testing. Statistical analysis involved use of SPSS v8.0 (SPSS Inc., Chicago, IL). *P *< 0.05 was considered statistically significant.

## Additional Information

**How to cite this article**: Hou, H.-H. *et al.* Role of phosphatase activity of soluble epoxide hydrolase in regulating simvastatin-activated endothelial nitric oxide synthase. *Sci. Rep.* **5**, 13524; doi: 10.1038/srep13524 (2015).

## Supplementary Material

Supplementary Information
