# Corrigendum: Role of phosphatase activity of soluble epoxide hydrolase in regulating simvastatin-activated endothelial nitric oxide synthase

**DOI:** 10.1038/srep46877

**Published:** 2017-07-31

**Authors:** Hsin-Han Hou, Yi-Jen Liao, Sheng-Huang Hsiao, Song-Kun Shyue, Tzong-Shyuan Lee

Scientific Reports 5: Article number: 13524; 10.1038/srep13524 published online: 08 25 2015; updated: 07 31 2017.

The original version of this Article contained an error in Figure 4b, where the ‘AMPK’ and ‘p-AMPK’ labels were inverted. In addition, the blot for AMPK in the vector group was incorrect.

The Supplementary Information file that accompanies the Article has now been updated on page 37 with the unprocessed blot corresponding to Figure 4.

These errors have now been corrected in the PDF and HTML versions of this Article.

