# Supplementary material for: Role of phosphatase activity of soluble epoxide hydrolase in regulating simvastatin-activated endothelial nitric oxide synthase
Source: Sci Rep. 2015 Aug 25;5:13524. doi: 10.1038/srep13524 (PMC4548251; doi:10.1038/srep13524)
Supplement: Supplementary Information [file srep13524-s1.pdf]

Supplementary Information

Role of phosphatase activity of soluble epoxide hydrolase in regulating simvastatin-activated endothelial nitric oxide synthase

Hsin-Han Hou, Yi-Jen Liao, Sheng-Huang Hsiao, Song-Kun Shyue, Tzong-Shyuan Lee

Fig S3. **Role of sEH phosphatase in regulating simvastatin-induced eNOS phosphorylation.**

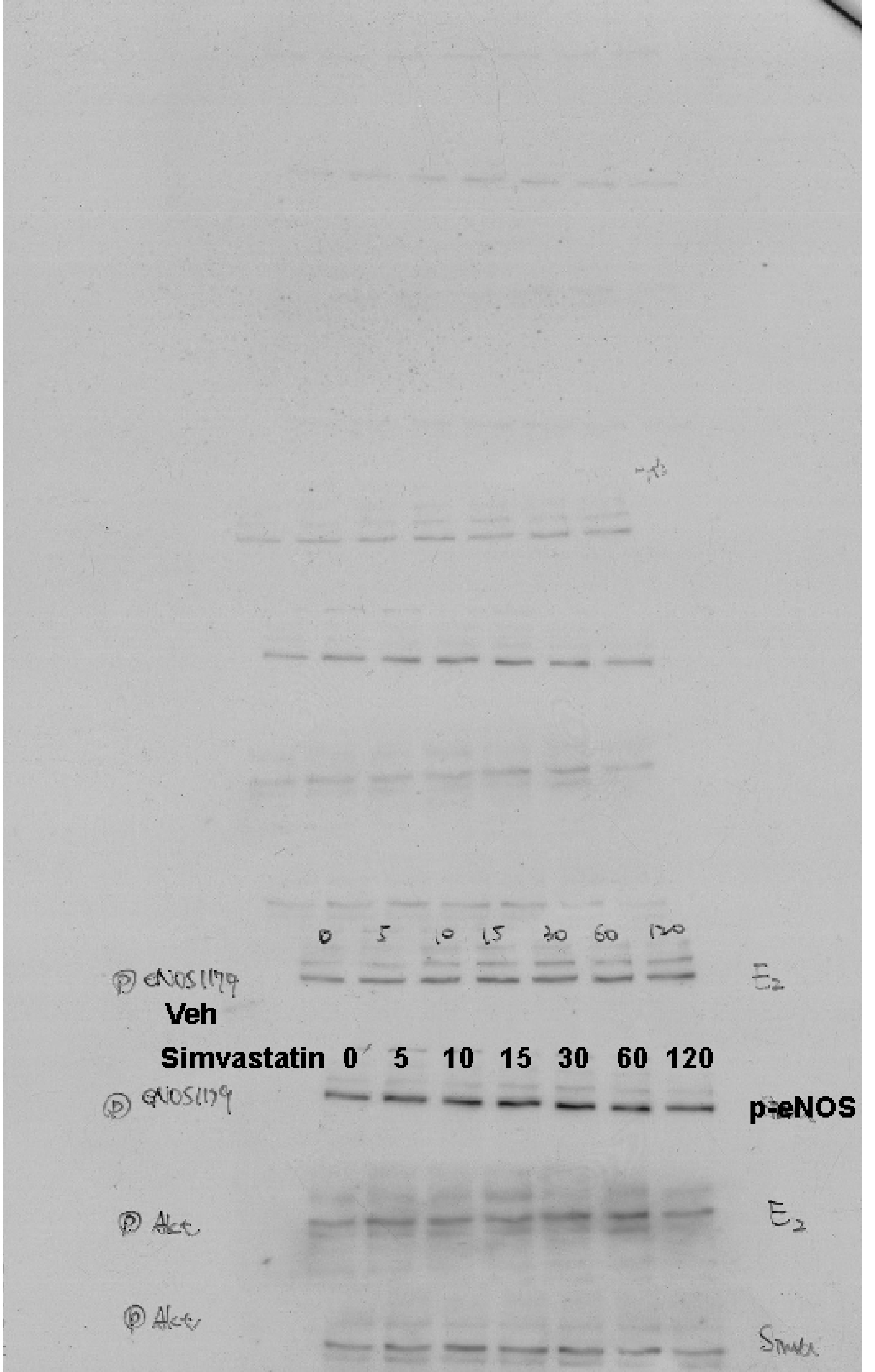
0 5 10 15 30 60 120
E2
Veh
Simvastatin 0 5 10 15 30 60 120
p-eNOS
E2

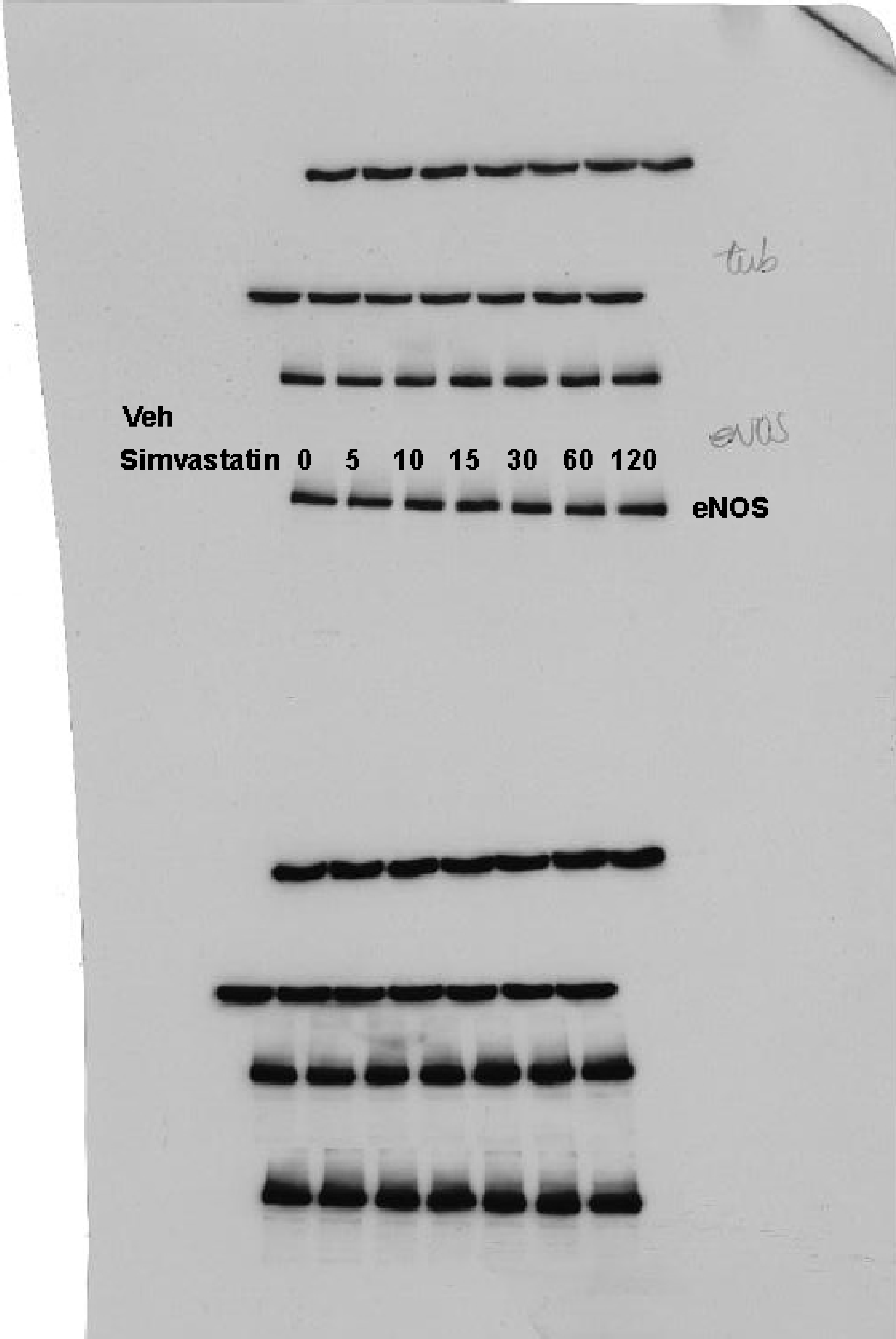
tub
Veh
eNOS
Simvastatin 0 5 10 15 30 60 120
eNOS

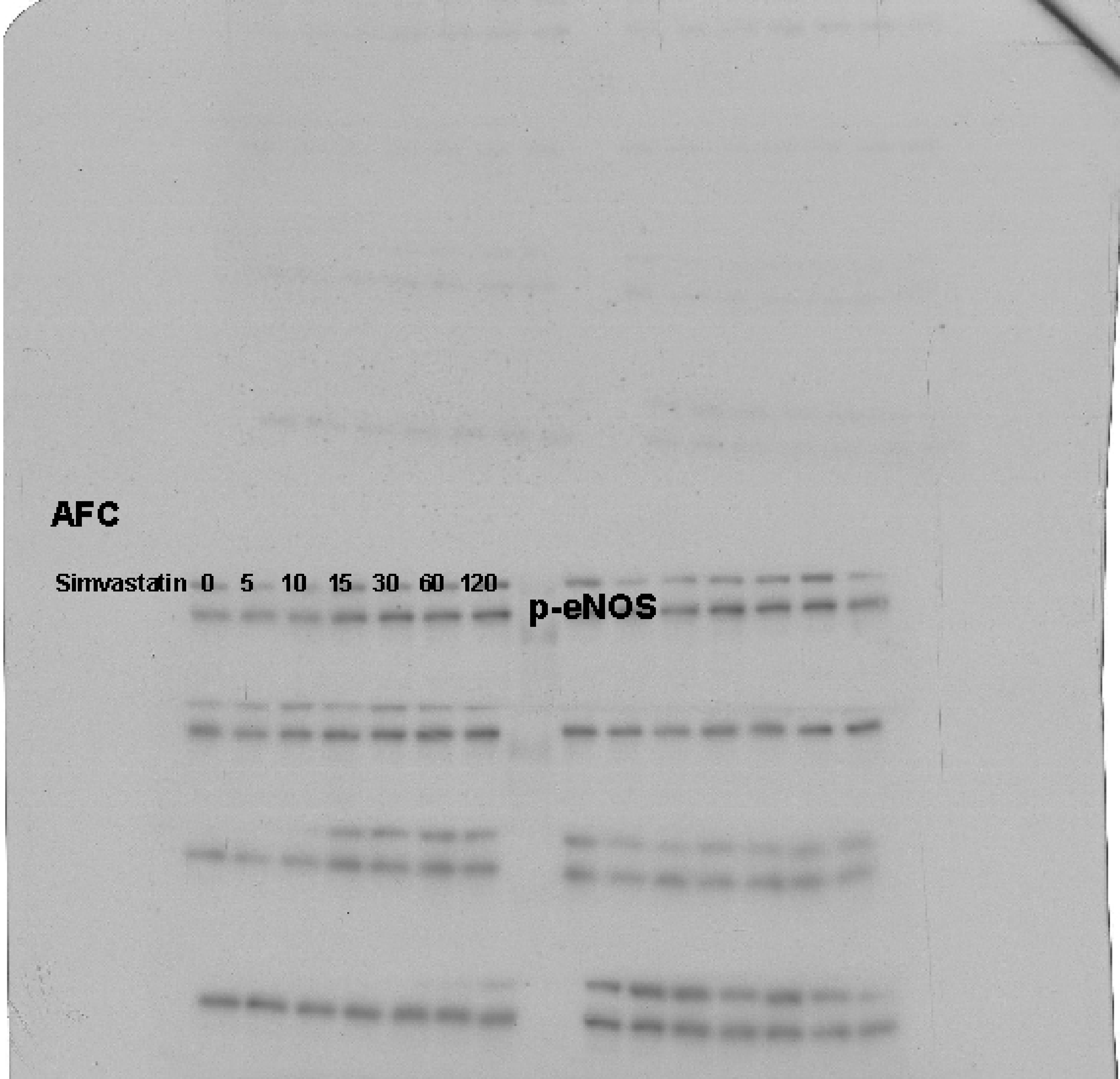
AFC
Simvastatin 0 5 10 15 30 60 120
p-eNOS

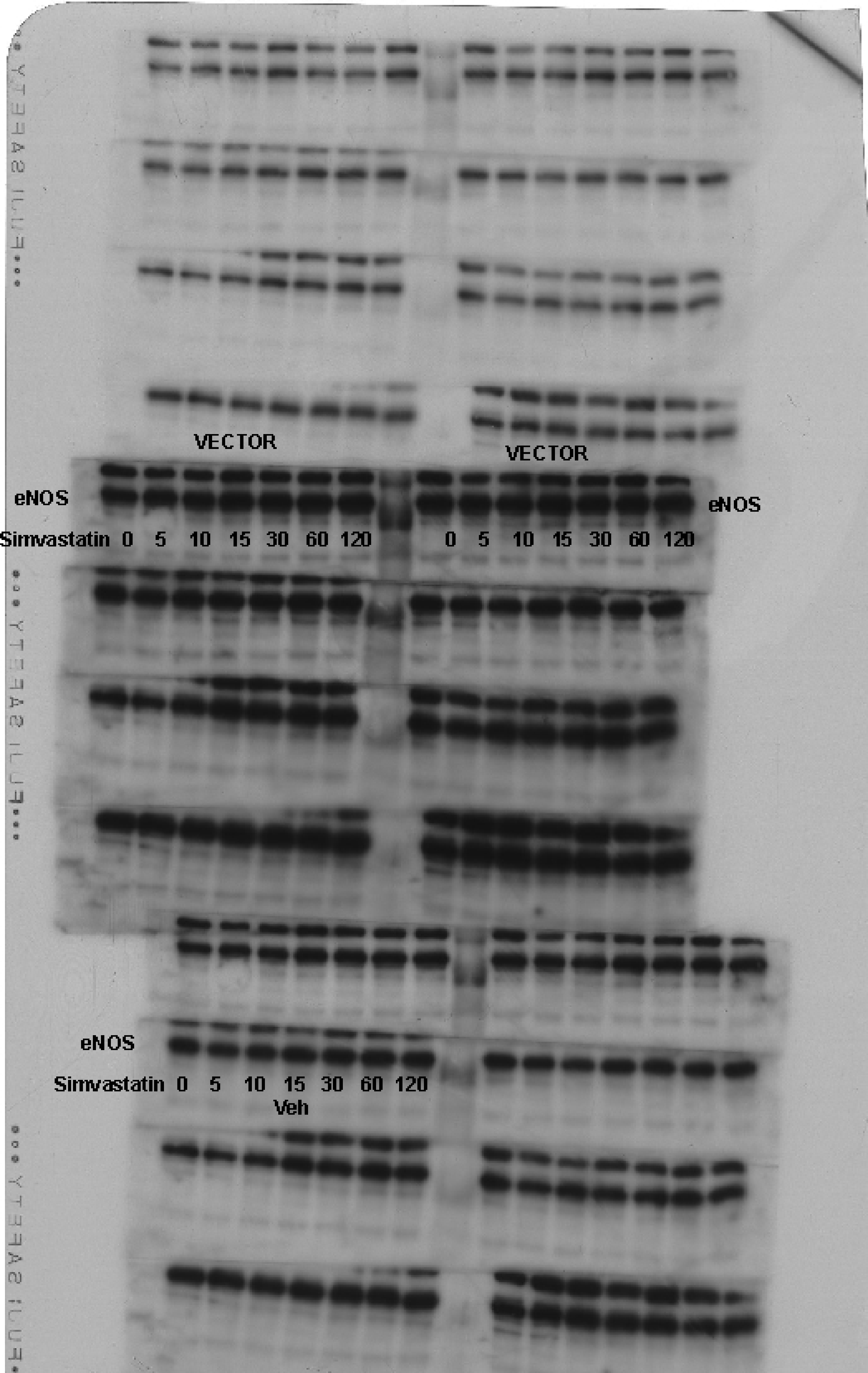
VECTOR
VECTOR
eNOS
Simvastatin 0 5 10 15 30 60 120
0 5 10 15 30 60 120
eNOS
eNOS
Simvastatin 0 5 10 15 30 60 120
Veh

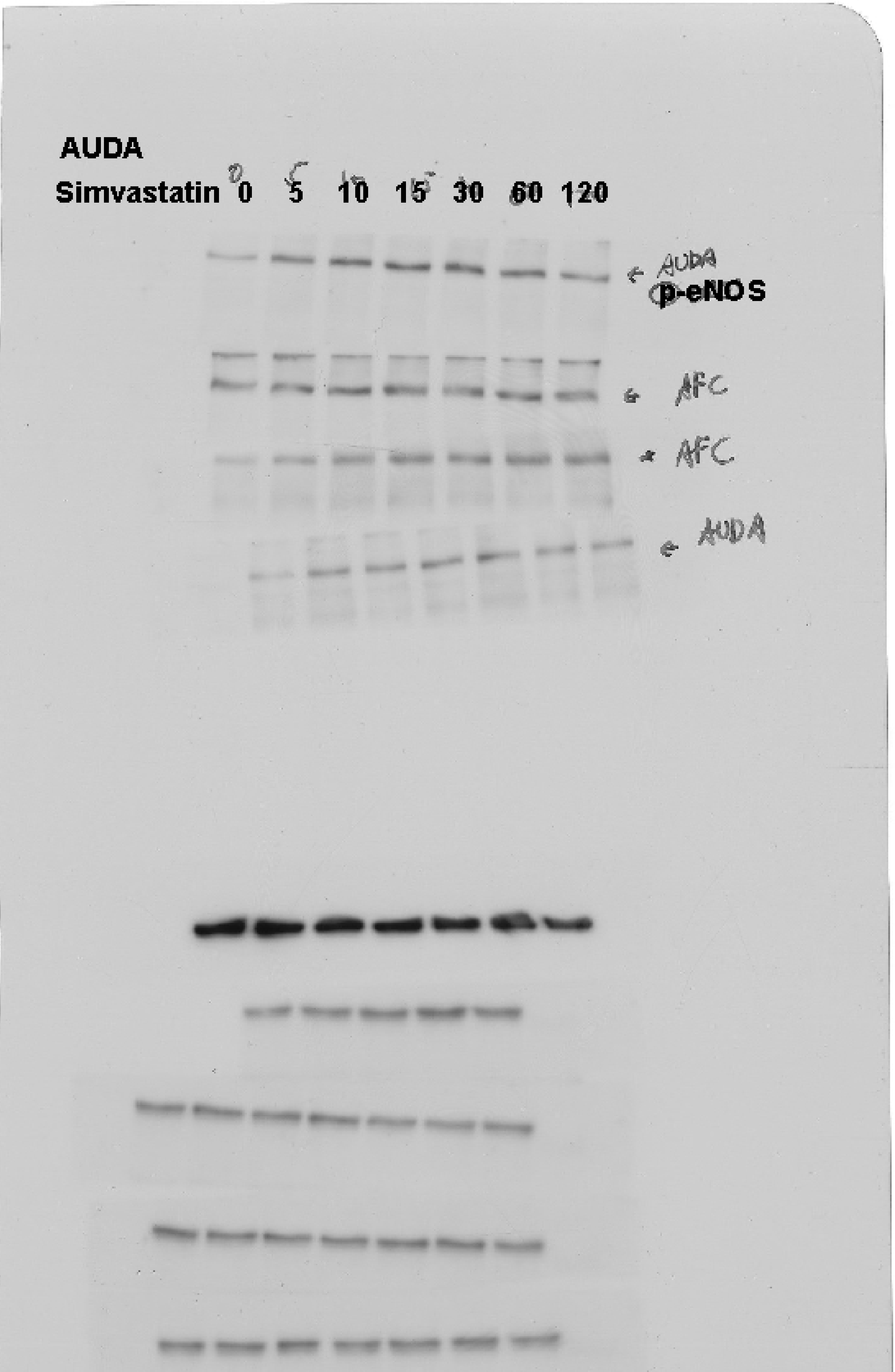
AUDA
Simvastatin 0 5 10 15 30 60 120
AUDA
p-eNOS
AFC
AFC
AUDA

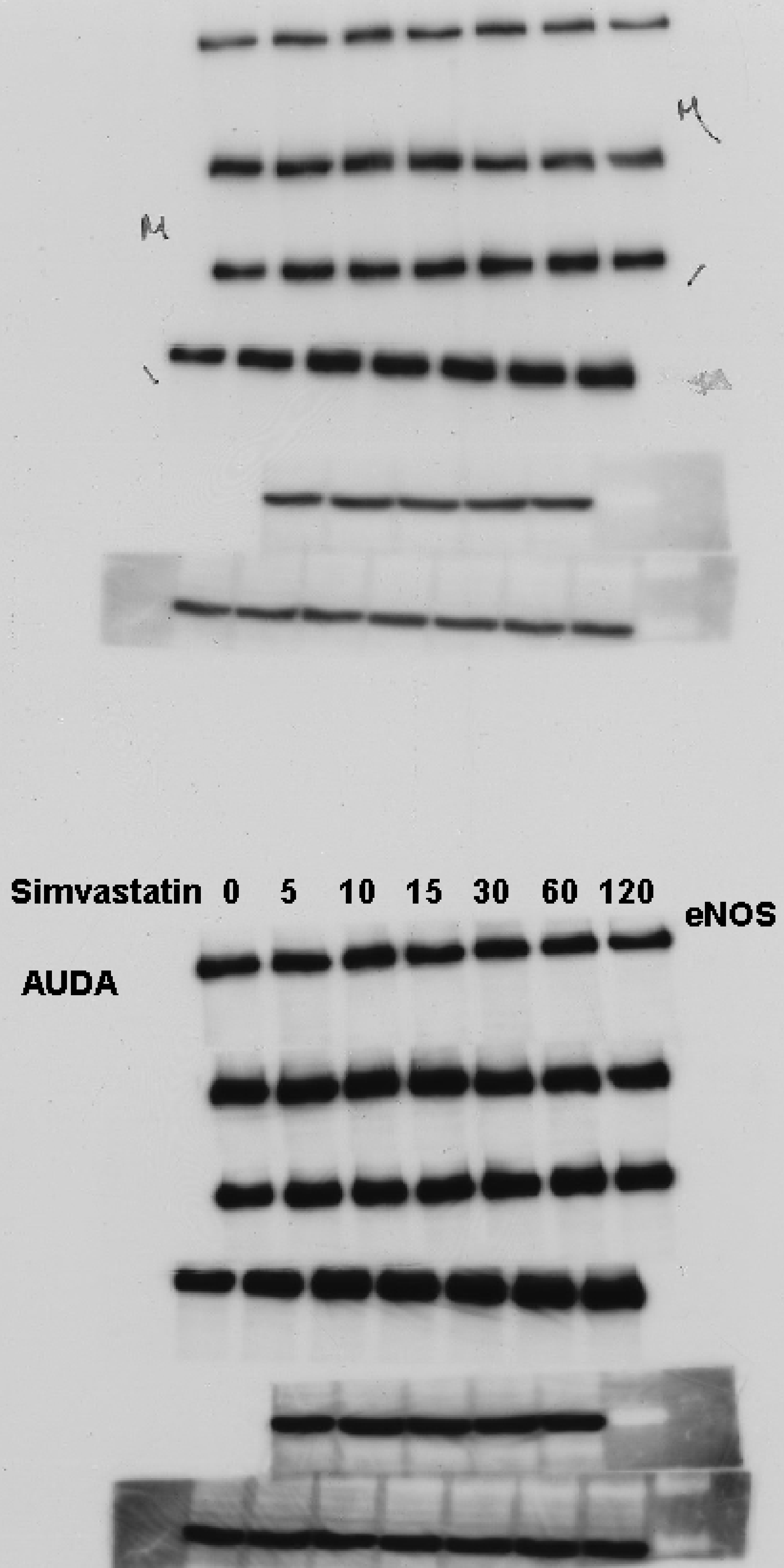
Simvastatin 0 5 10 15 30 60 120
eNOS
AUDA

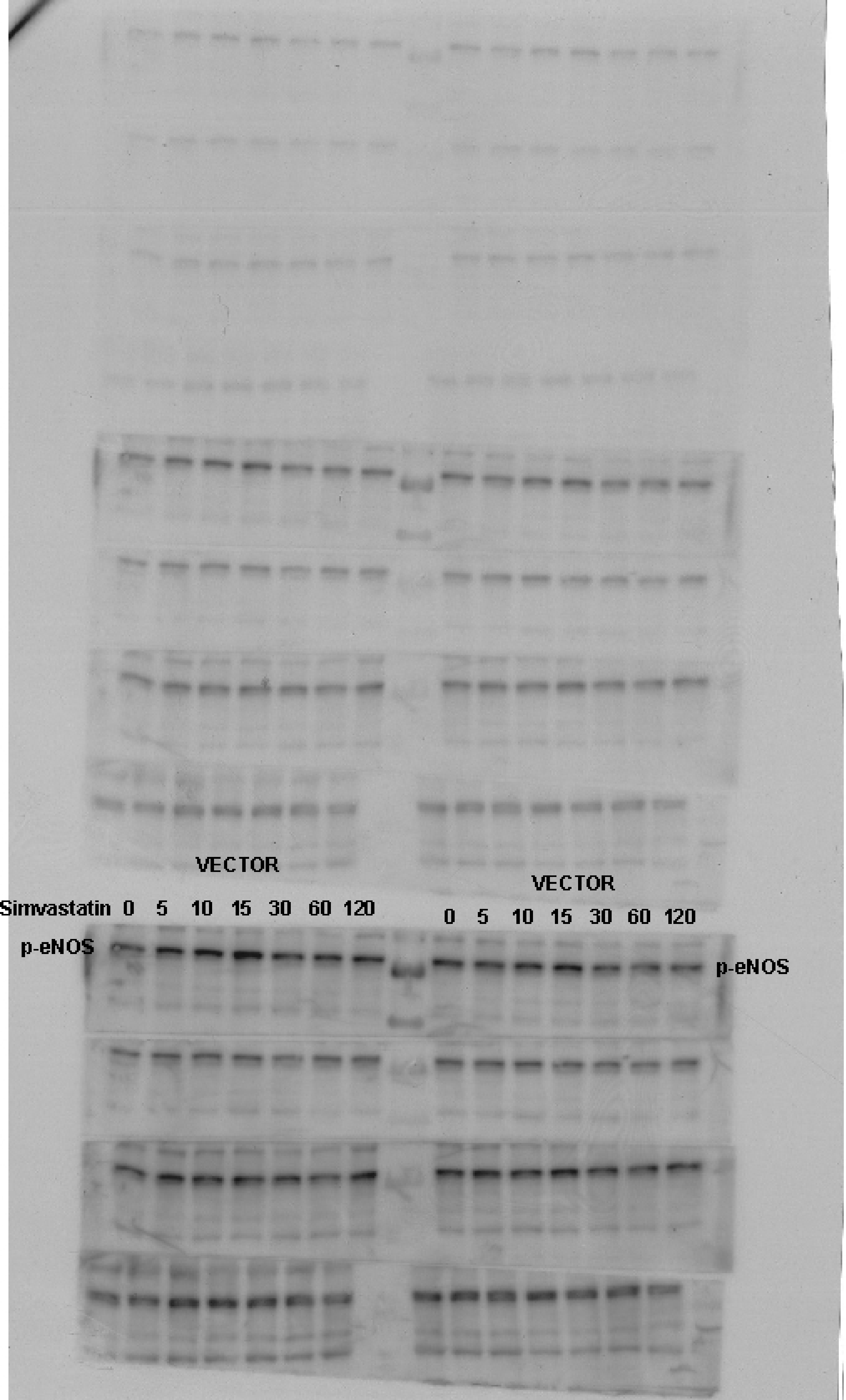
VECTOR
VECTOR
Simvastatin 0 5 10 15 30 60 120
0 5 10 15 30 60 120
p-eNOS
p-eNOS

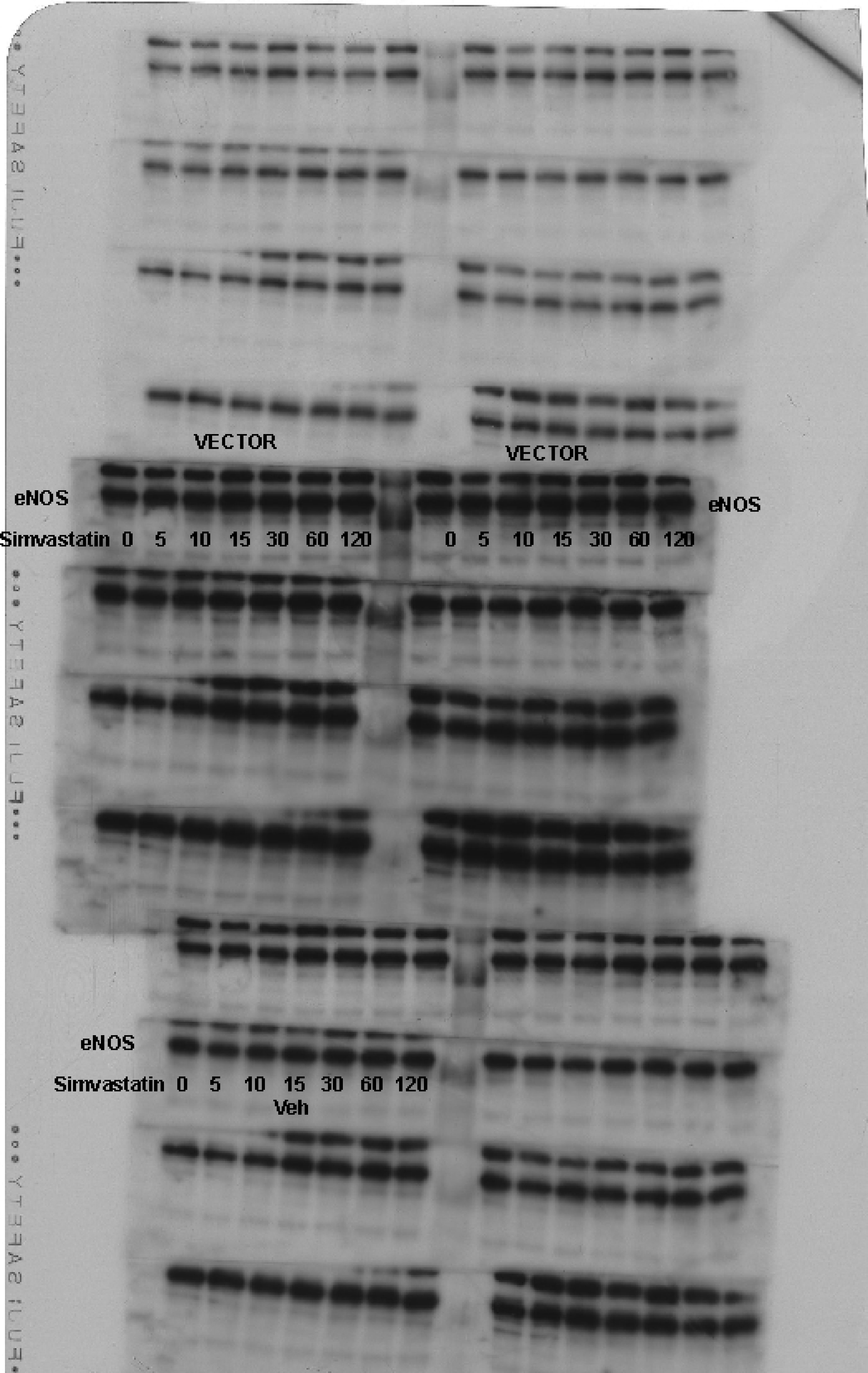
VECTOR
VECTOR
eNOS
eNOS
Simvastatin 0 5 10 15 30 60 120
0 5 10 15 30 60 120
eNOS
Simvastatin 0 5 10 15 30 60 120
Veh

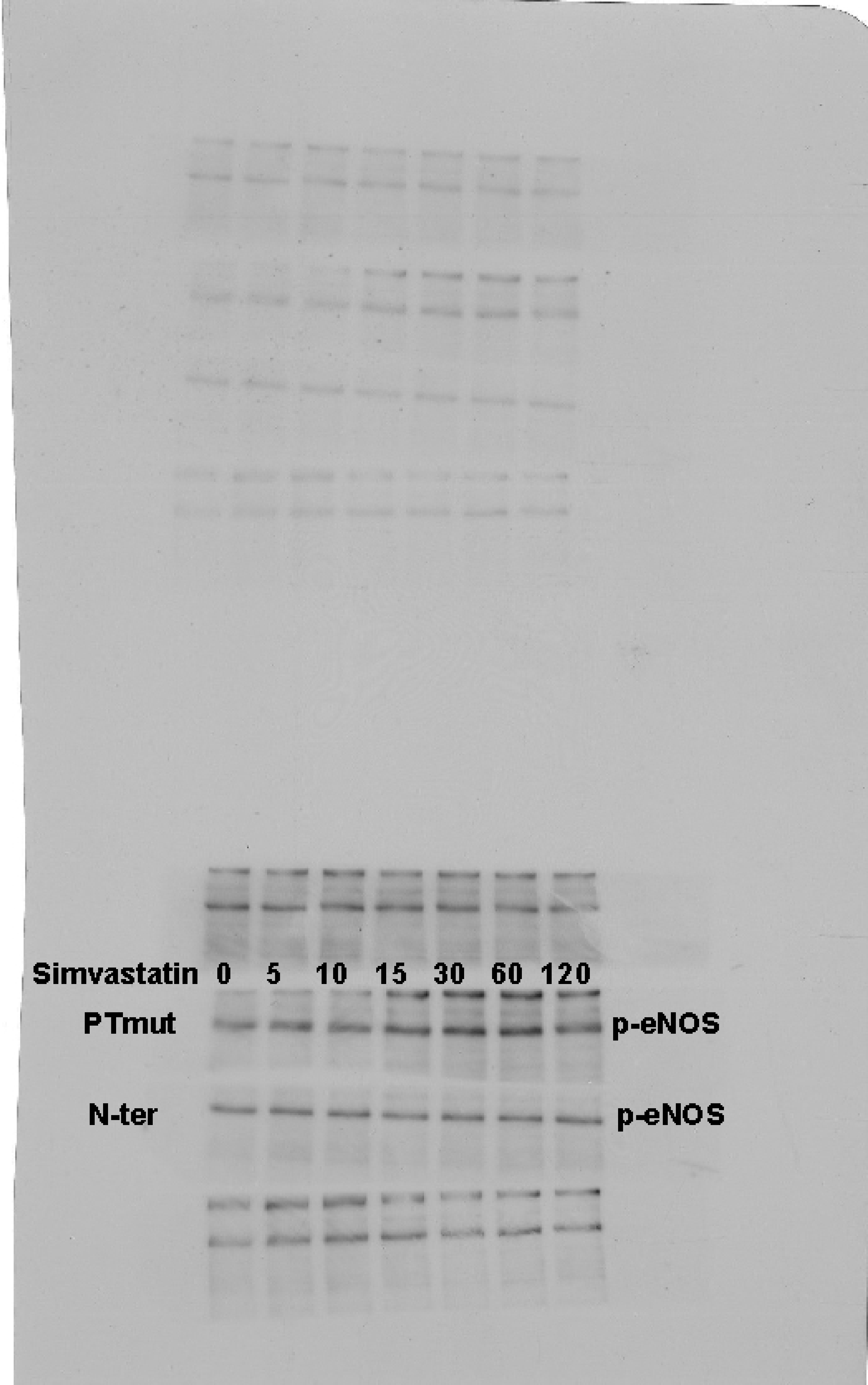
Simvastatin 0 5 10 15 30 60 120
PTmut
p-eNOS
N-ter
p-eNOS

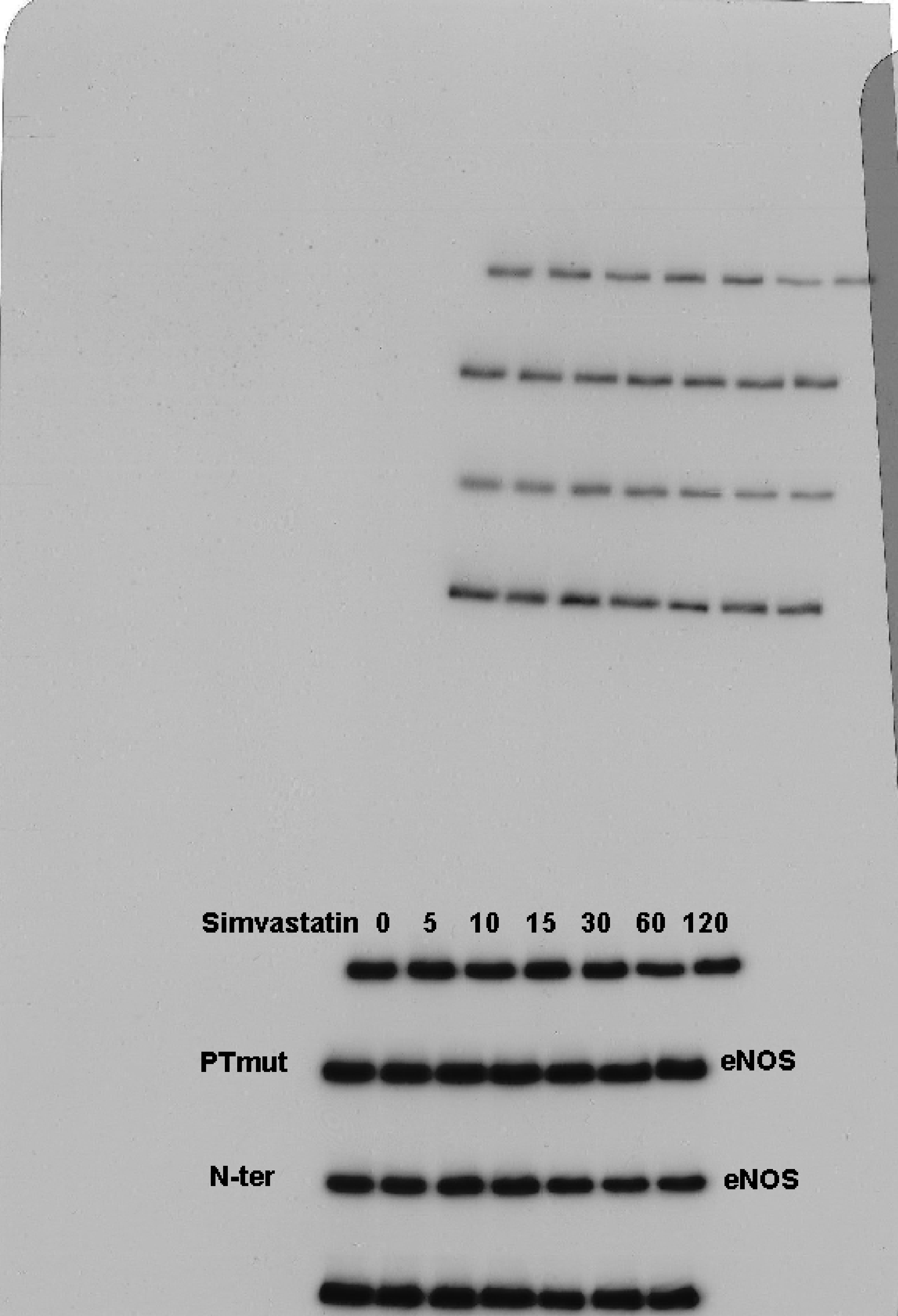
Simvastatin 0 5 10 15 30 60 120
PTmut
eNOS
N-ter
eNOS

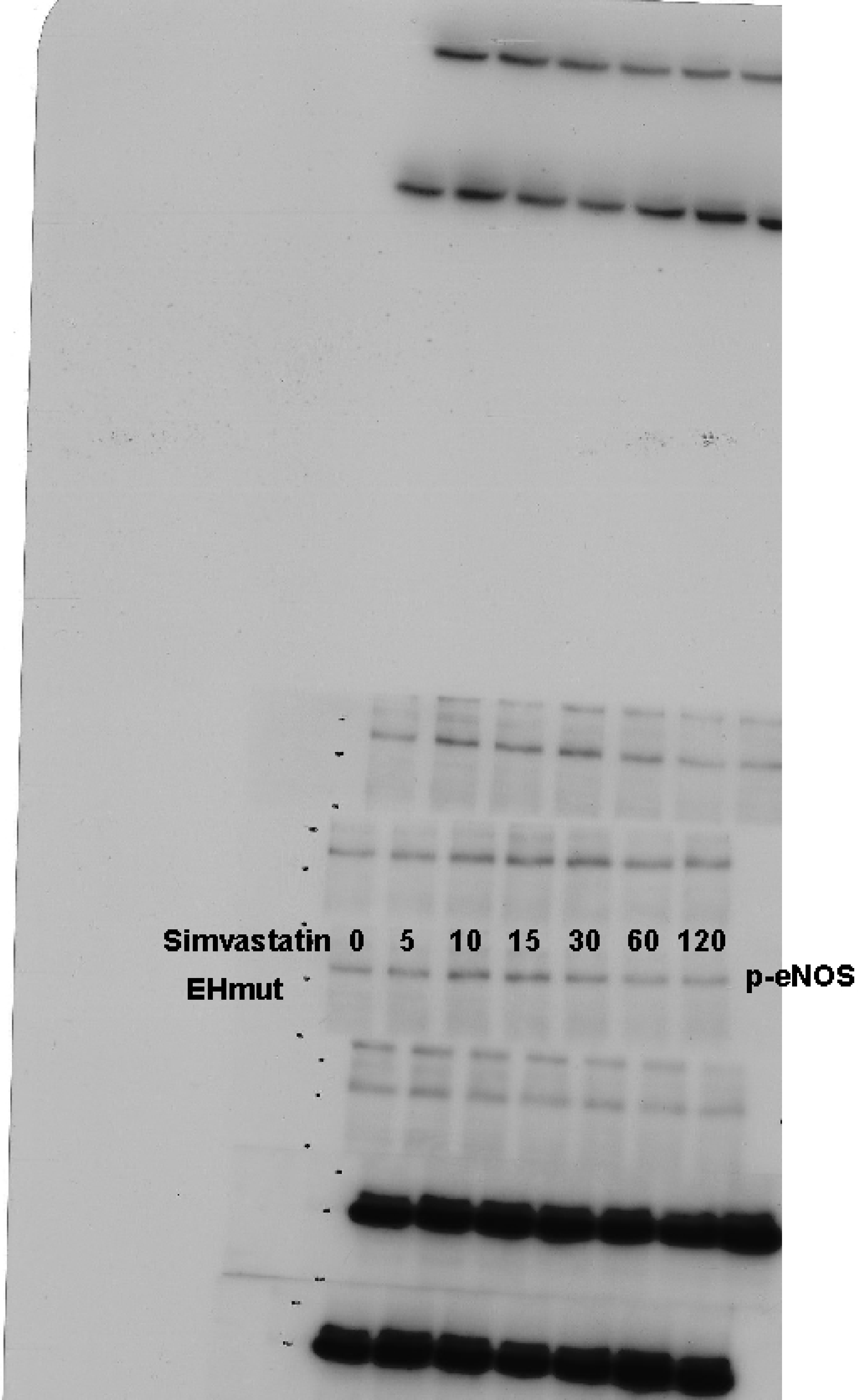
Simvastatin 0 5 10 15 30 60 120
EHmut
p-eNOS

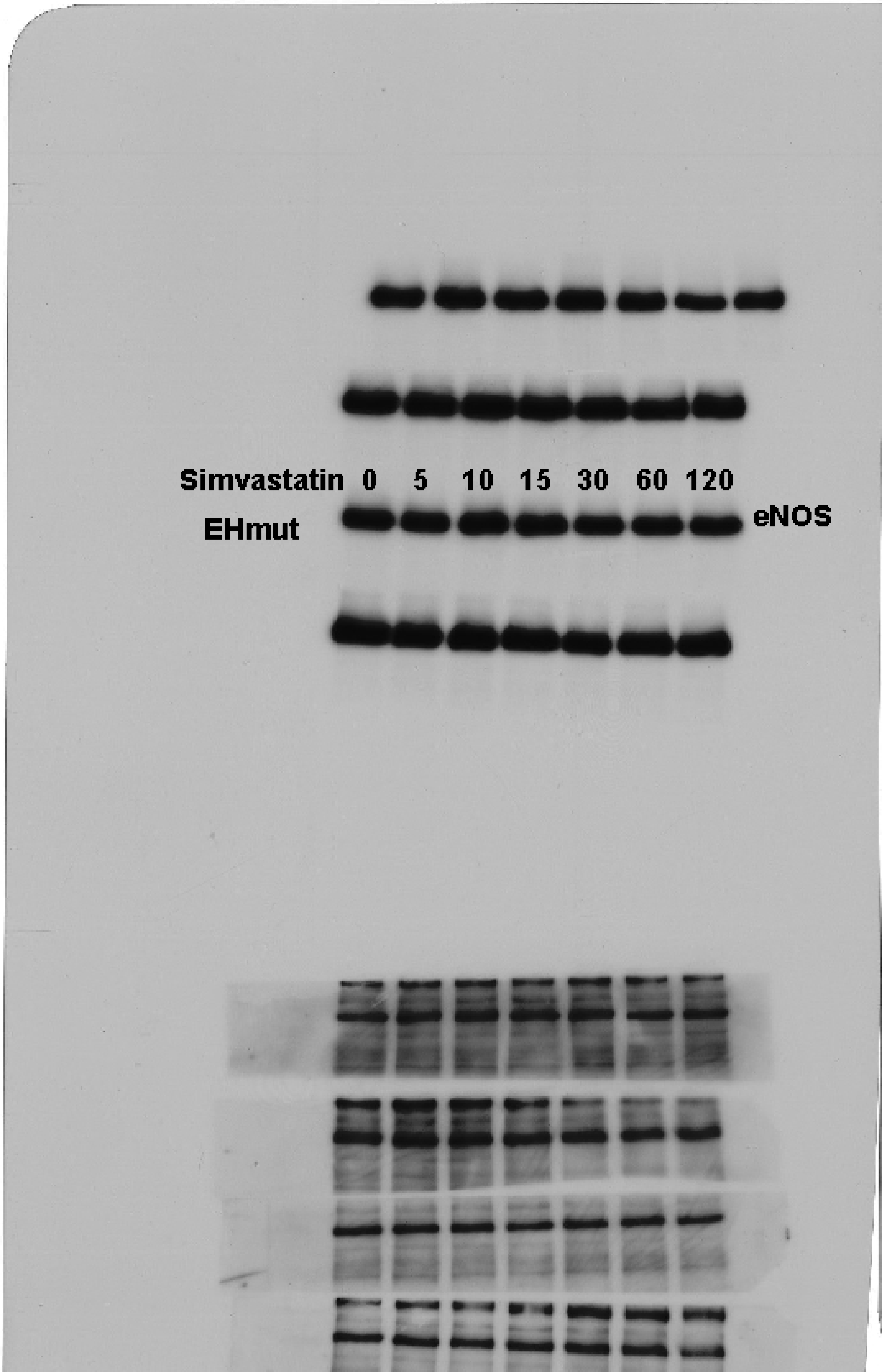
Simvastatin 0 5 10 15 30 60 120
EHmut
eNOS

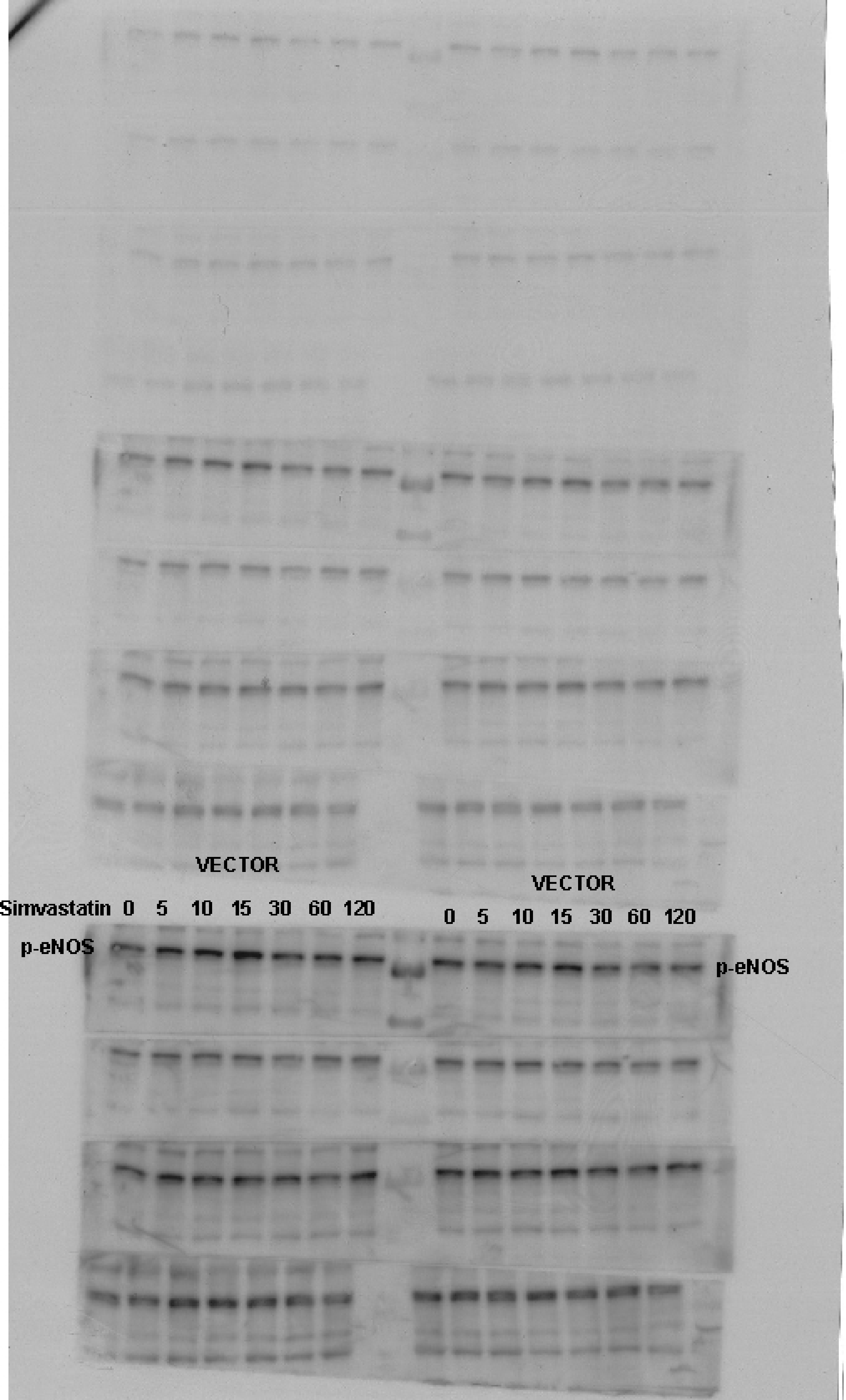

VECTOR
VECTOR
Simvastatin 0 5 10 15 30 60 120
0 5 10 15 30 60 120
p-eNOS
p-eNOS

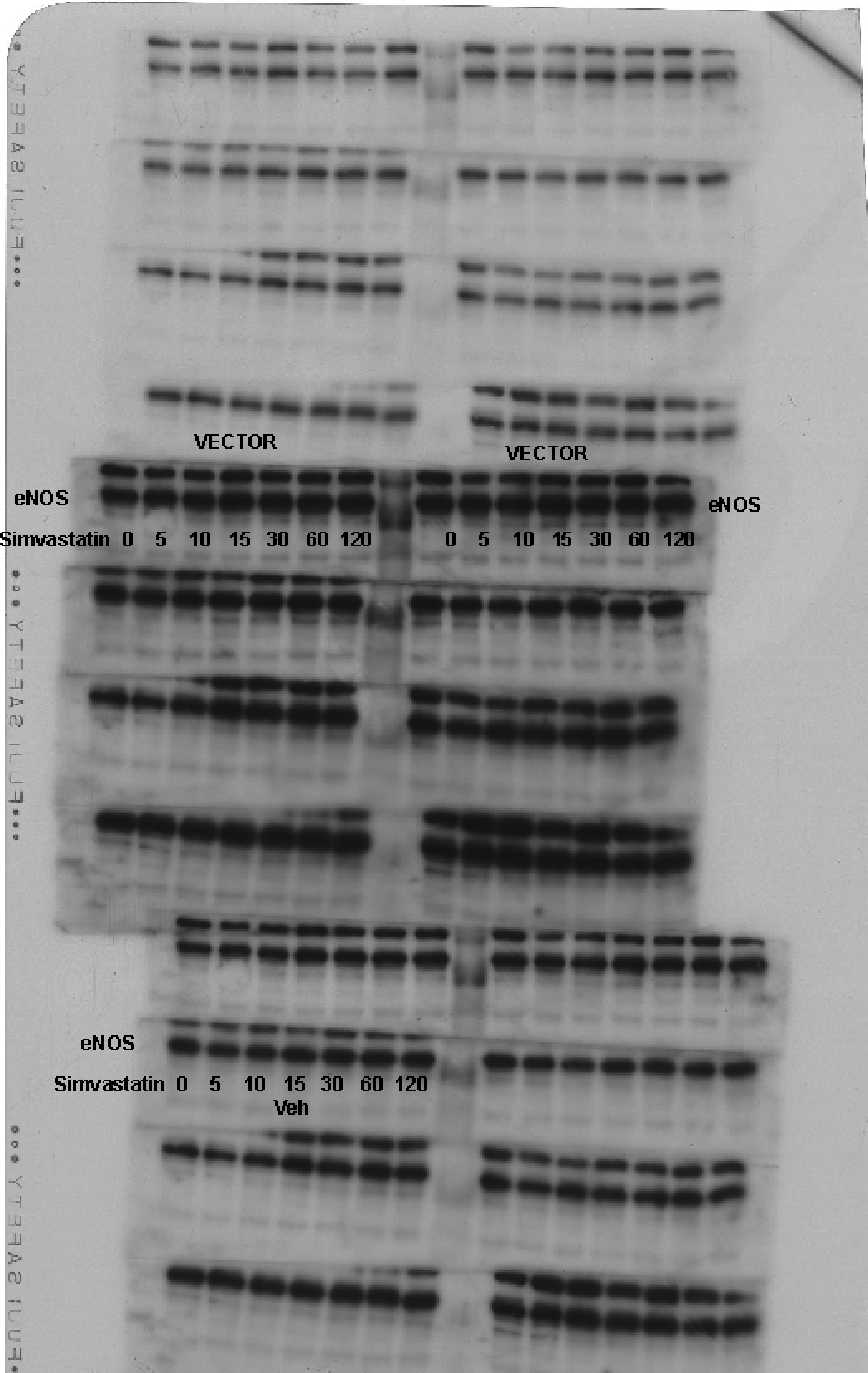
VECTOR
VECTOR
eNOS
eNOS
Simvastatin 0 5 10 15 30 60 120
0 5 10 15 30 60 120
eNOS
Simvastatin 0 5 10 15 30 60 120
Veh

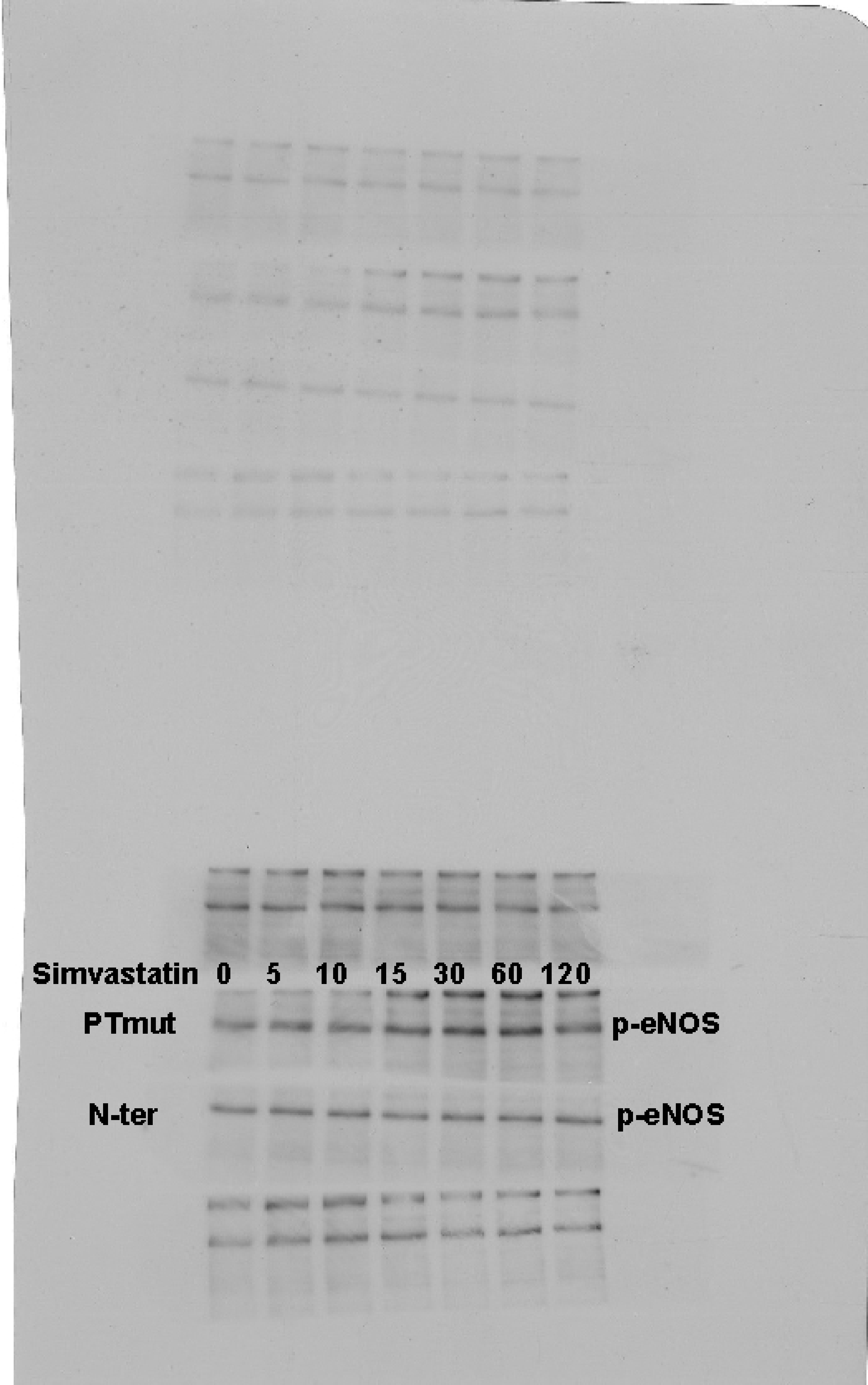

Simvastatin 0 5 10 15 30 60 120
PTmut
p-eNOS
N-ter
p-eNOS

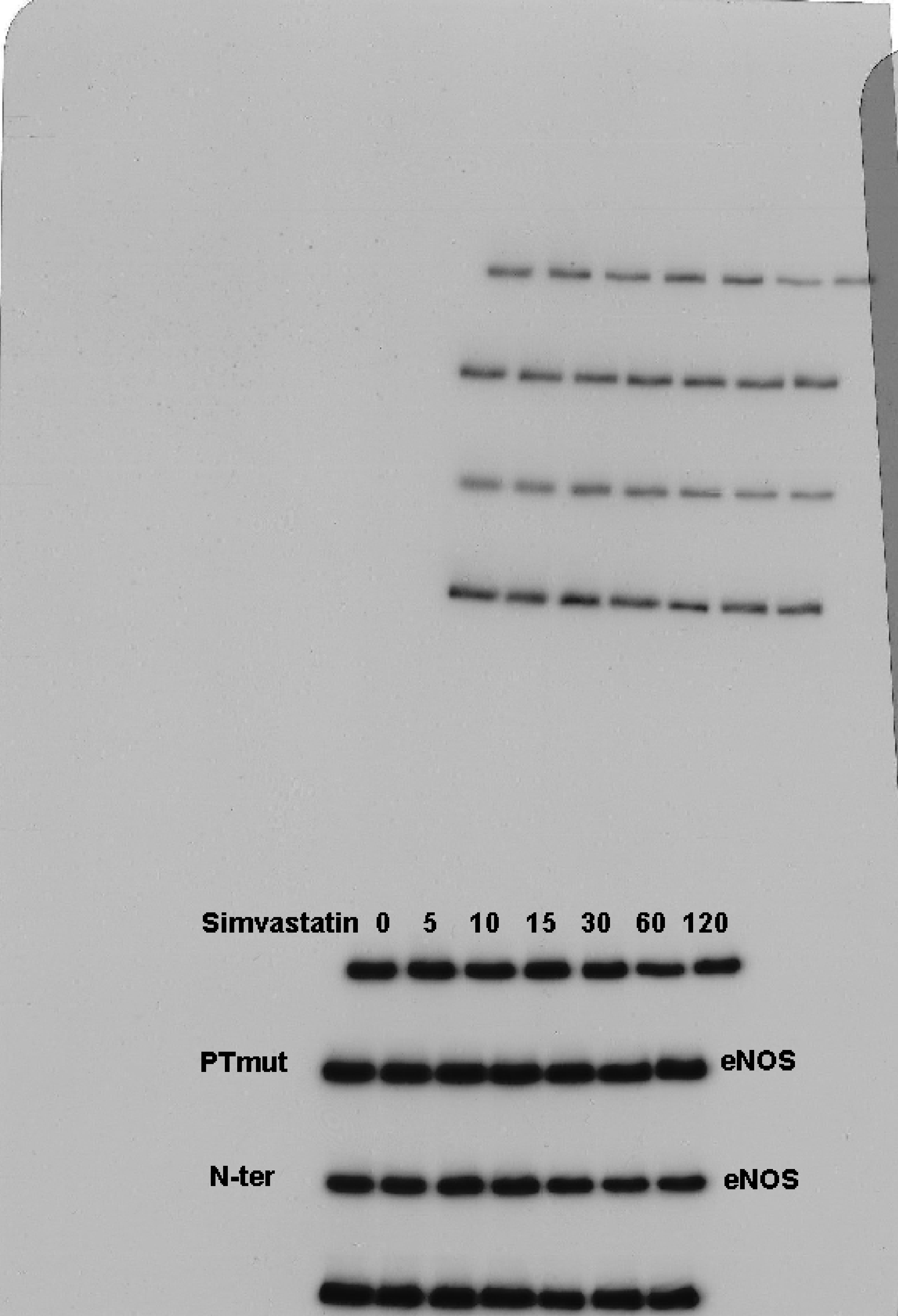

Simvastatin 0 5 10 15 30 60 120
PTmut
eNOS
N-ter
eNOS

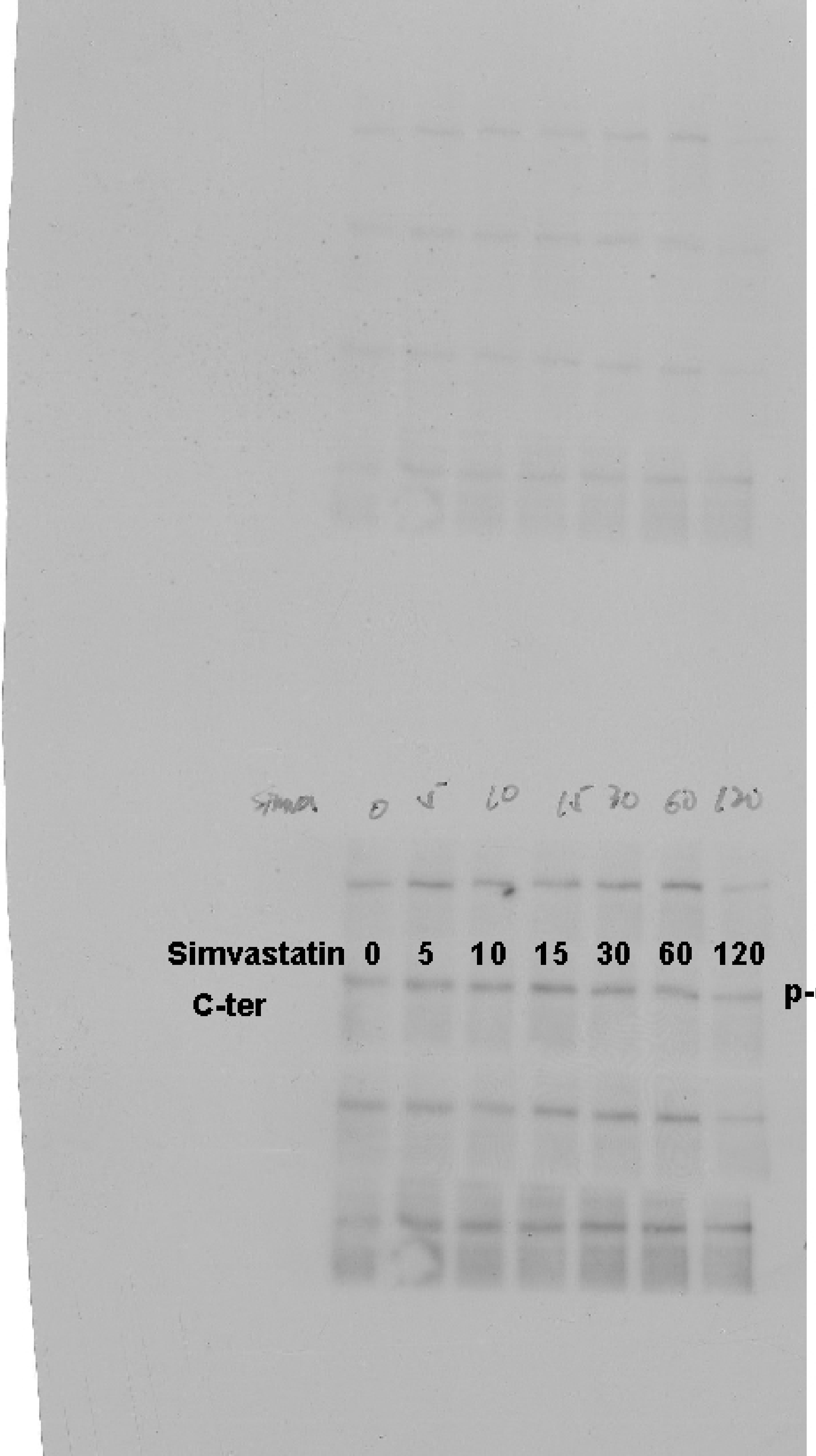

Simvastatin 0 5 10 15 30 60 120
C-ter
p-eNOS

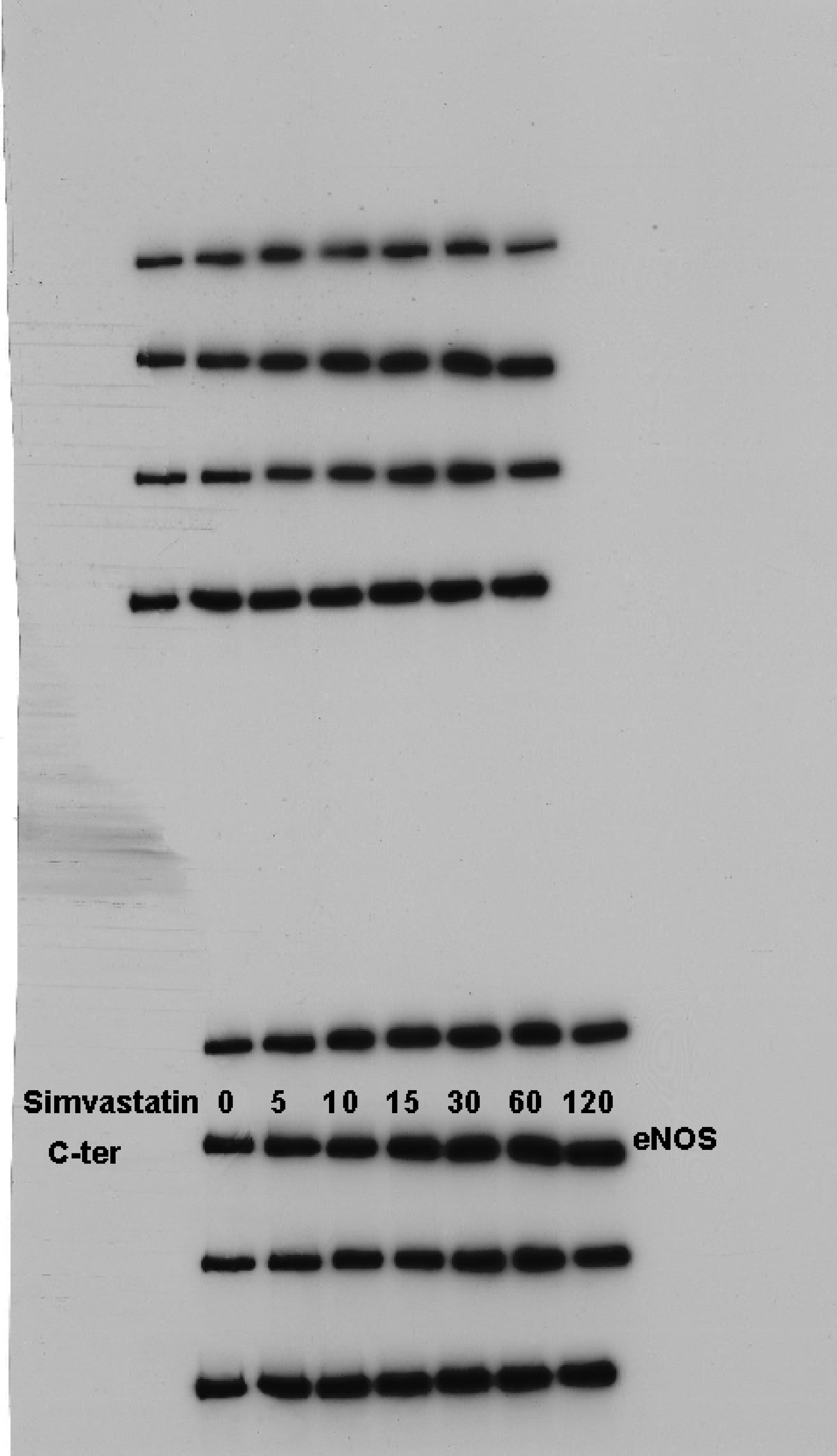
Simvastatin 0 5 10 15 30 60 120
C-ter
eNOS

Fig S4. **sEH phosphatase plays a crucial role in regulating simvastatin-induced Akt and AMPK phosphorylation.**

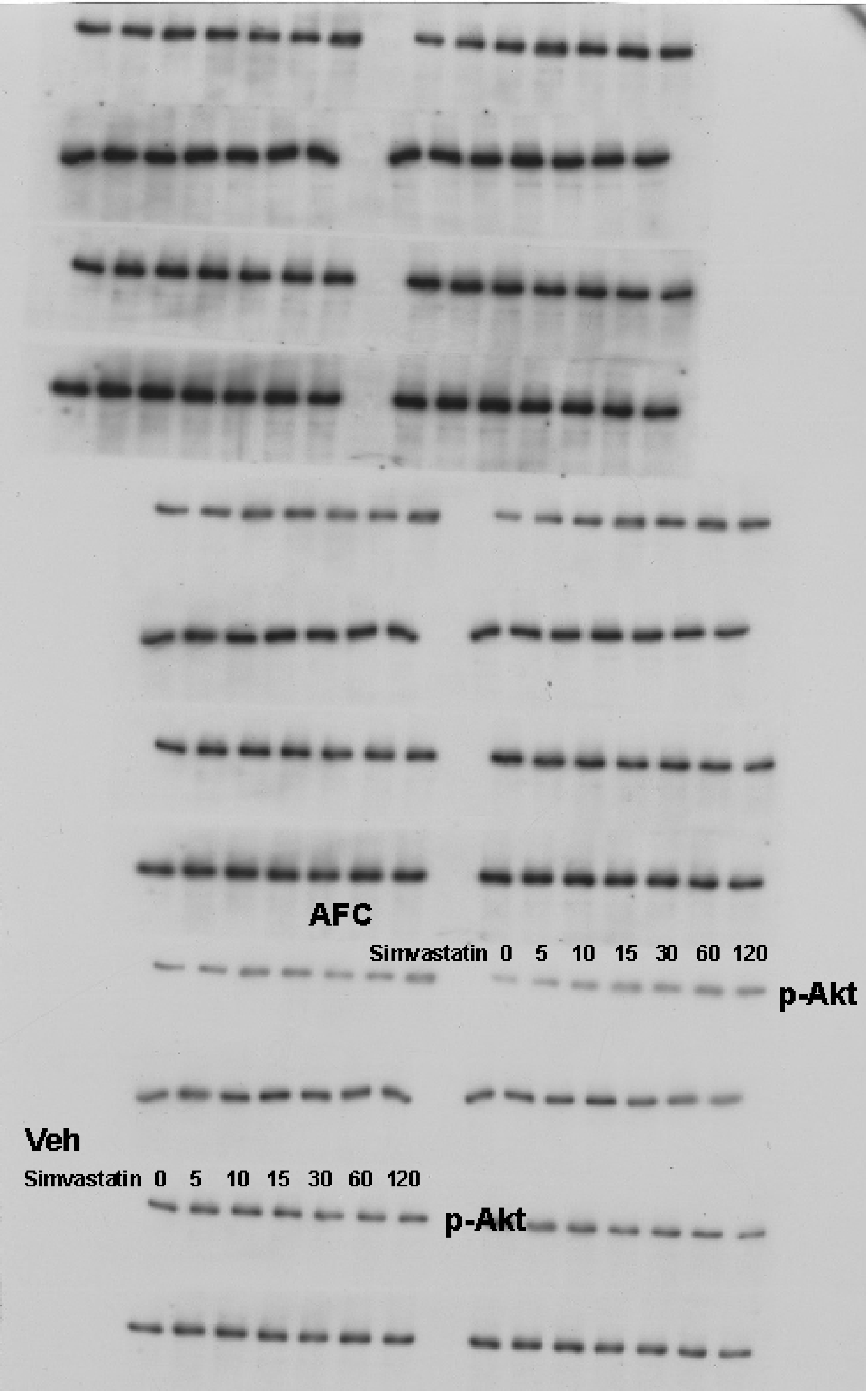
AFC
Simvastatin 0 5 10 15 30 60 120
p-Akt
Veh
Simvastatin 0 5 10 15 30 60 120
p-Akt

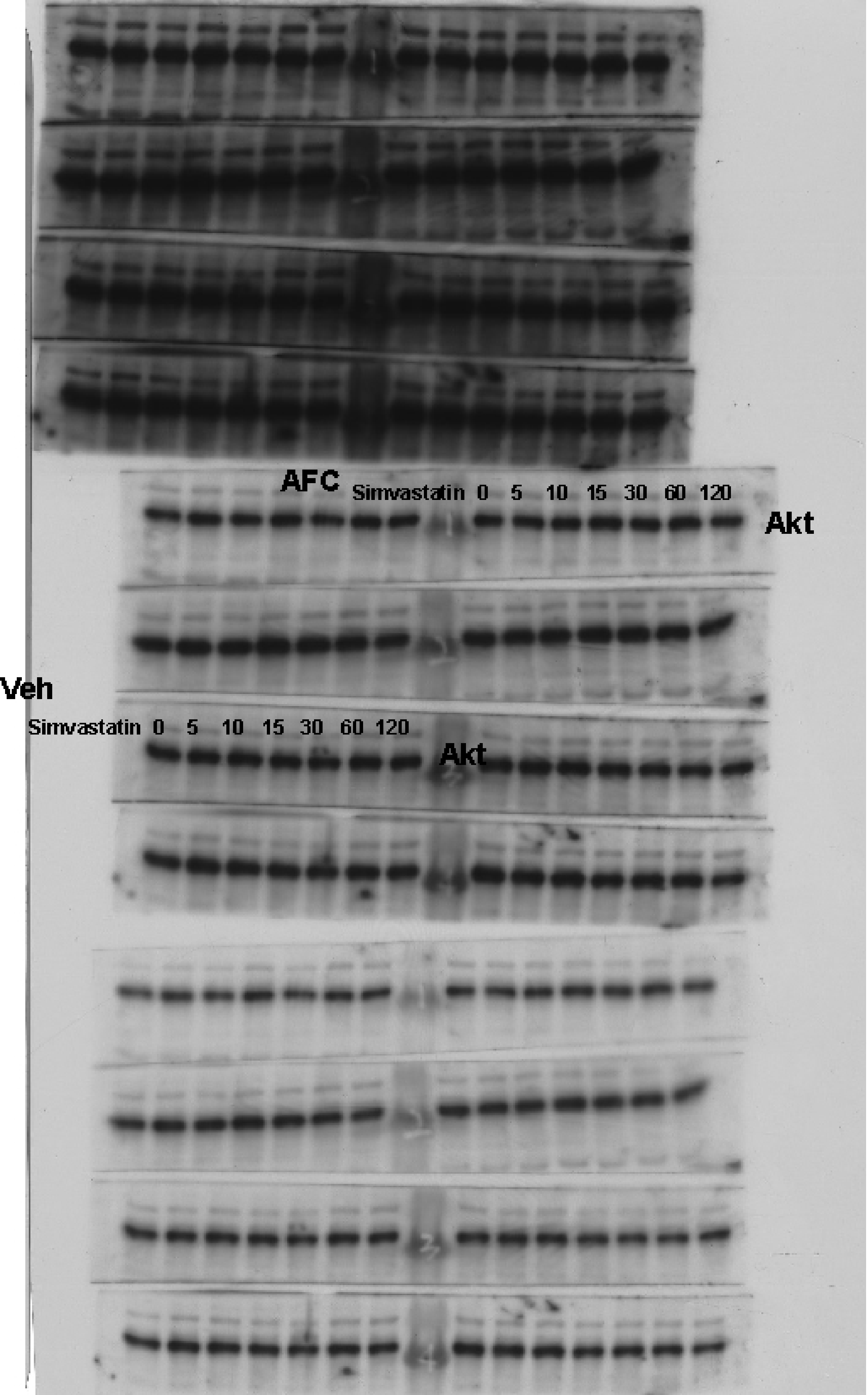
AFC
Simvastatin 0 5 10 15 30 60 120
Akt
Veh
Simvastatin 0 5 10 15 30 60 120
Akt

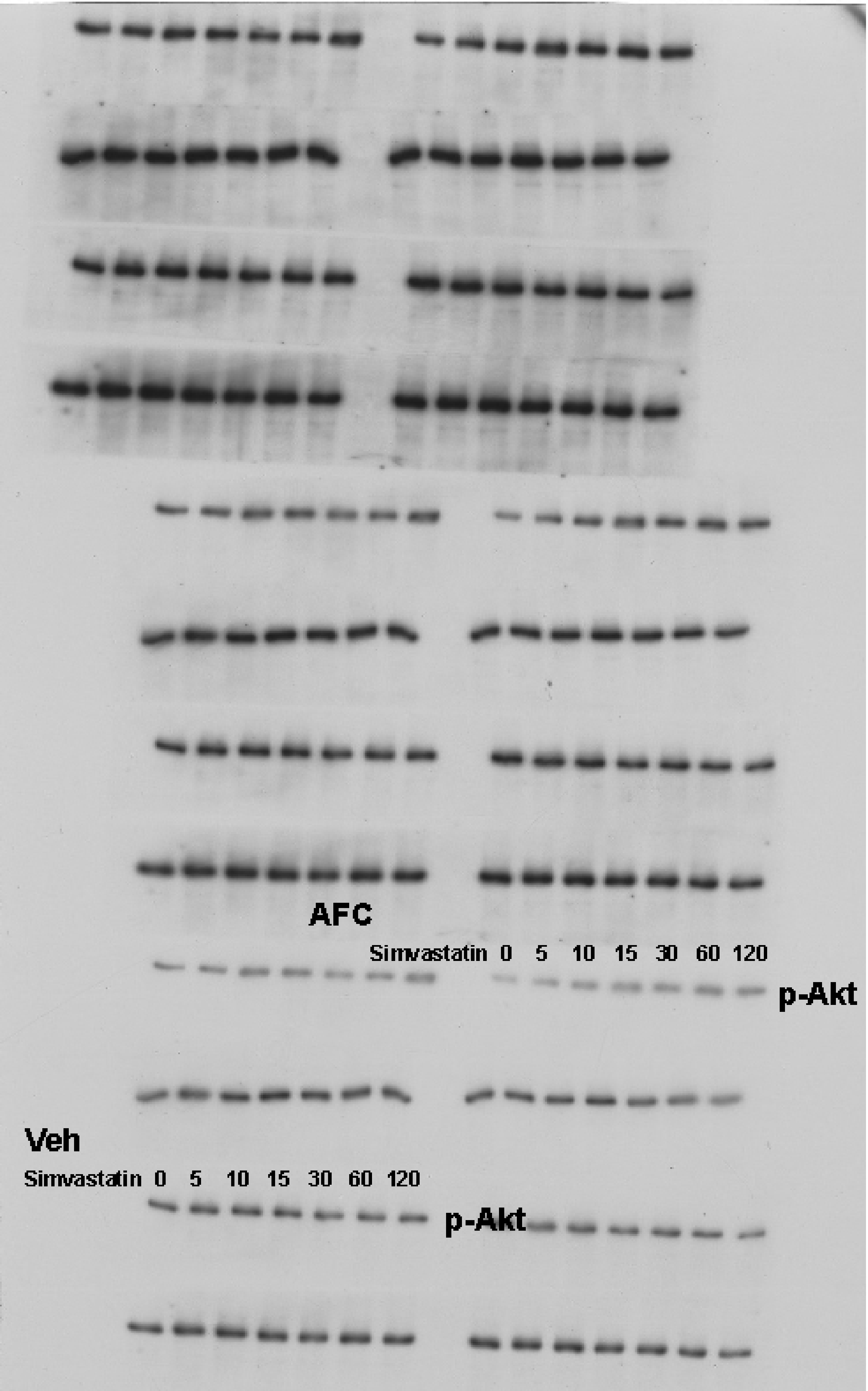
AFC
Simvastatin 0 5 10 15 30 60 120
p-Akt
Veh
Simvastatin 0 5 10 15 30 60 120
p-Akt

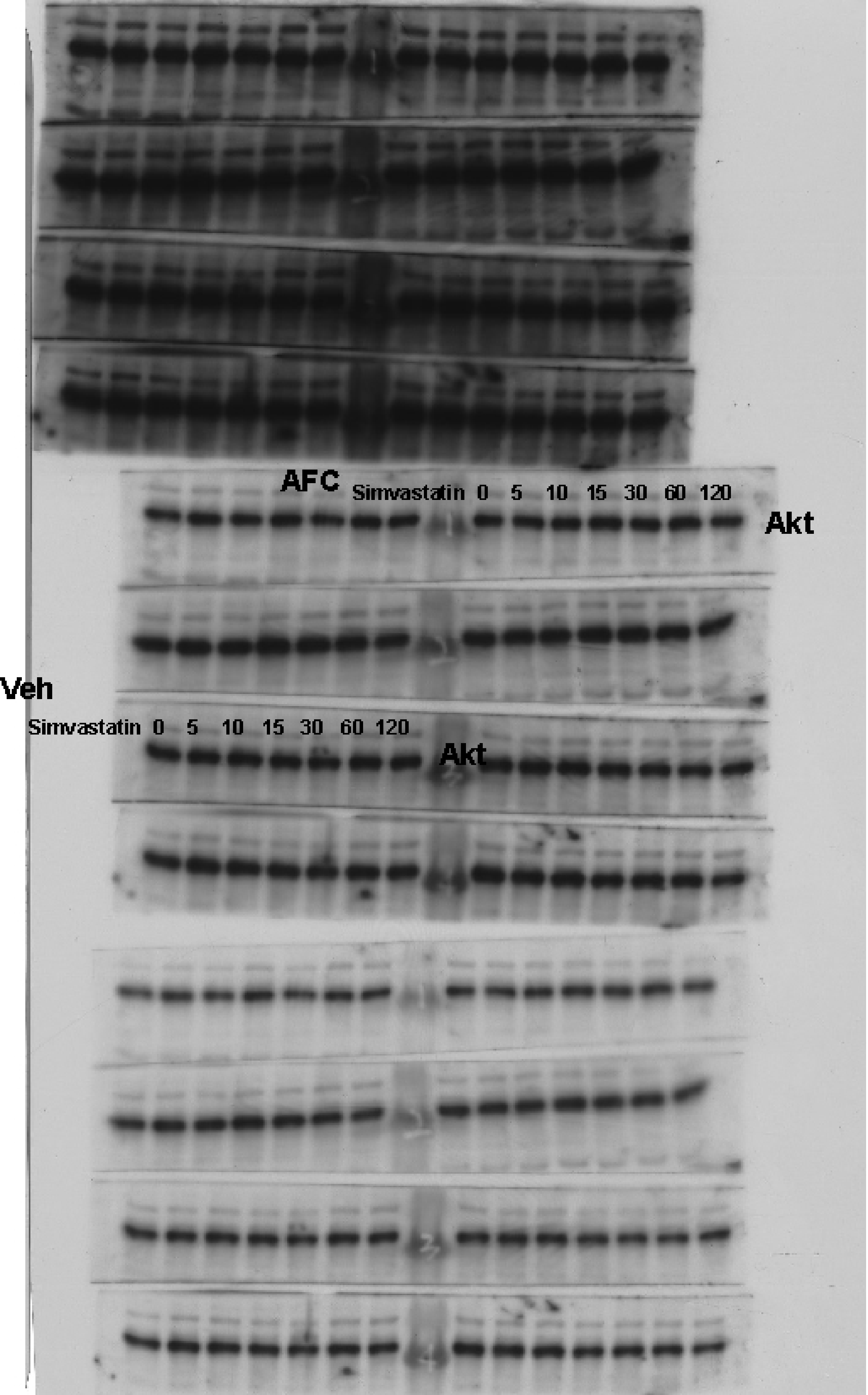
AFC
Simvastatin 0 5 10 15 30 60 120
Akt
Veh
Simvastatin 0 5 10 15 30 60 120
Akt

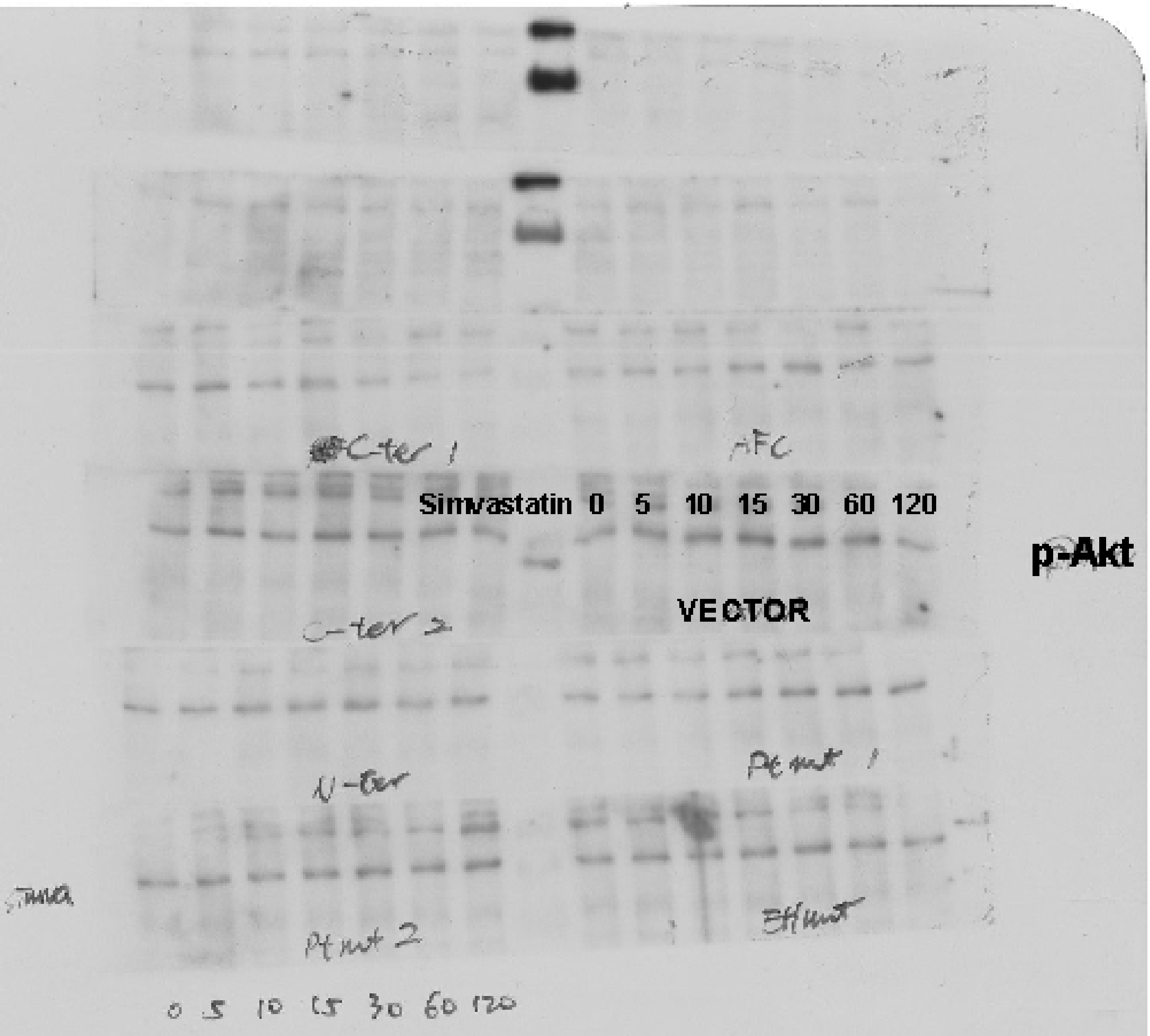
Simvastatin 0 5 10 15 30 60 120
p-Akt
VECTOR

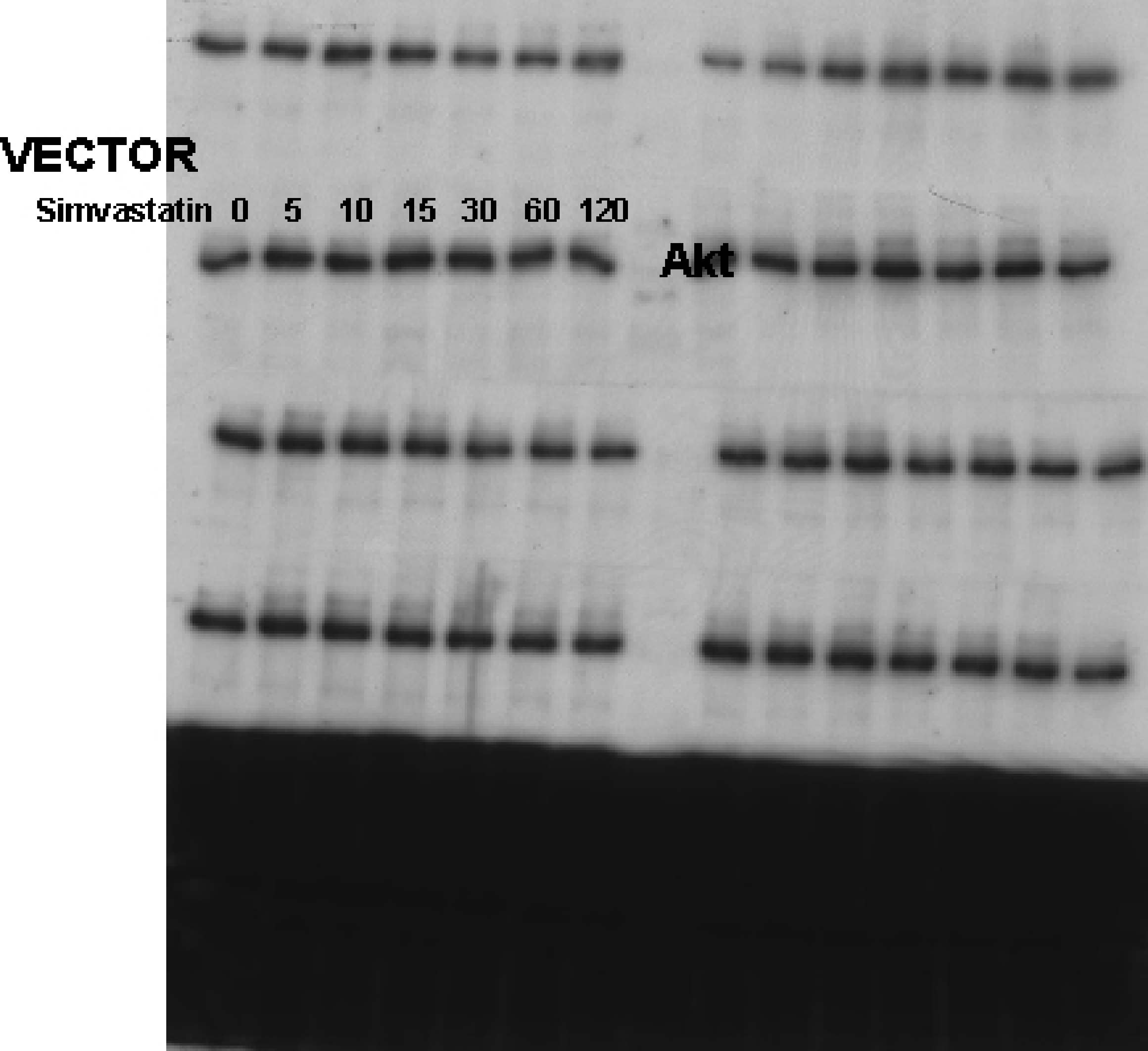
VECTOR
Simvastatin 0 5 10 15 30 60 120
Akt

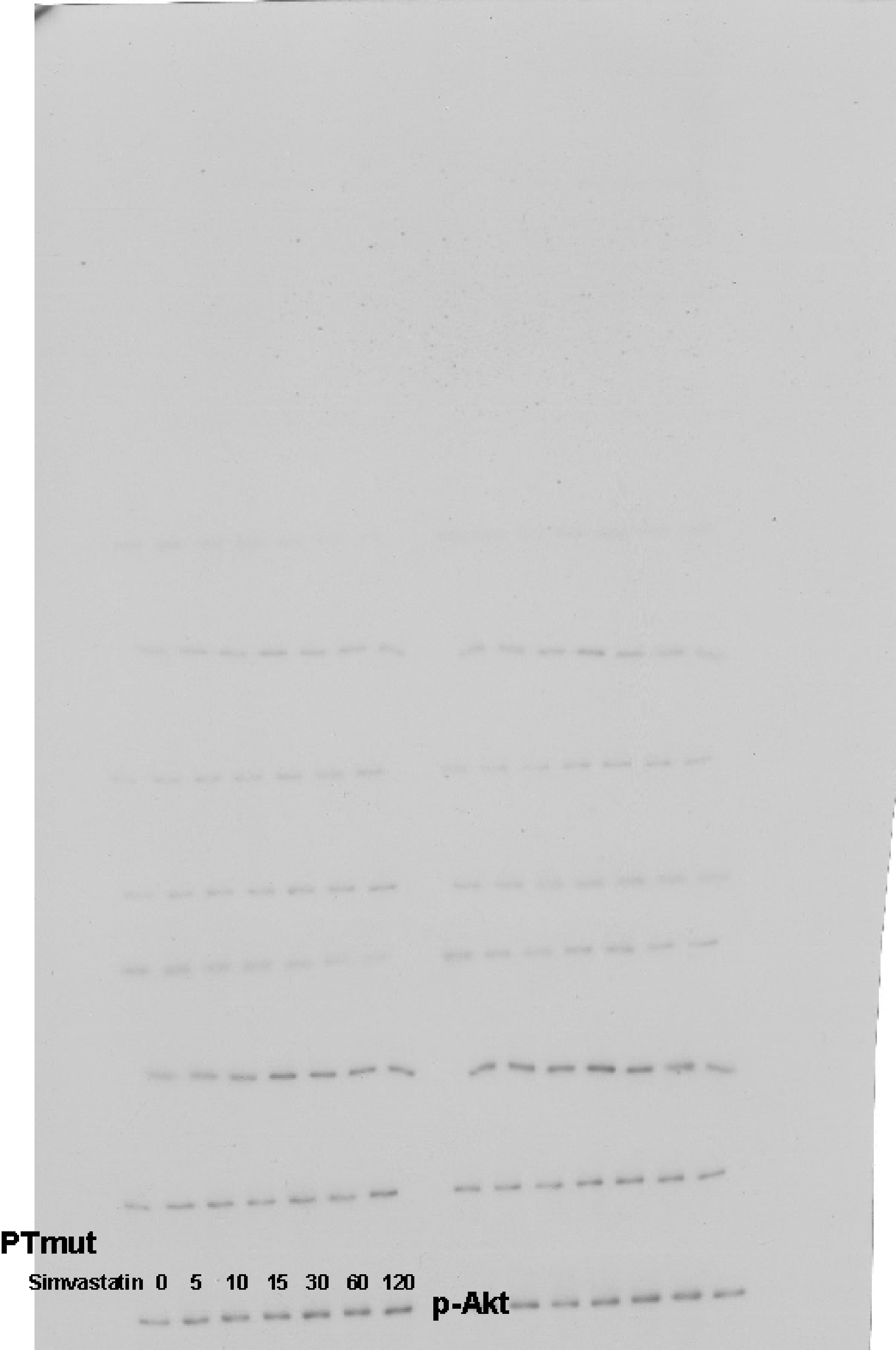
PTmut
Simvastatin 0 5 10 15 30 60 120
p-Akt

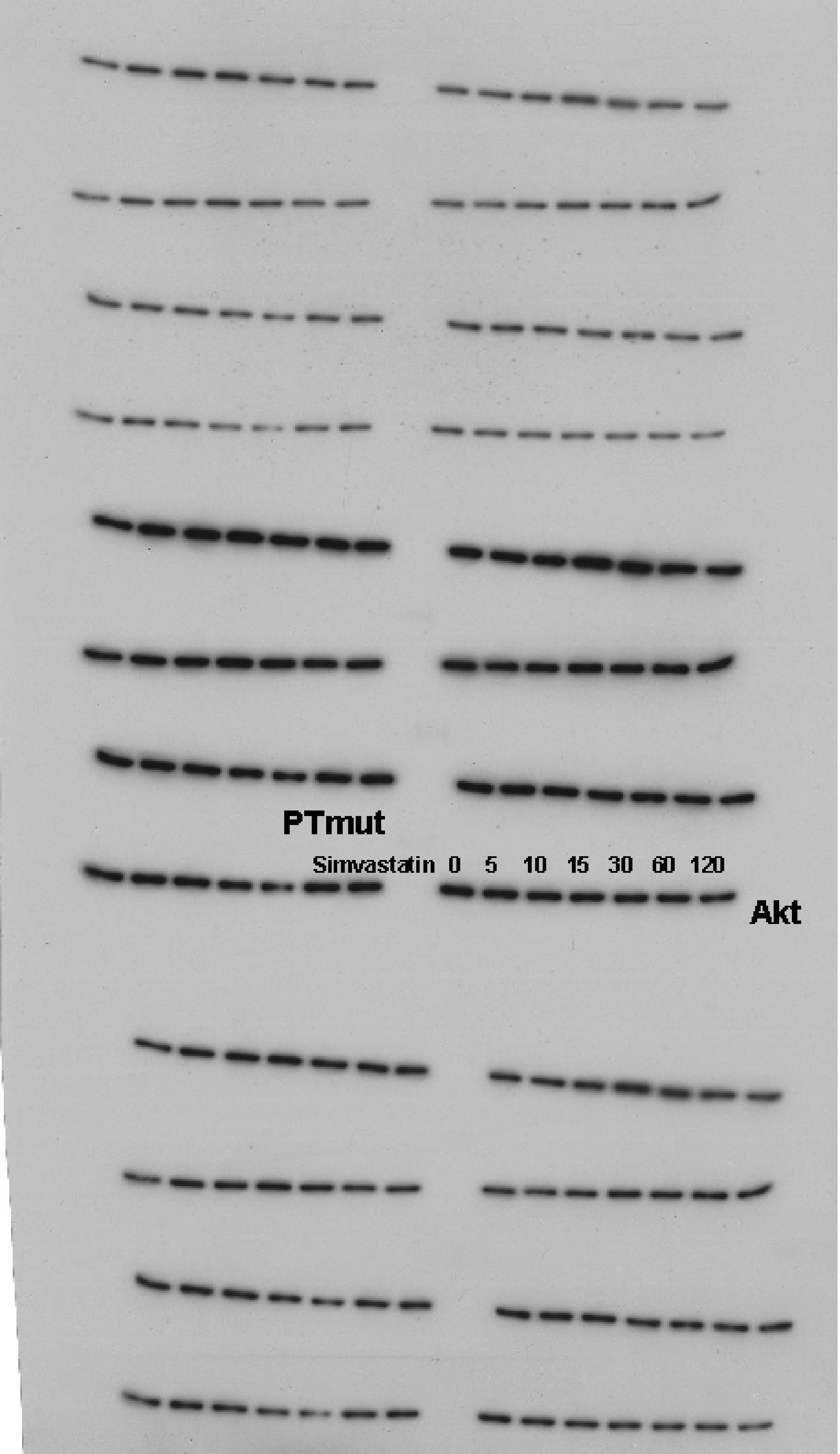
PTmut
Simvastatin 0 5 10 15 30 60 120
Akt

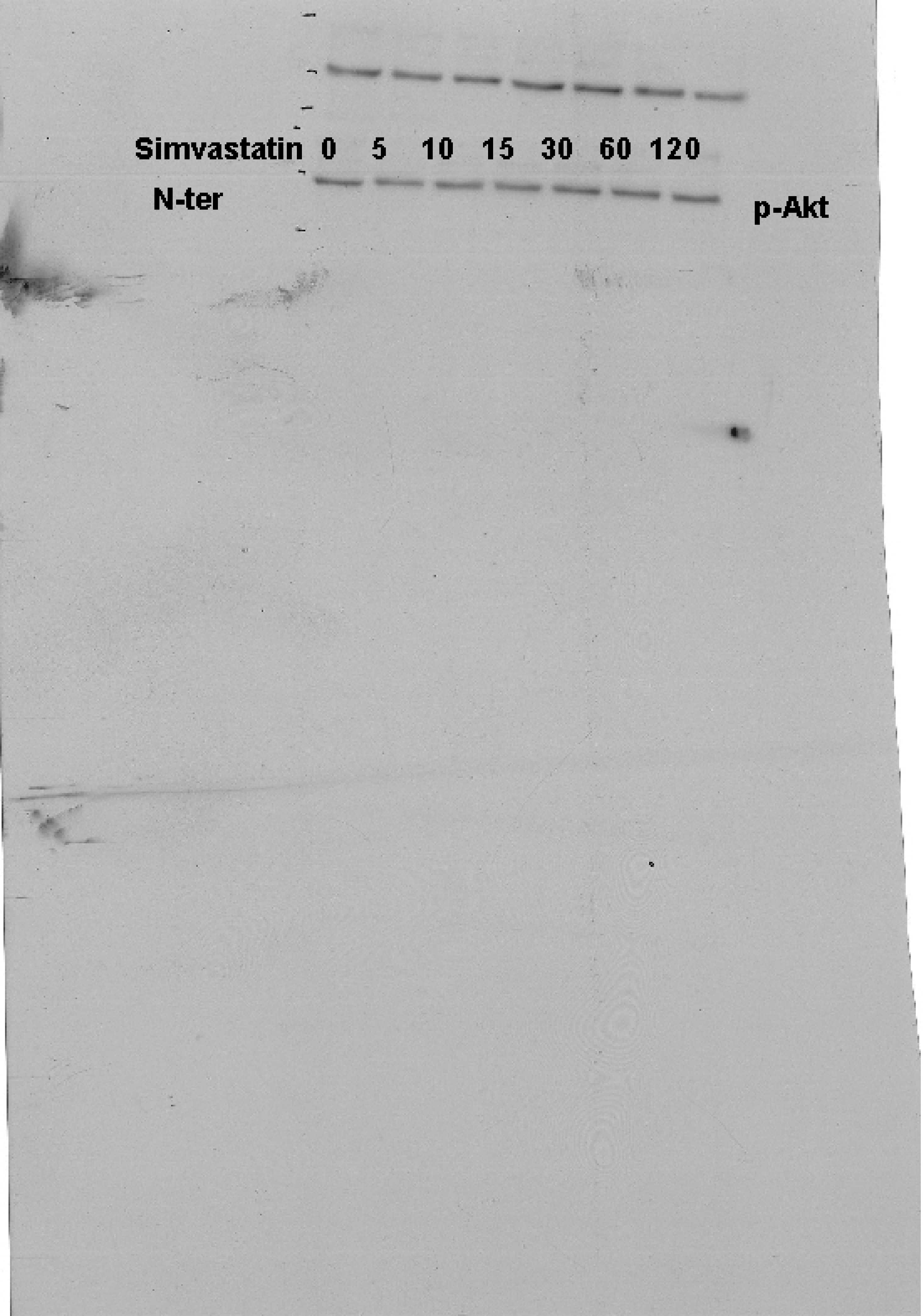
Simvastatin 0 5 10 15 30 60 120
N-ter
p-Akt

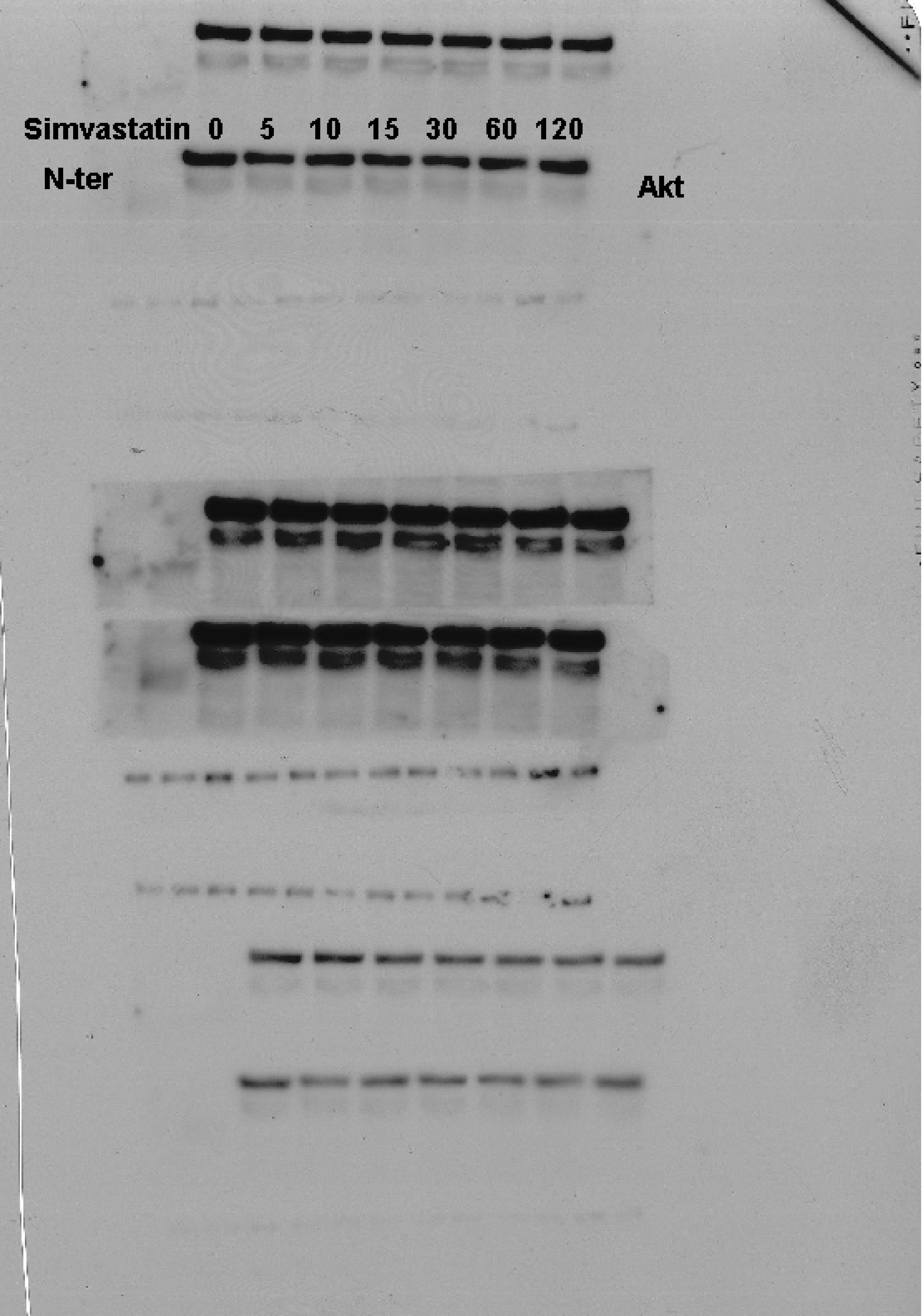
Simvastatin 0 5 10 15 30 60 120
N-ter
Akt

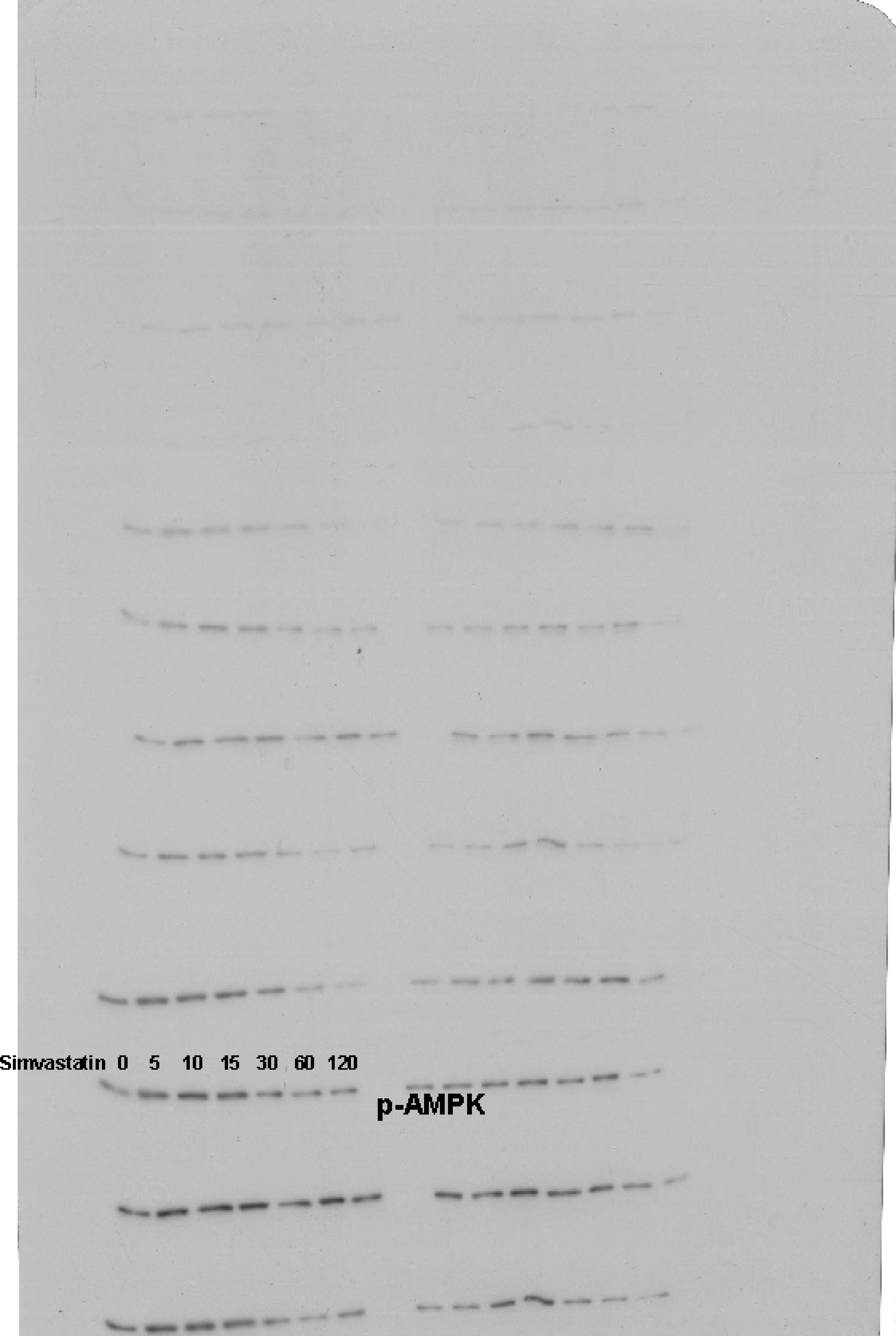
Simvastatin 0 5 10 15 30 60 120
p-AMPK

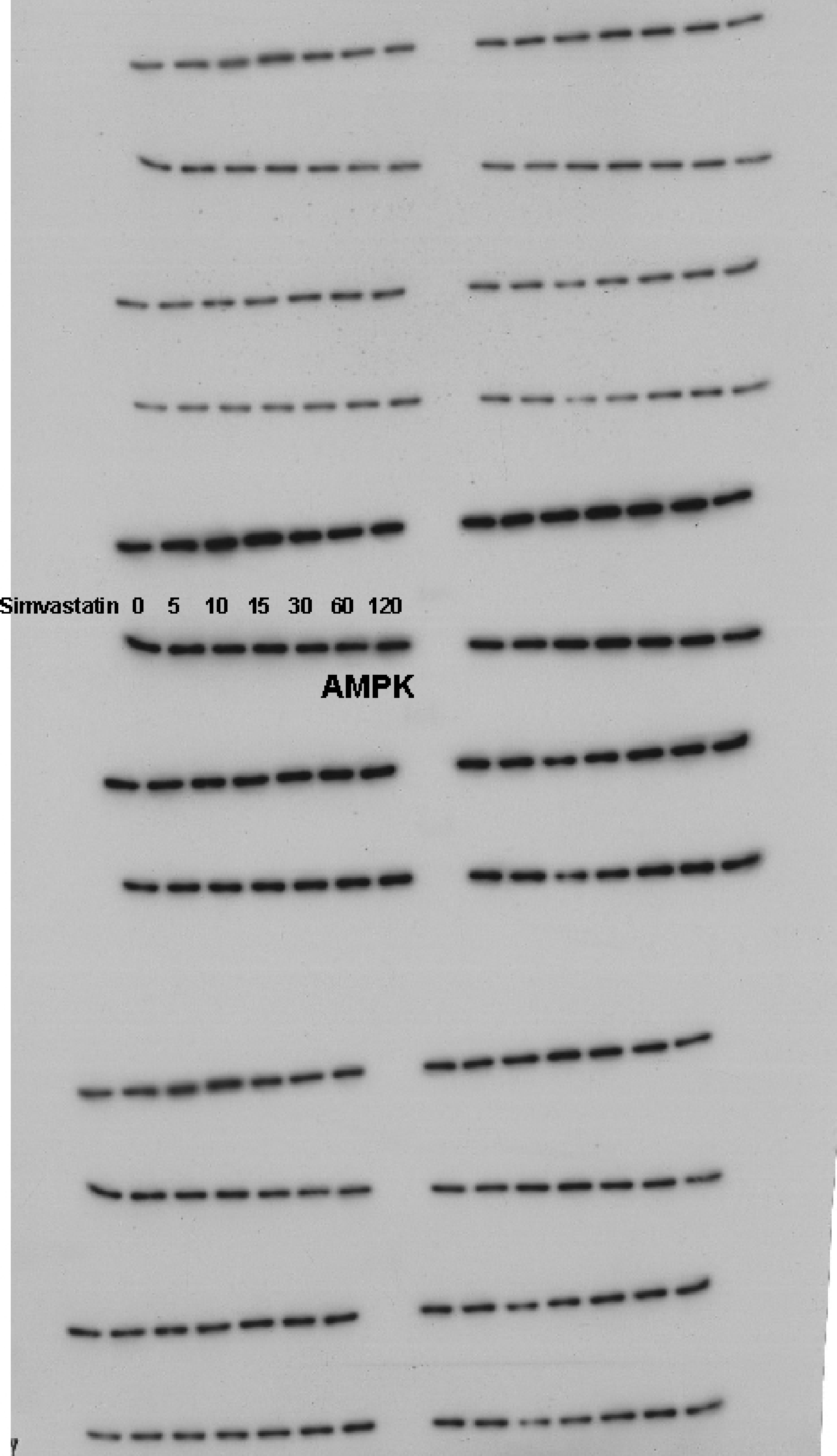
Simvastatin 0 5 10 15 30 60 120
AMPK

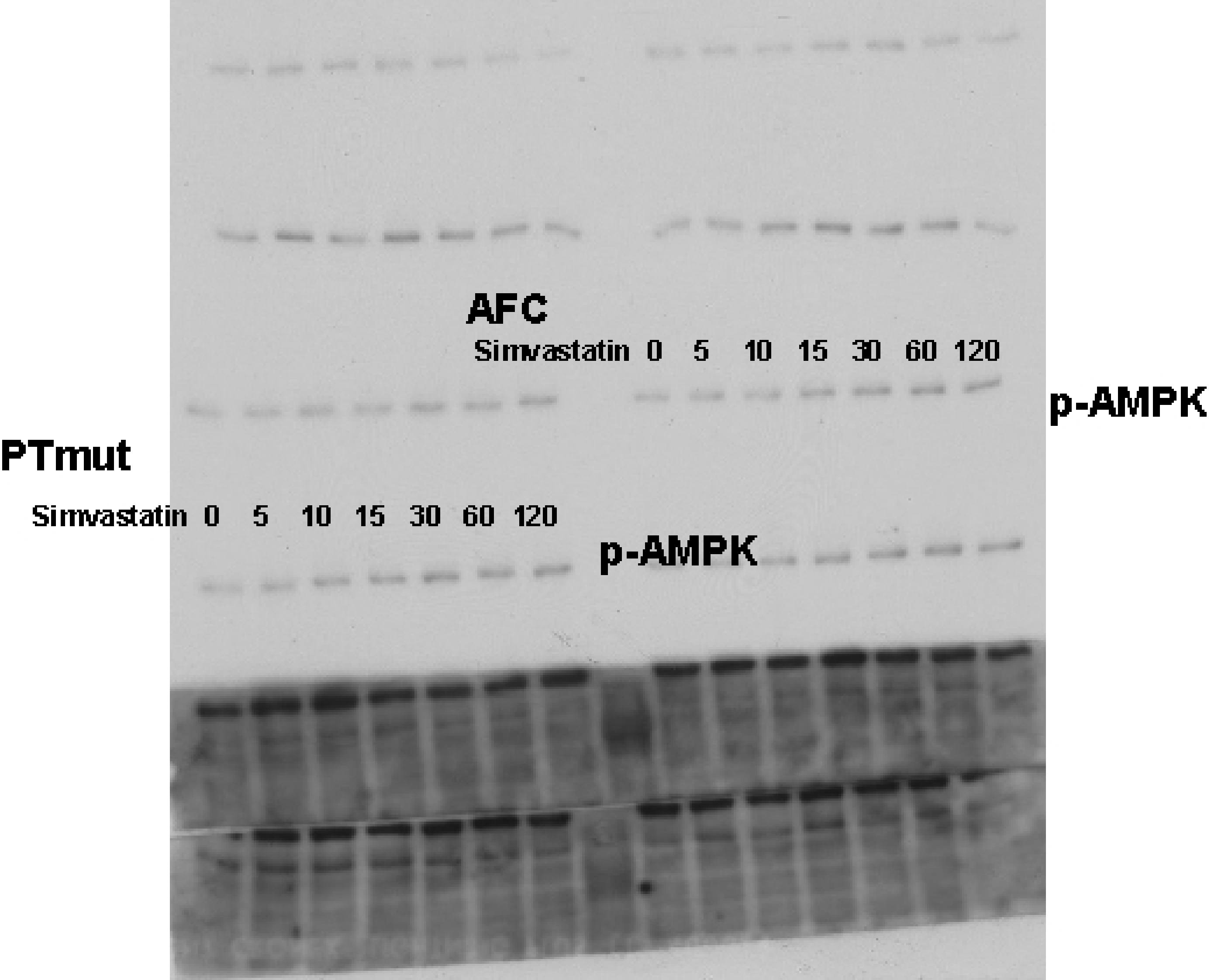
AFC
Simvastatin 0 5 10 15 30 60 120
p-AMPK
PTmut
Simvastatin 0 5 10 15 30 60 120
p-AMPK

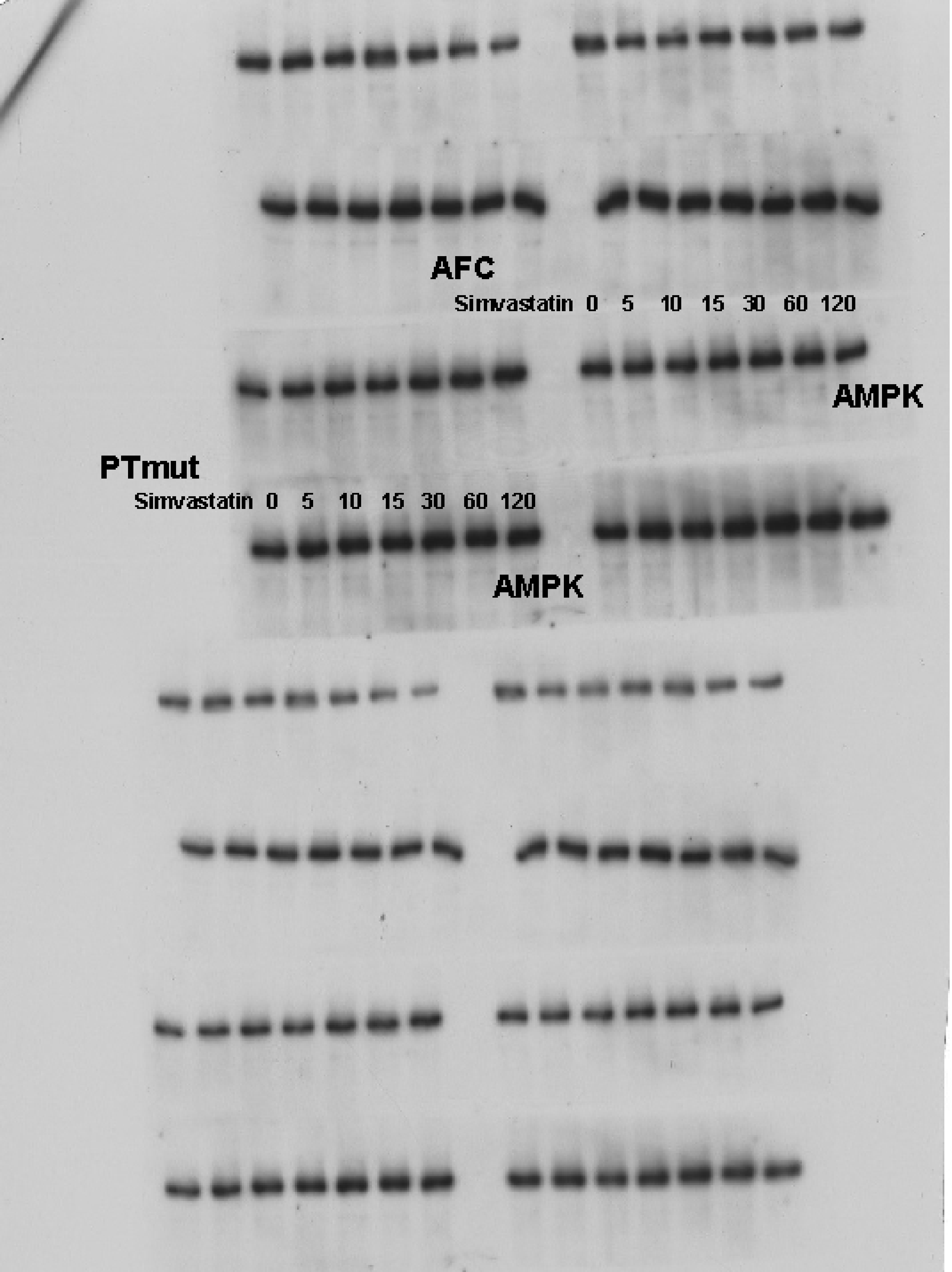
AFC
Simvastatin 0 5 10 15 30 60 120
AMPK
PTmut
Simvastatin 0 5 10 15 30 60 120
AMPK

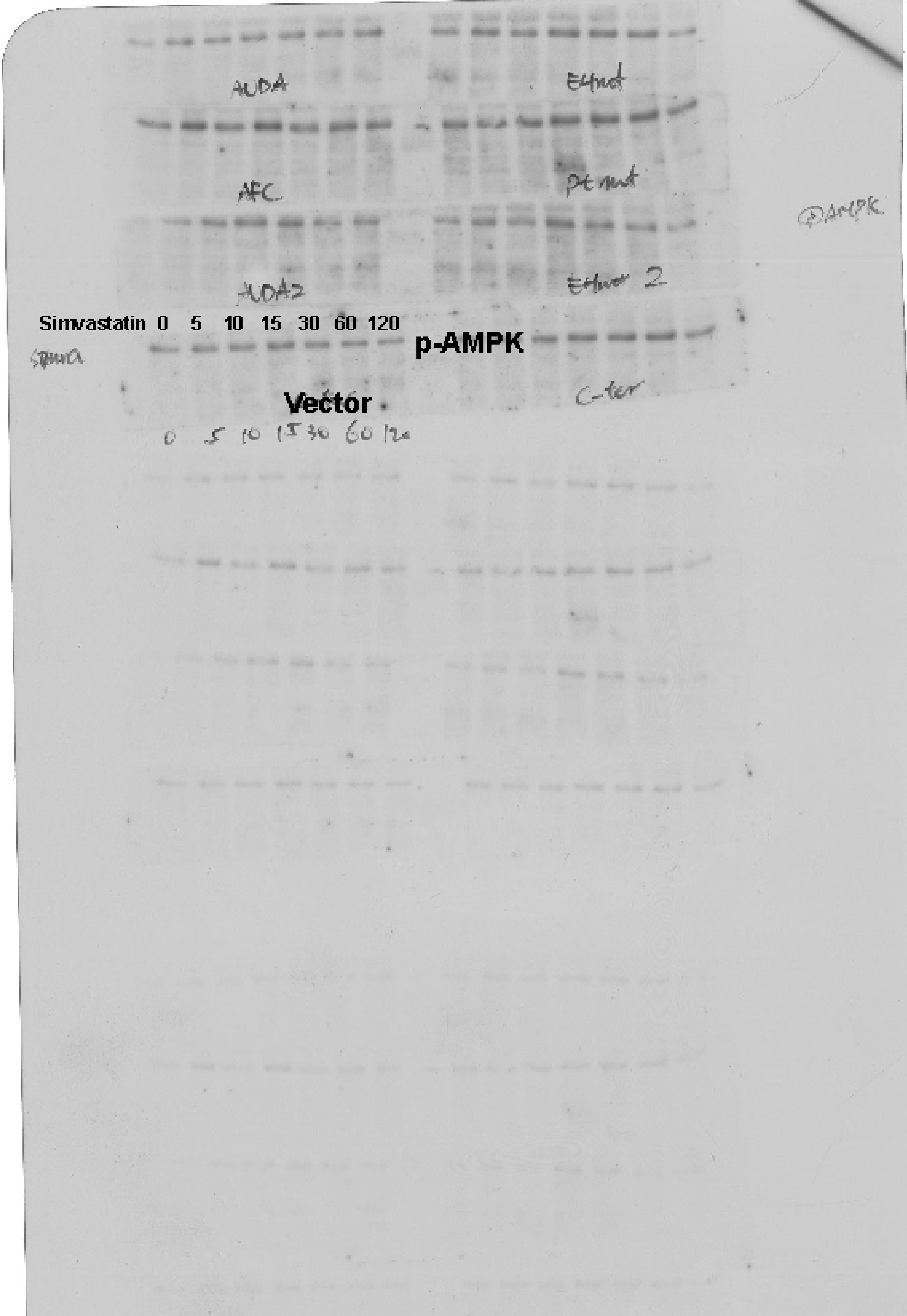
AUDA
AFC
AUDA2
Simvastatin 0 5 10 15 30 60 120
p-AMPK
C-ter
Vector
0 5 10 15 30 60 120

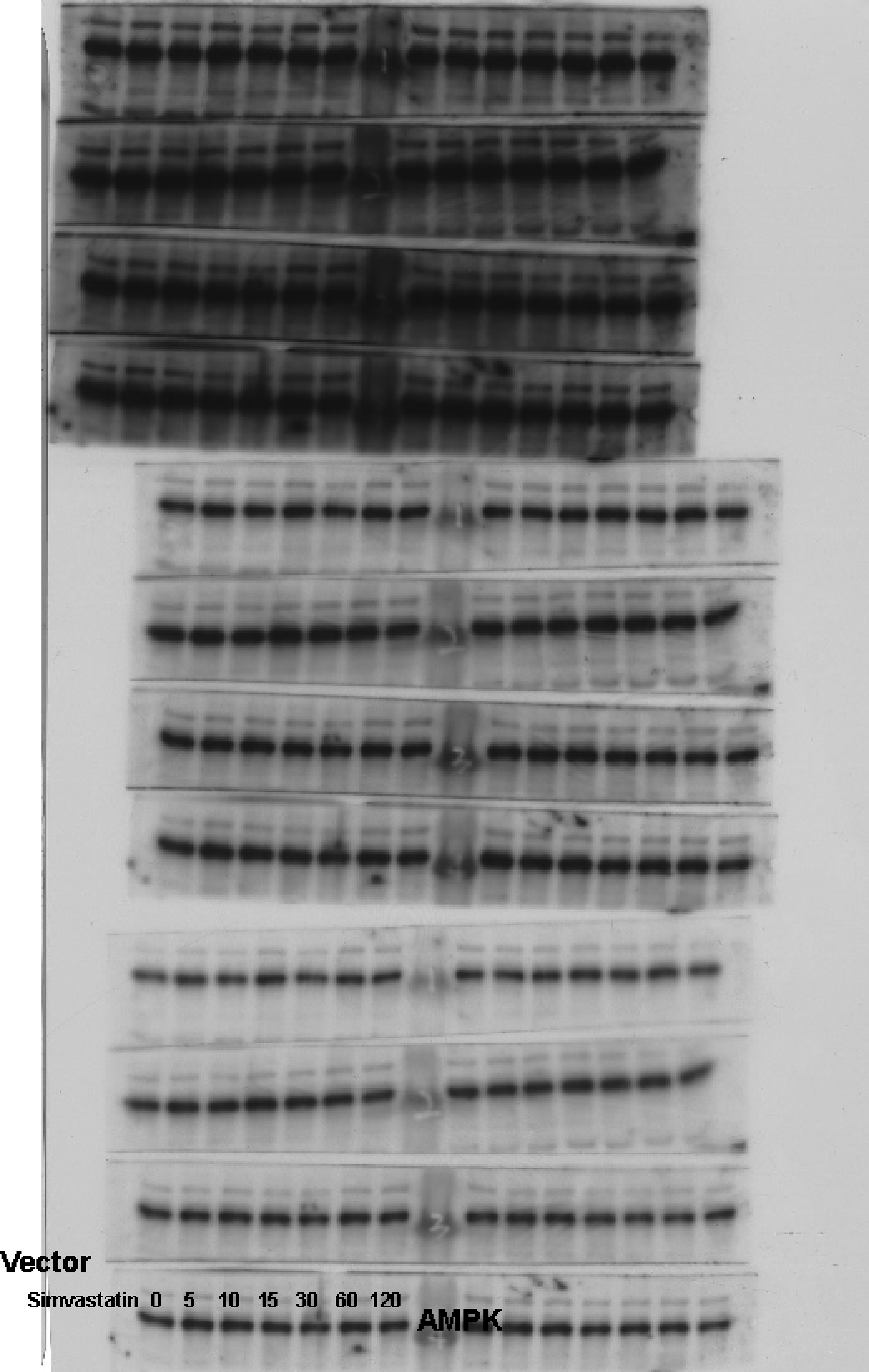
Vector
Simvastatin 0 5 10 15 30 60 120
AMPK

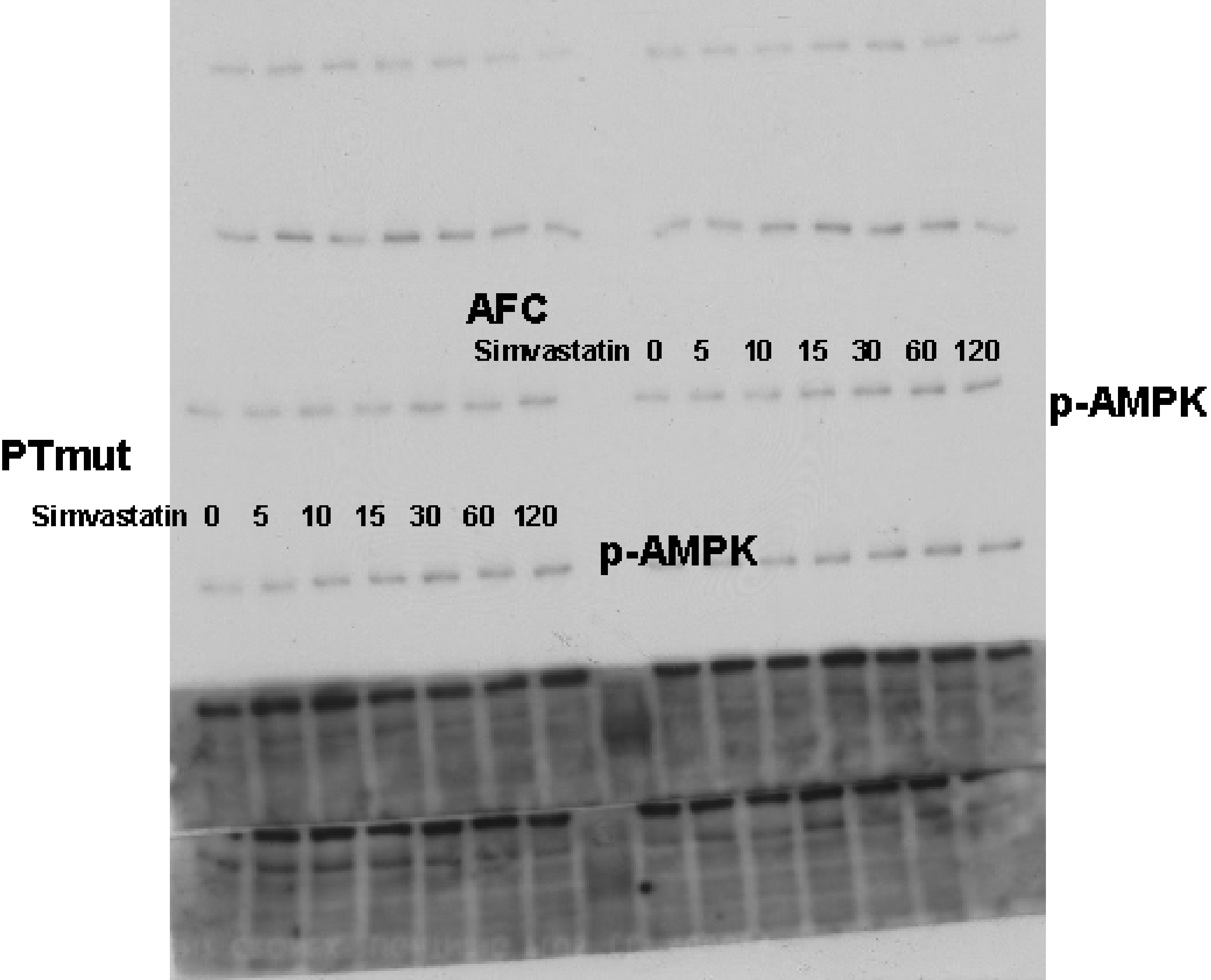
AFC
Simvastatin 0 5 10 15 30 60 120
p-AMPK
PTmut
Simvastatin 0 5 10 15 30 60 120
p-AMPK

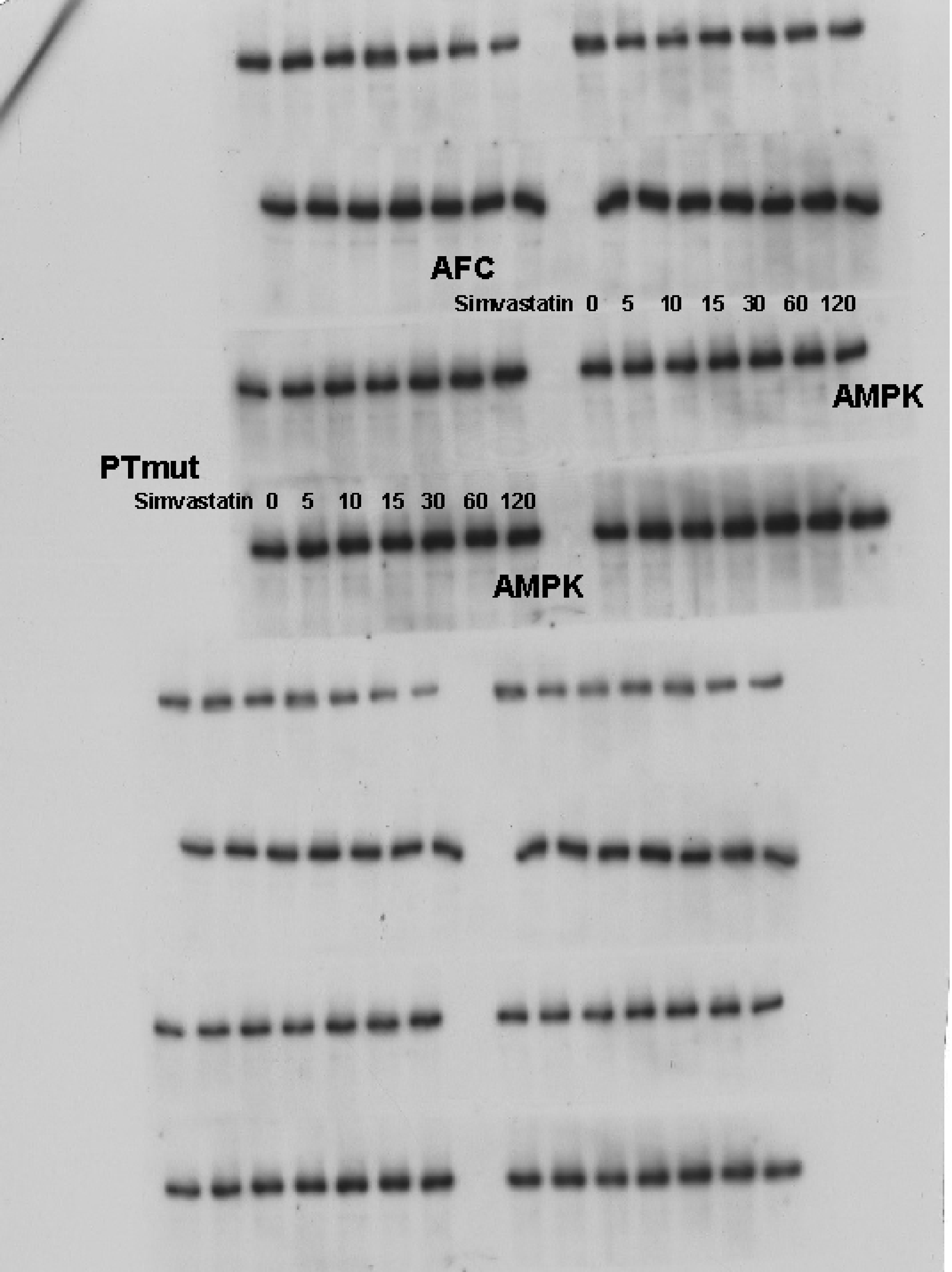
AFC
Simvastatin 0 5 10 15 30 60 120
AMPK
PTmut
Simvastatin 0 5 10 15 30 60 120
AMPK

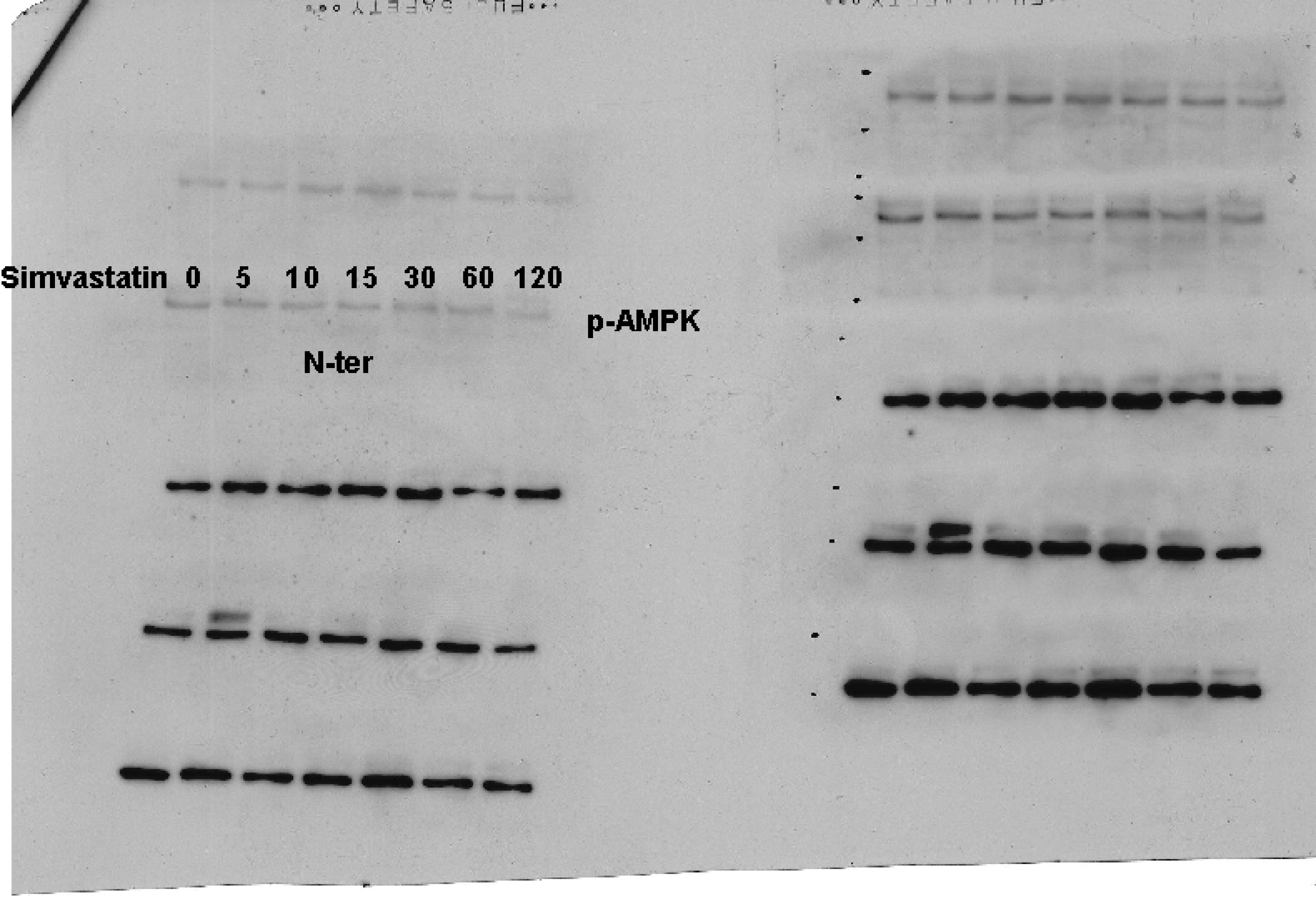
Simvastatin 0 5 10 15 30 60 120
p-AMPK
N-ter

Fig S5. **Simvastatin promotes the formation of an sEH–eNOS–Akt–AMPK complex.**

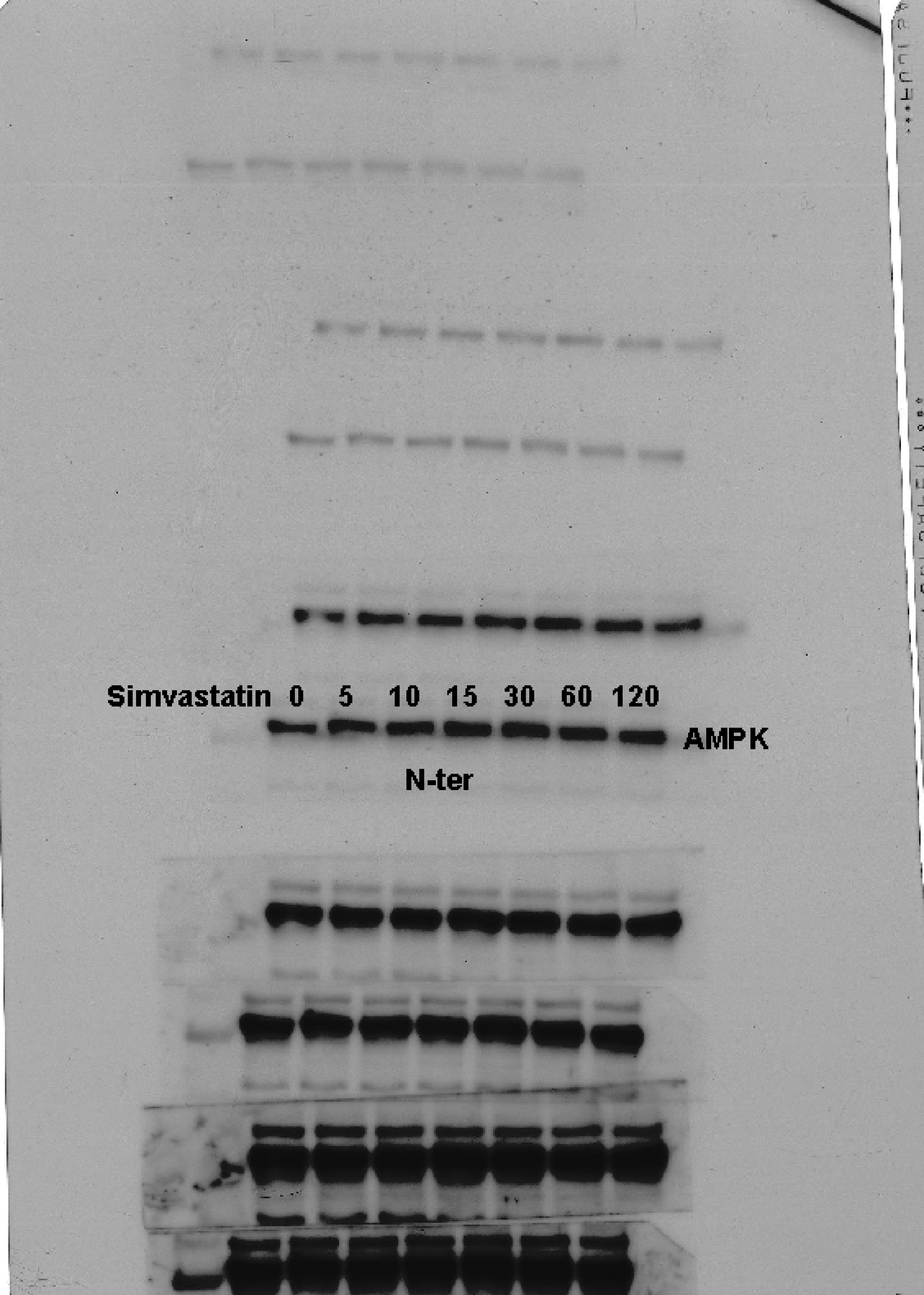
Simvastatin 0 5 10 15 30 60 120
AMPK
N-ter

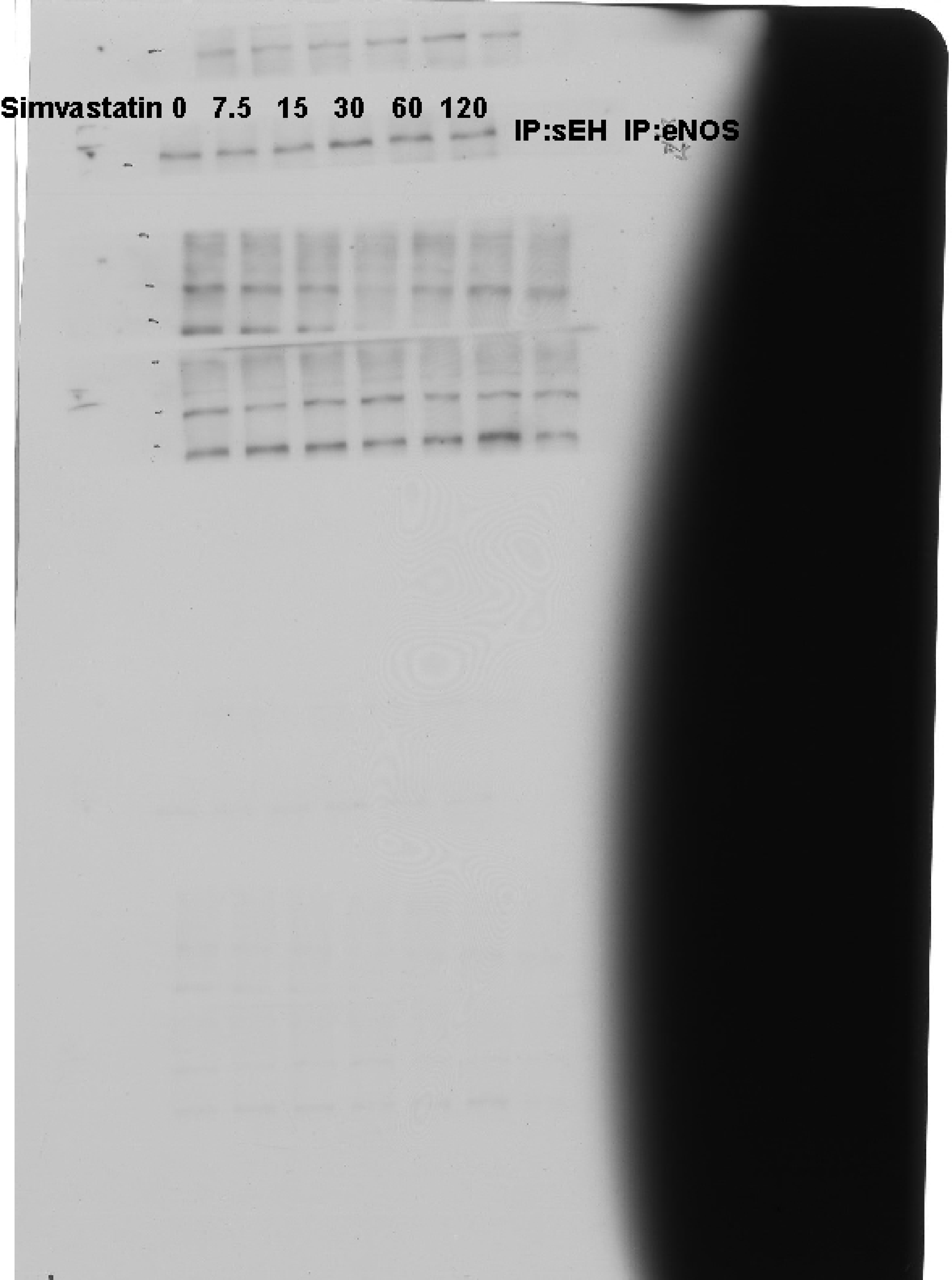
Simvastatin 0 7.5 15 30 60 120
IP:sEH IP:eNOS

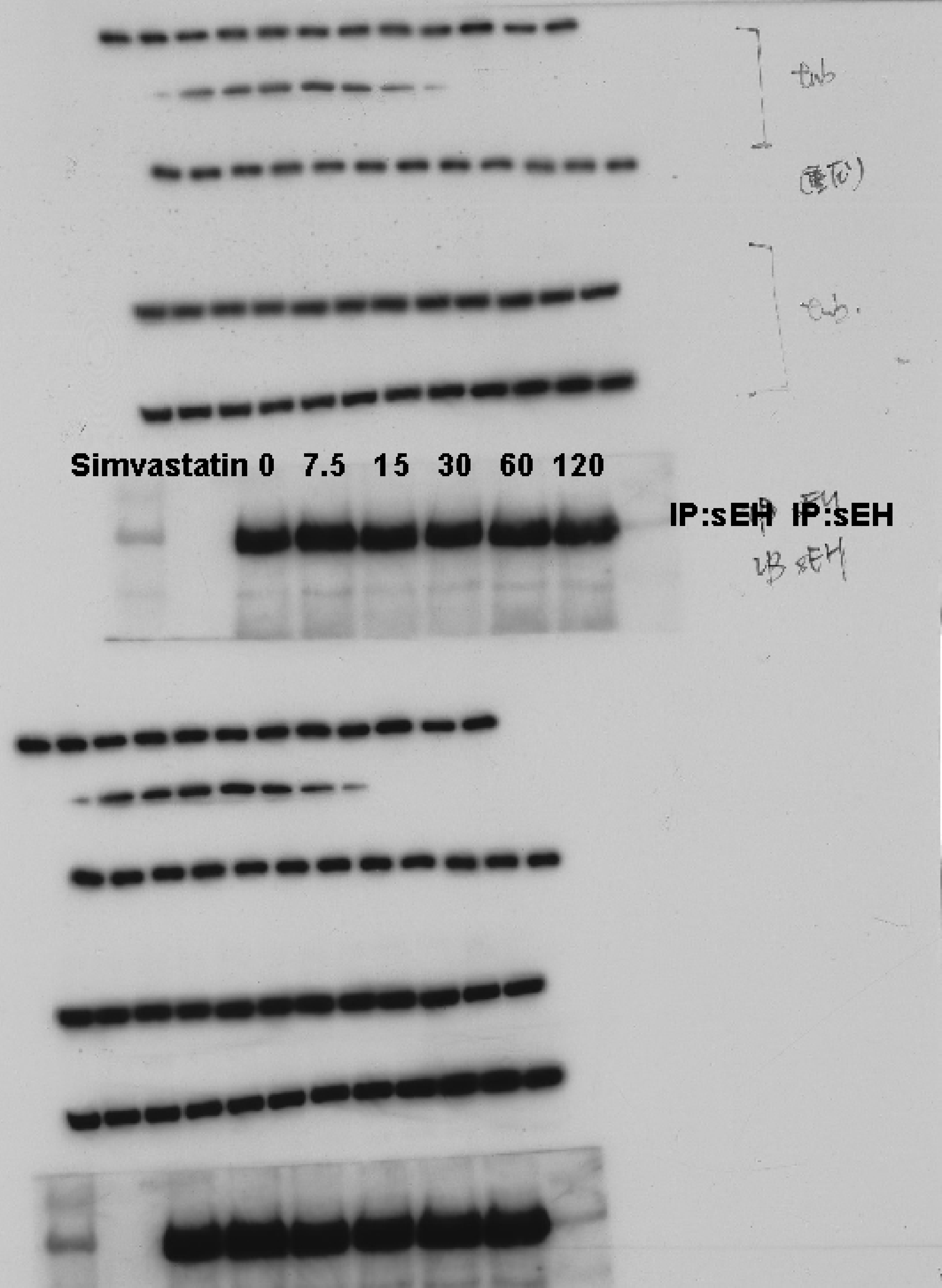
Simvastatin 0 7.5 15 30 60 120
IP:sEH IP:sEH

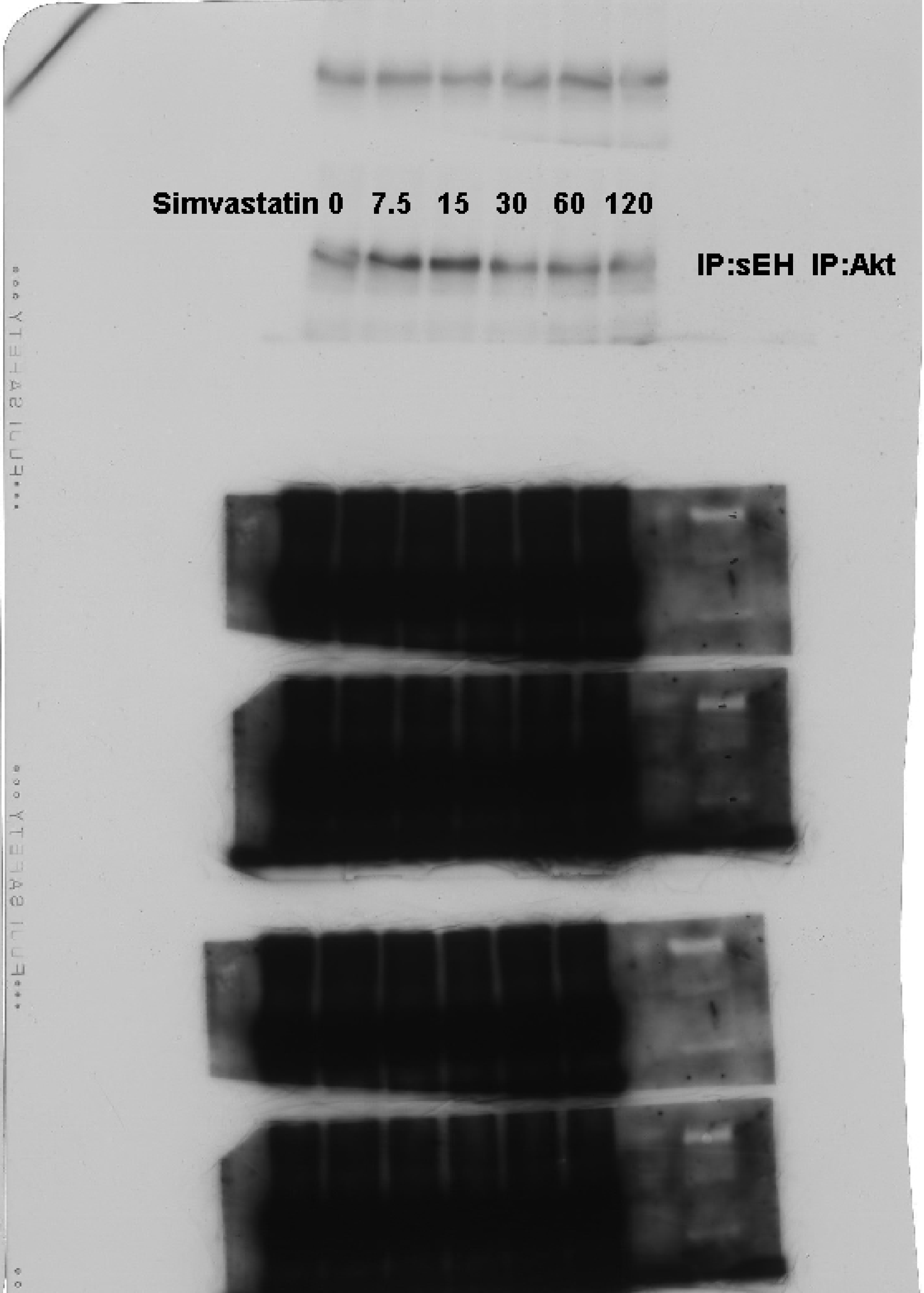
Simvastatin 0 7.5 15 30 60 120
IP:sEH IP:Akt

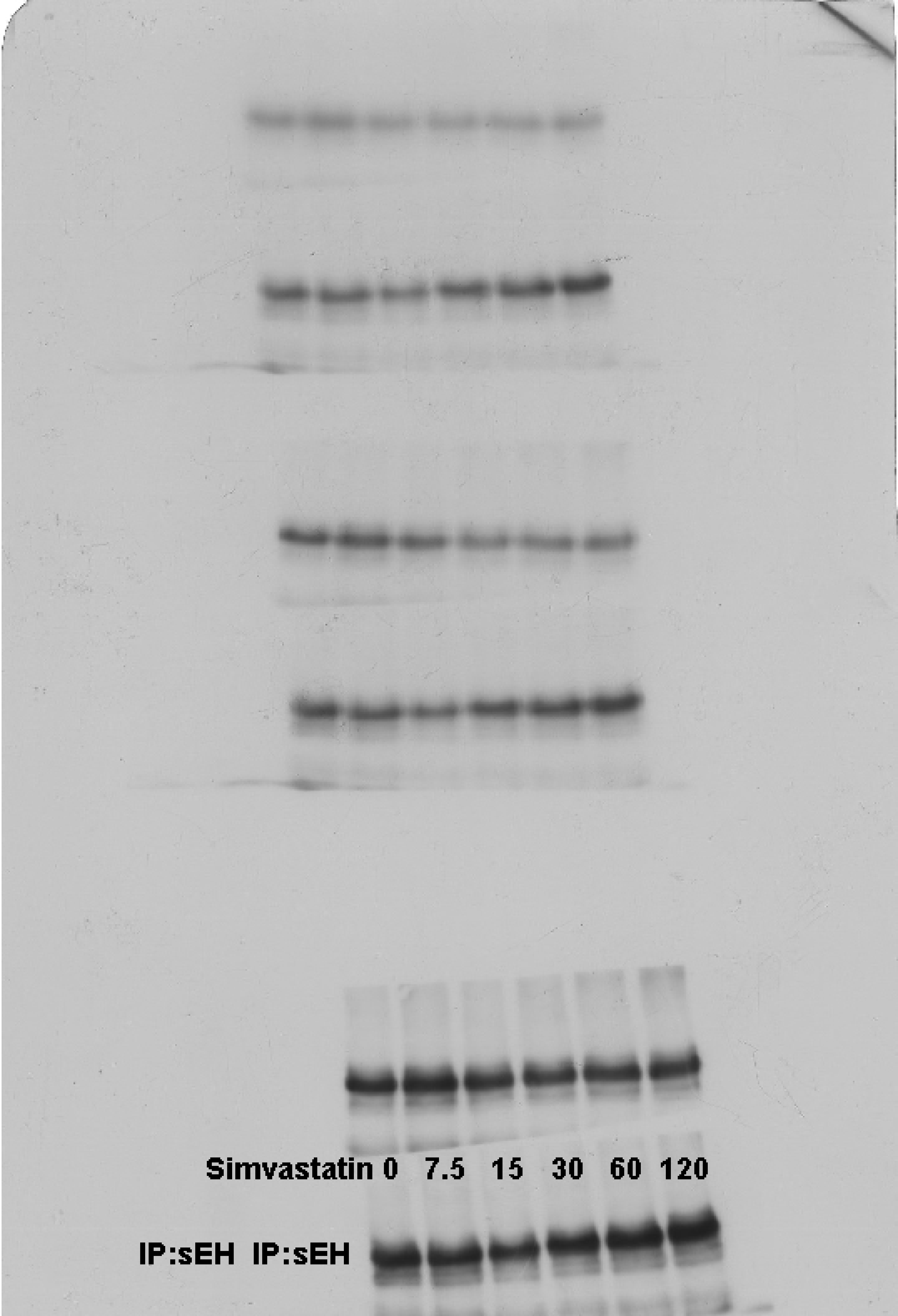
Simvastatin 0 7.5 15 30 60 120
IP:sEH IP:sEH

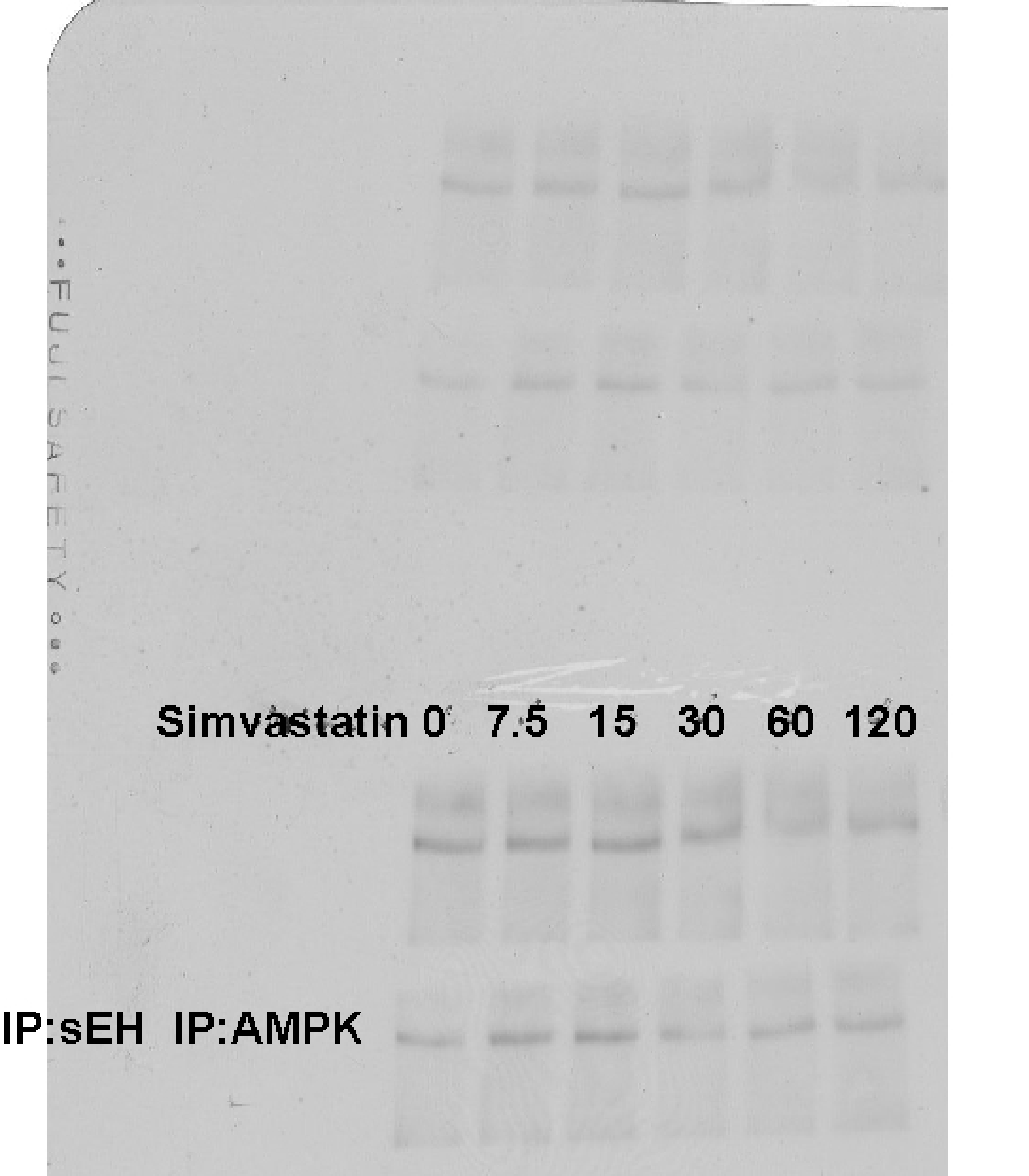

FUJI SAFETY
Simvastatin 0 7.5 15 30 60 120
IP:sEH IP:AMPK

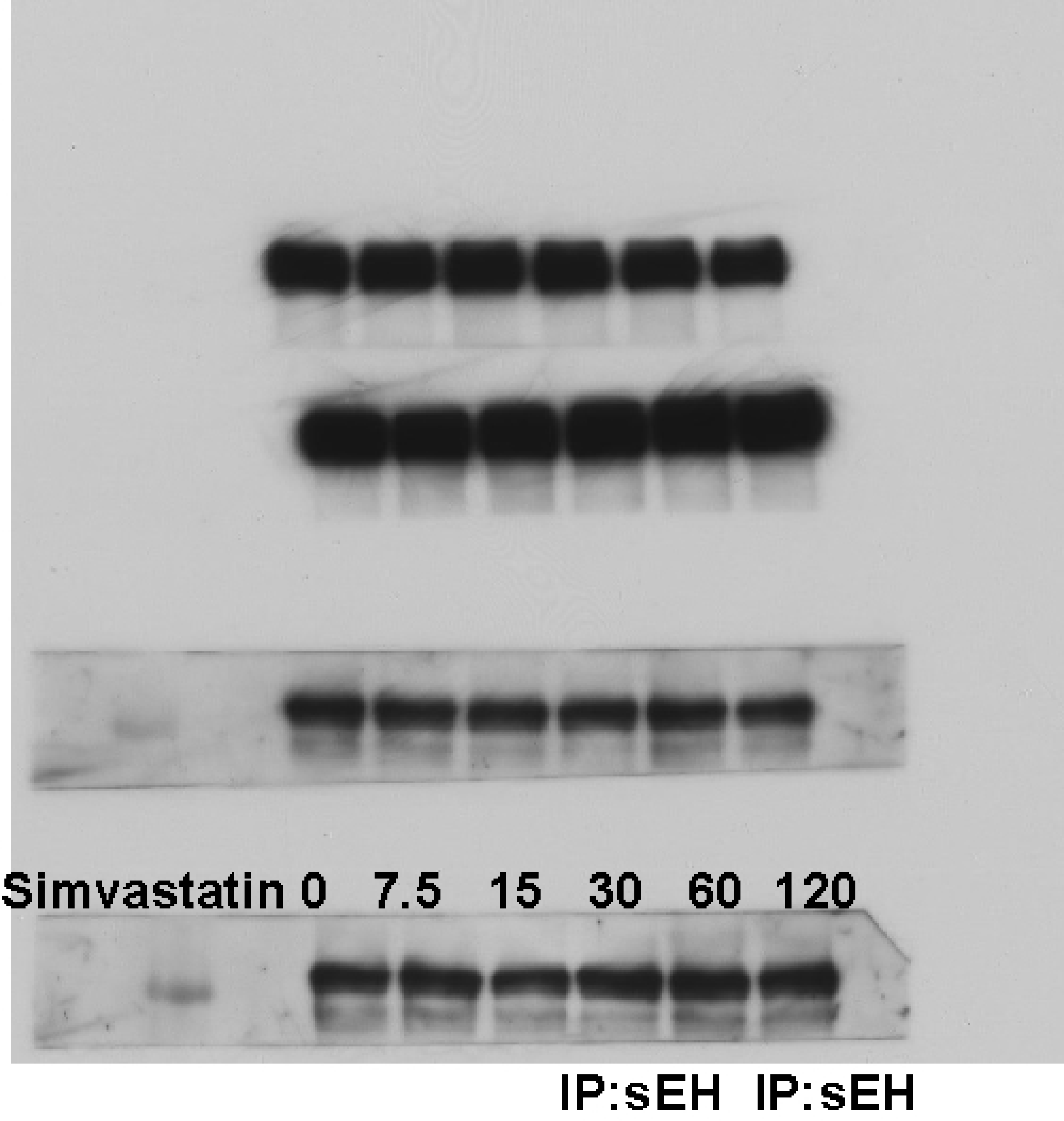
Simvastatin 0 7.5 15 30 60 120
IP:sEH IP:sEH

Fig S6. **Activation of epidermal growth factor receptor (EGFR)–c-Src signaling is required for simvastatin-induced increase in sEH phosphatase activity.**

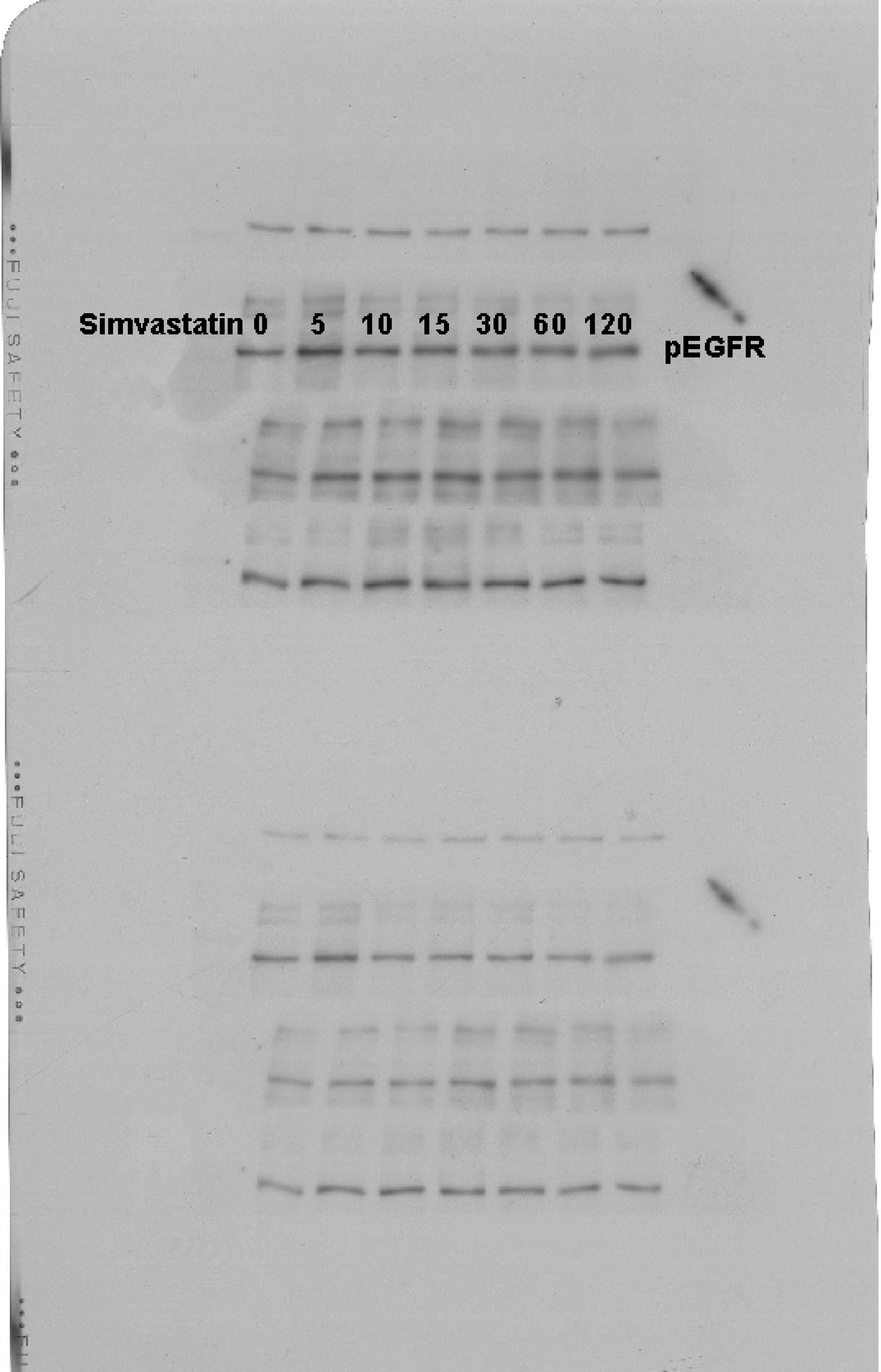
Simvastatin 0 5 10 15 30 60 120
pEGFR

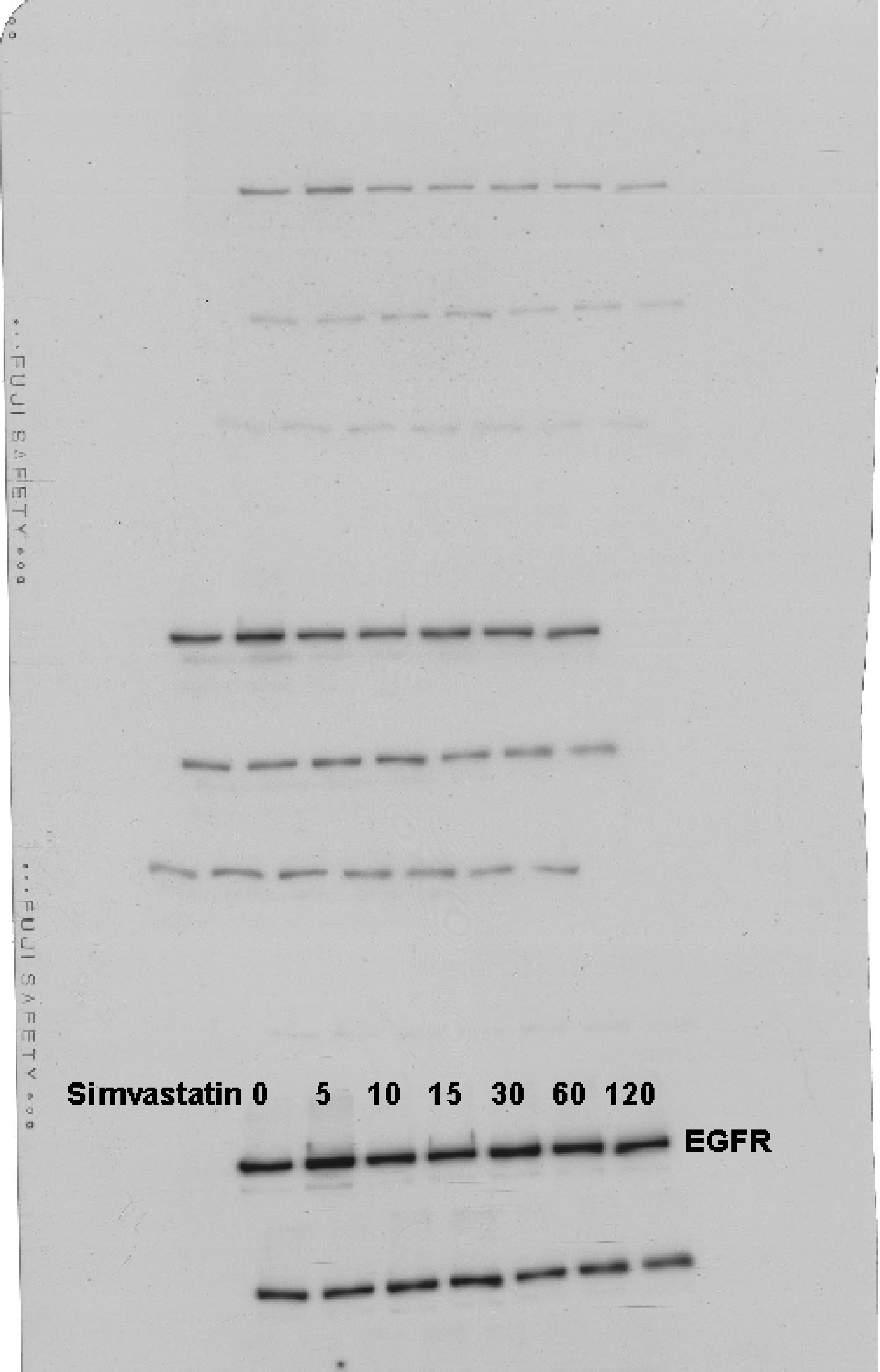
Simvastatin 0 5 10 15 30 60 120
EGFR
FUJI SAFETY
FUJI SAFETY

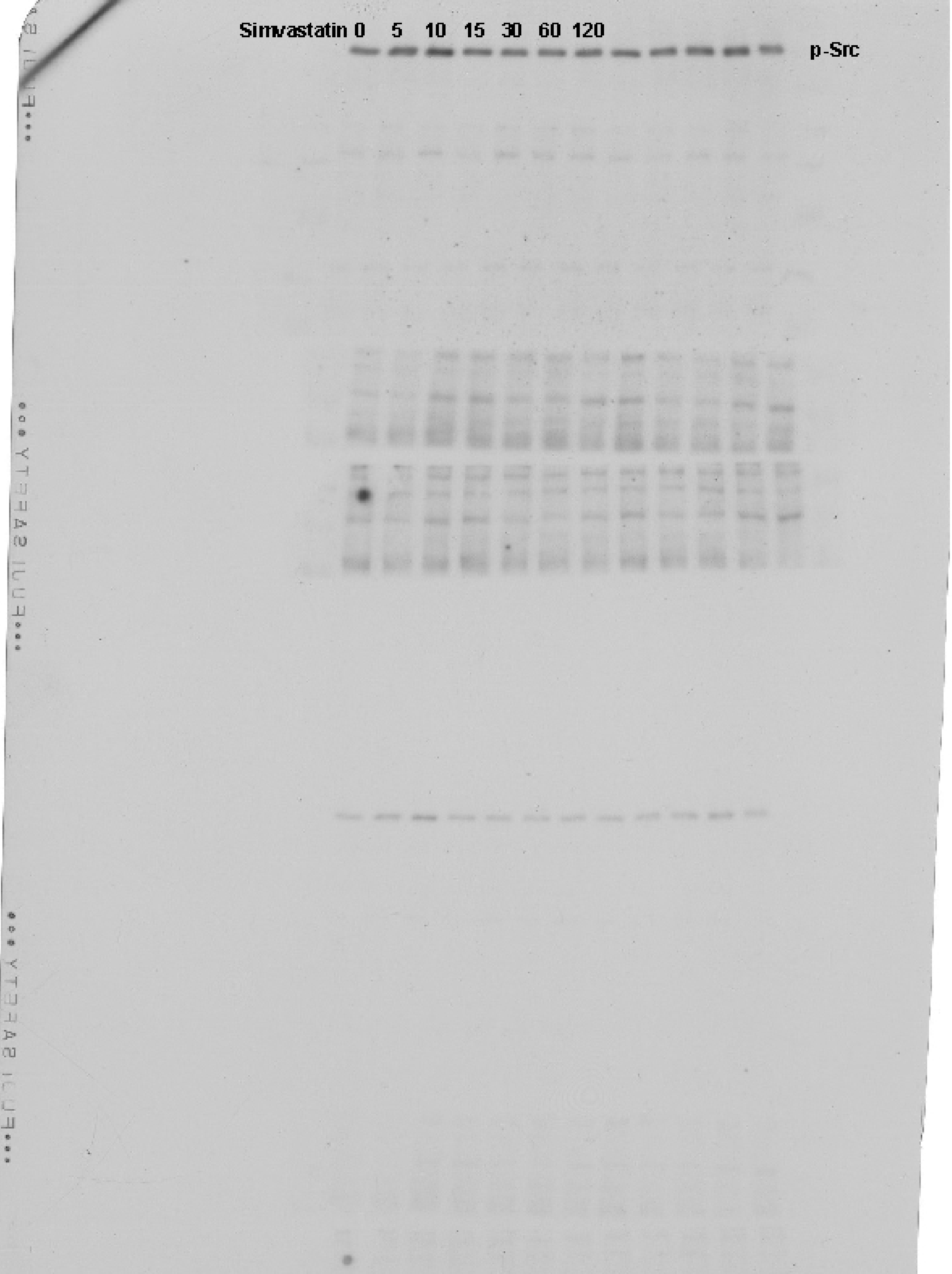
Simvastatin 0 5 10 15 30 60 120
p-Src

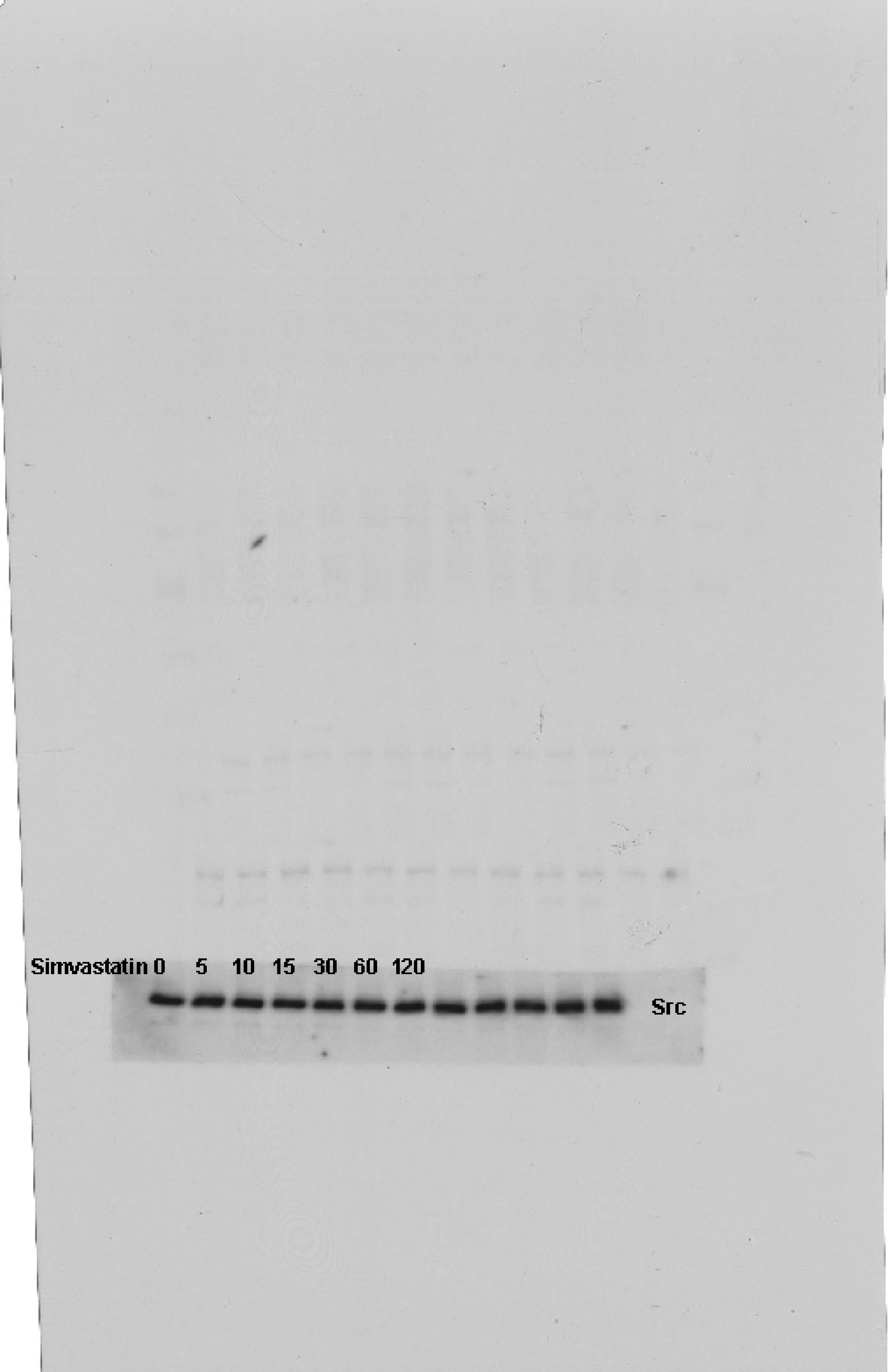
Simvastatin 0 5 10 15 30 60 120
Src

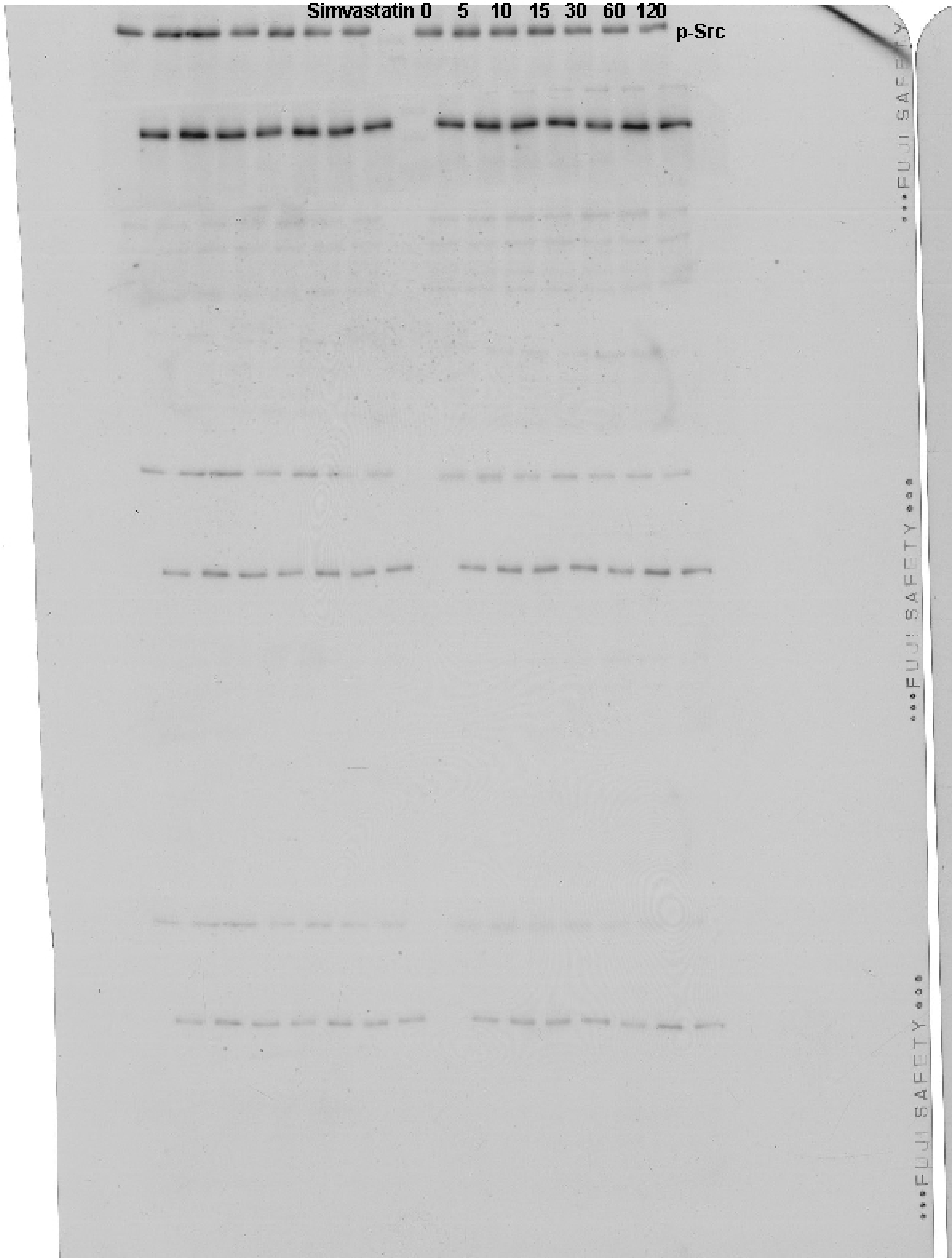
Simvastatin 0 5 10 15 30 60 120
p-Src
FUJI SAFETY
FUJI SAFETY
FUJI SAFETY
FUJI SAFETY

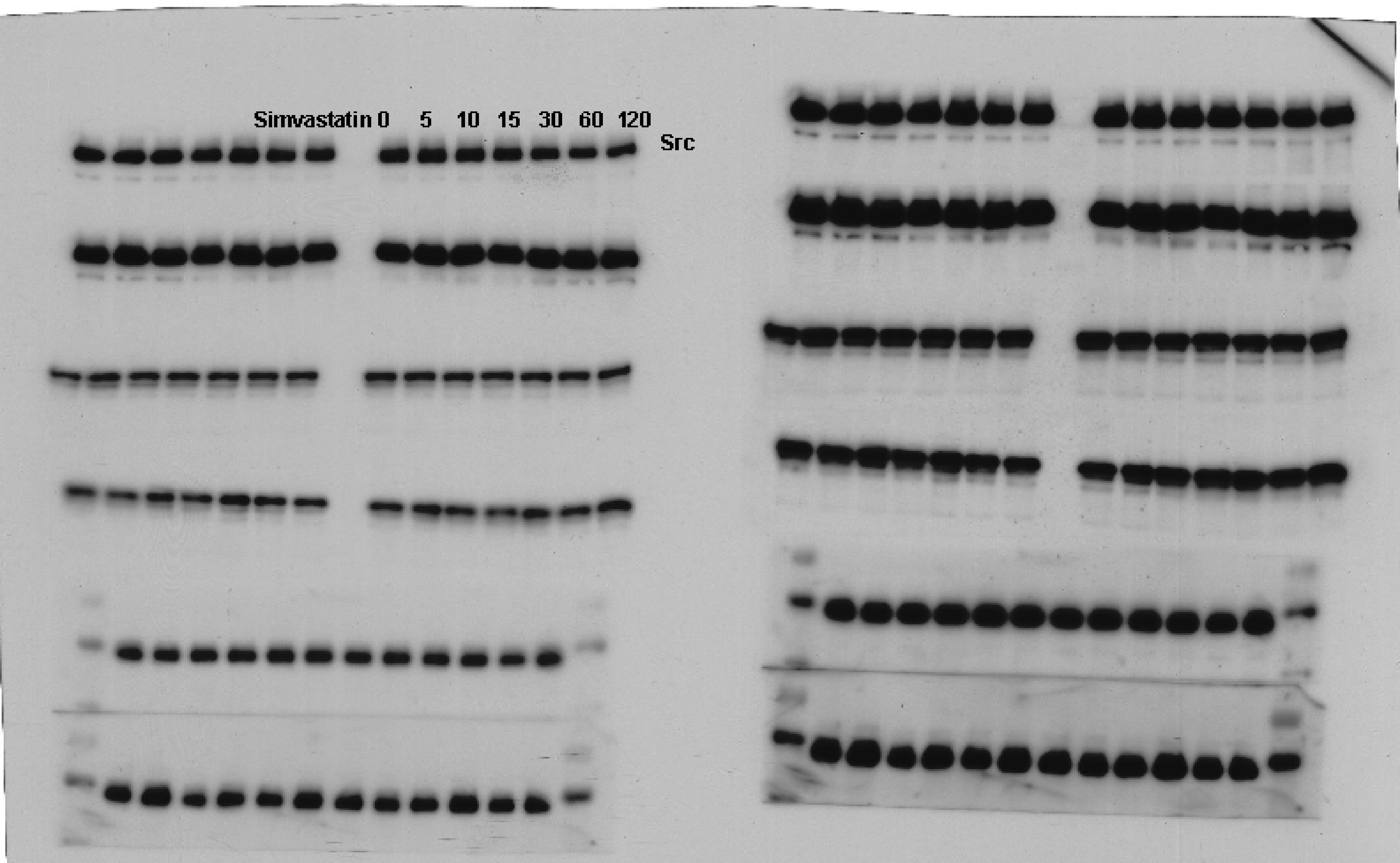
Simvastatin 0 5 10 15 30 60 120
Src

Fig S7. **Simvastatin induces tyrosine phosphorylation of sEH.**

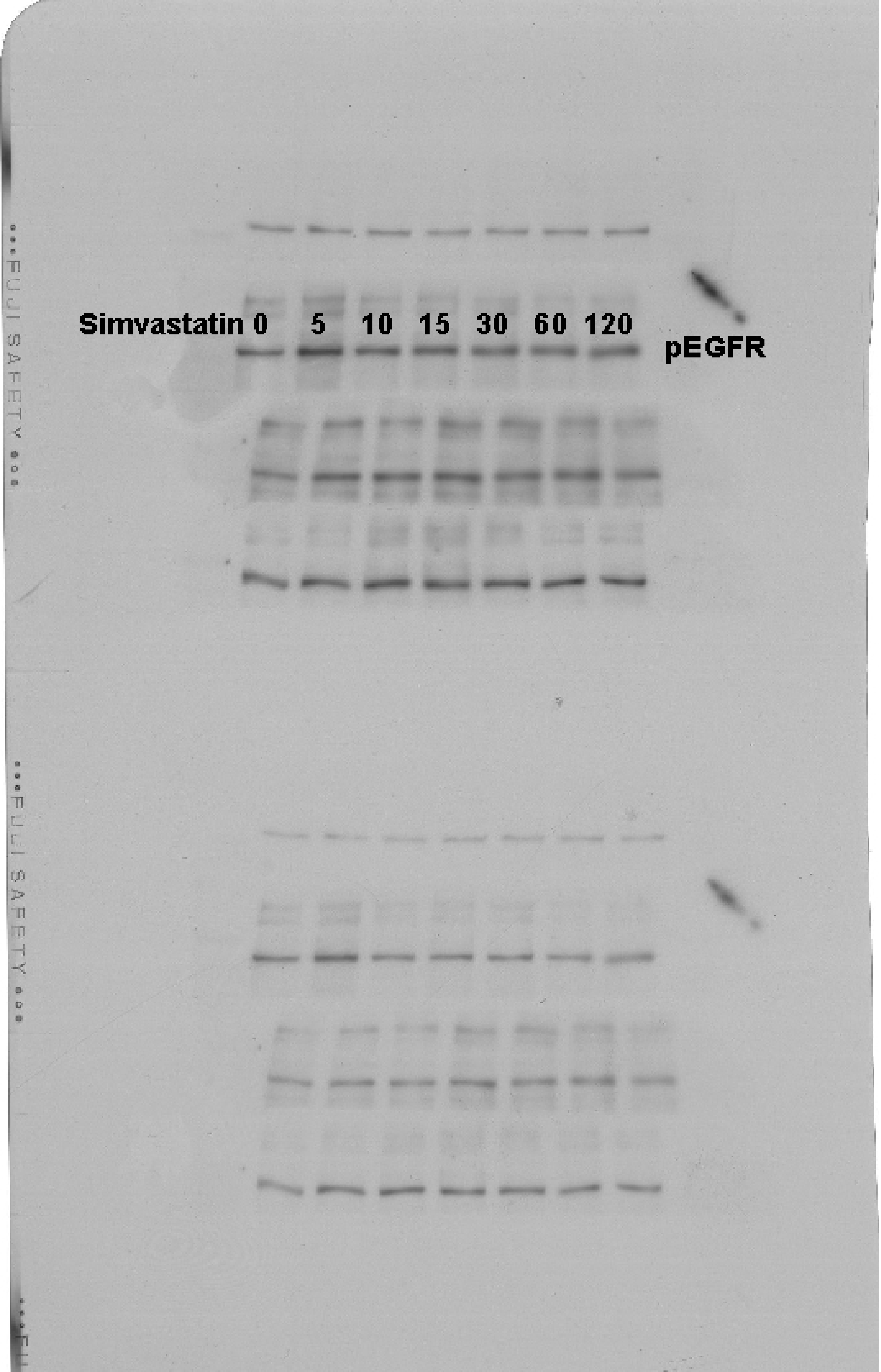
Simvastatin 0 5 10 15 30 60 120
pEGFR
FUJI SAFETY
FUJI SAFETY

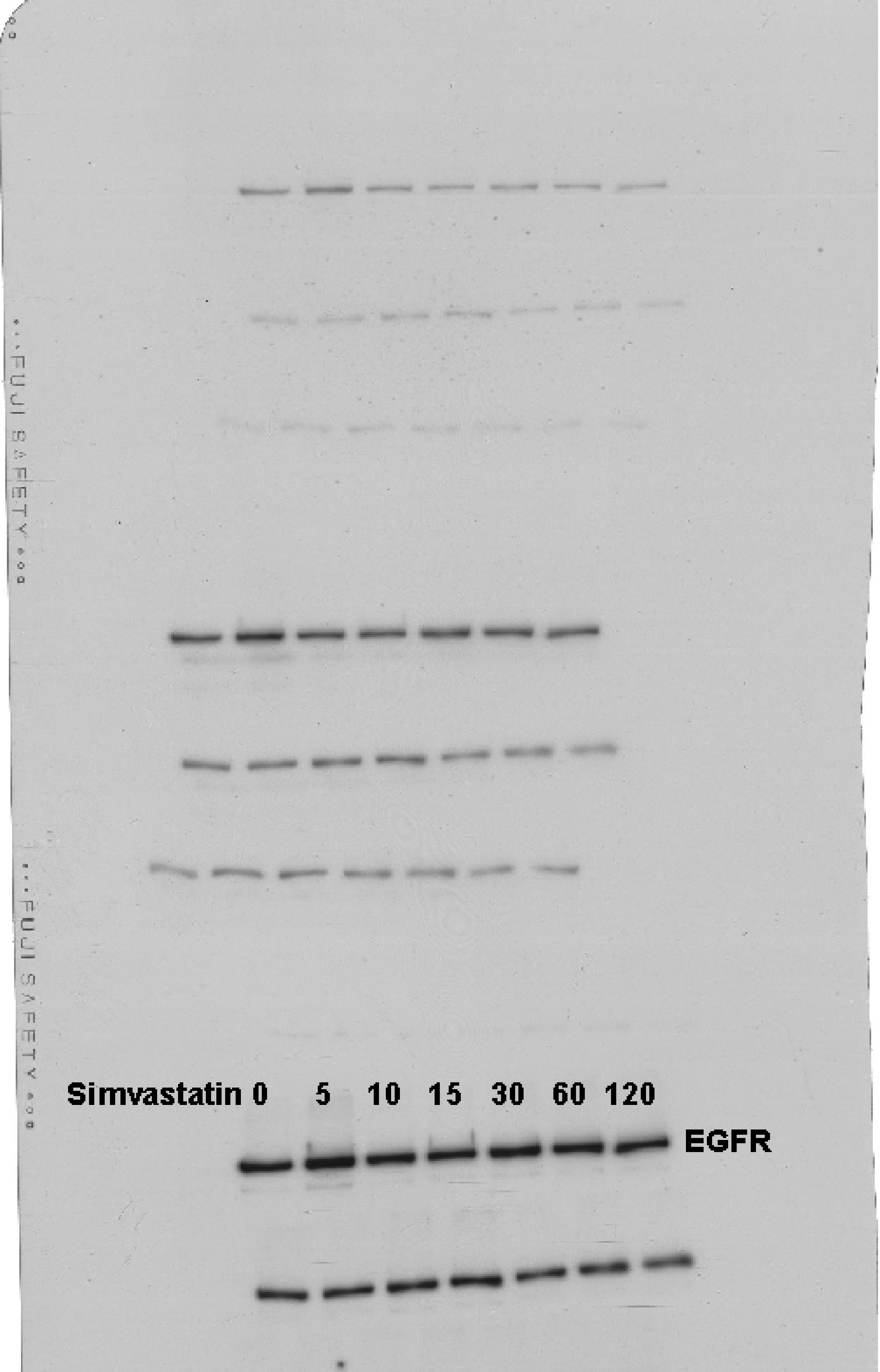
Simvastatin 0 5 10 15 30 60 120
EGFR

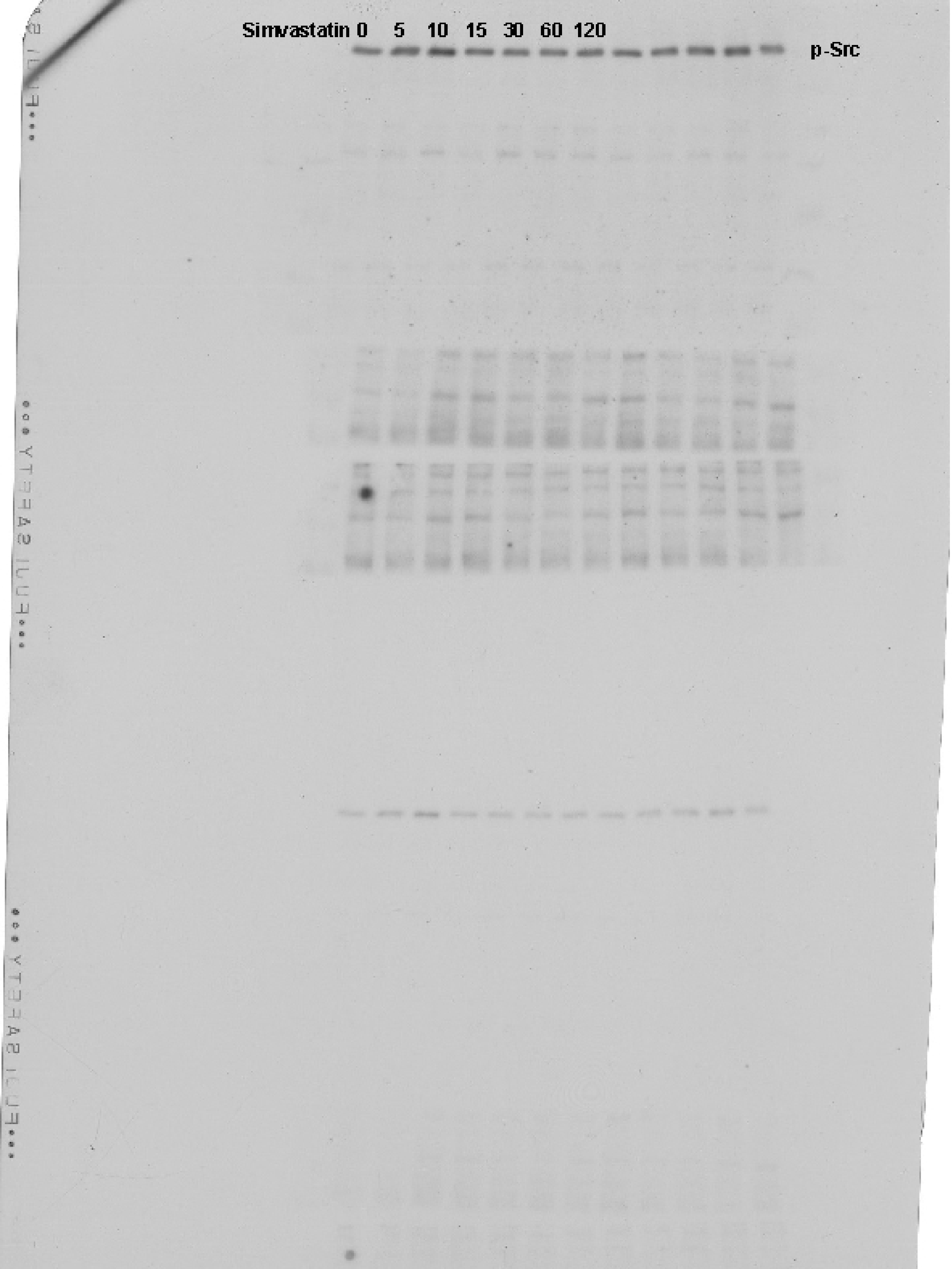
Simvastatin 0 5 10 15 30 60 120
p-Src

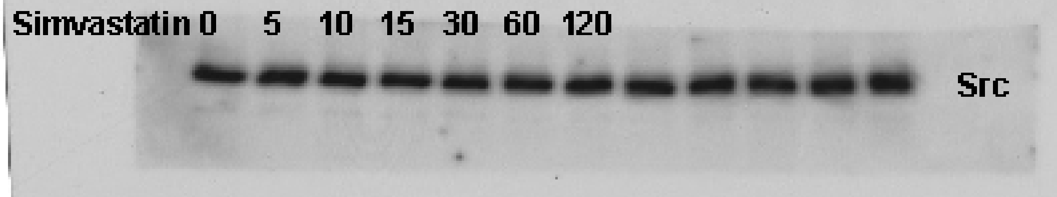
Simvastatin 0 5 10 15 30 60 120
Src

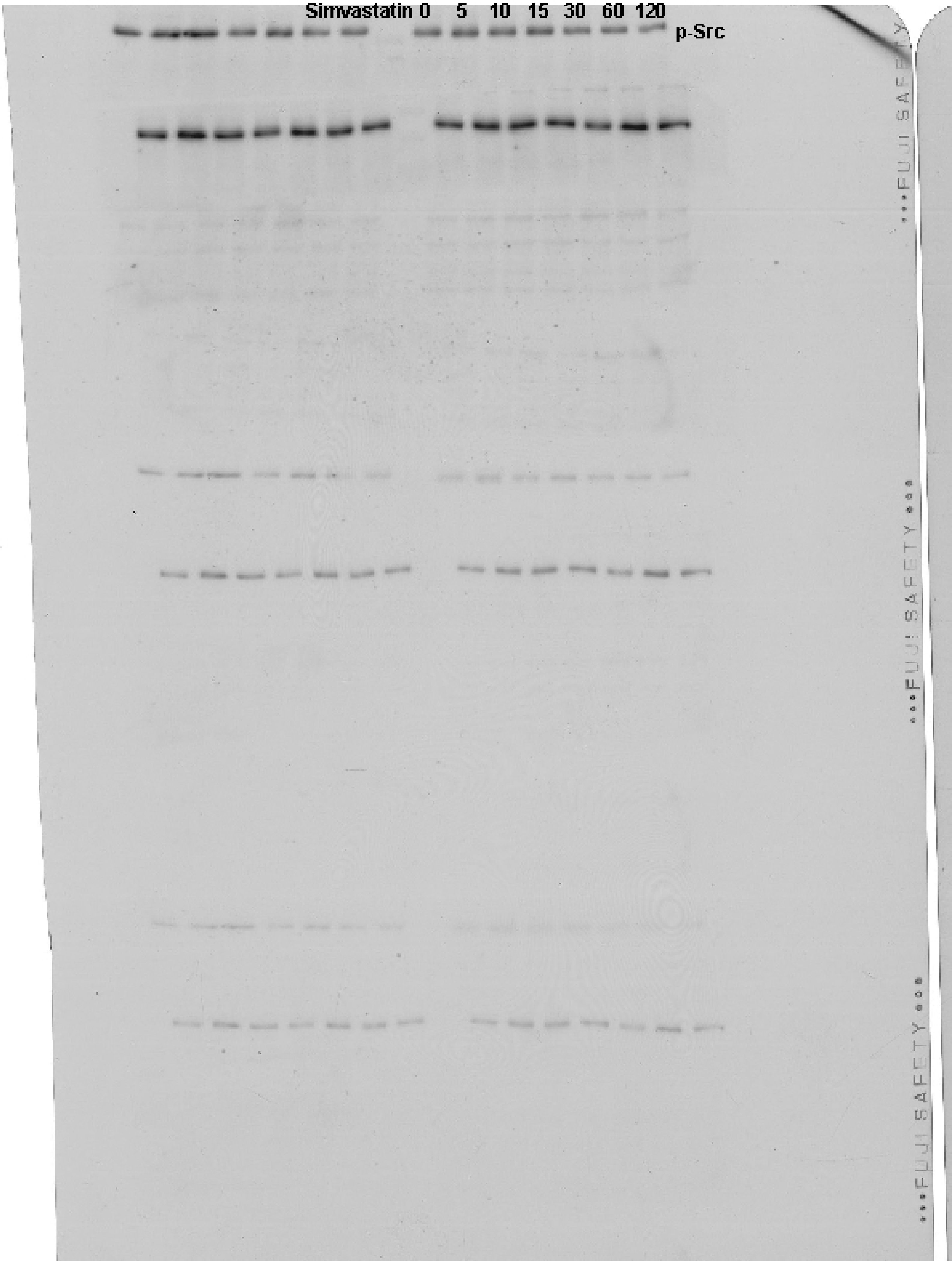
Simvastatin 0 5 10 15 30 60 120
p-Src
FUJI SAFETY

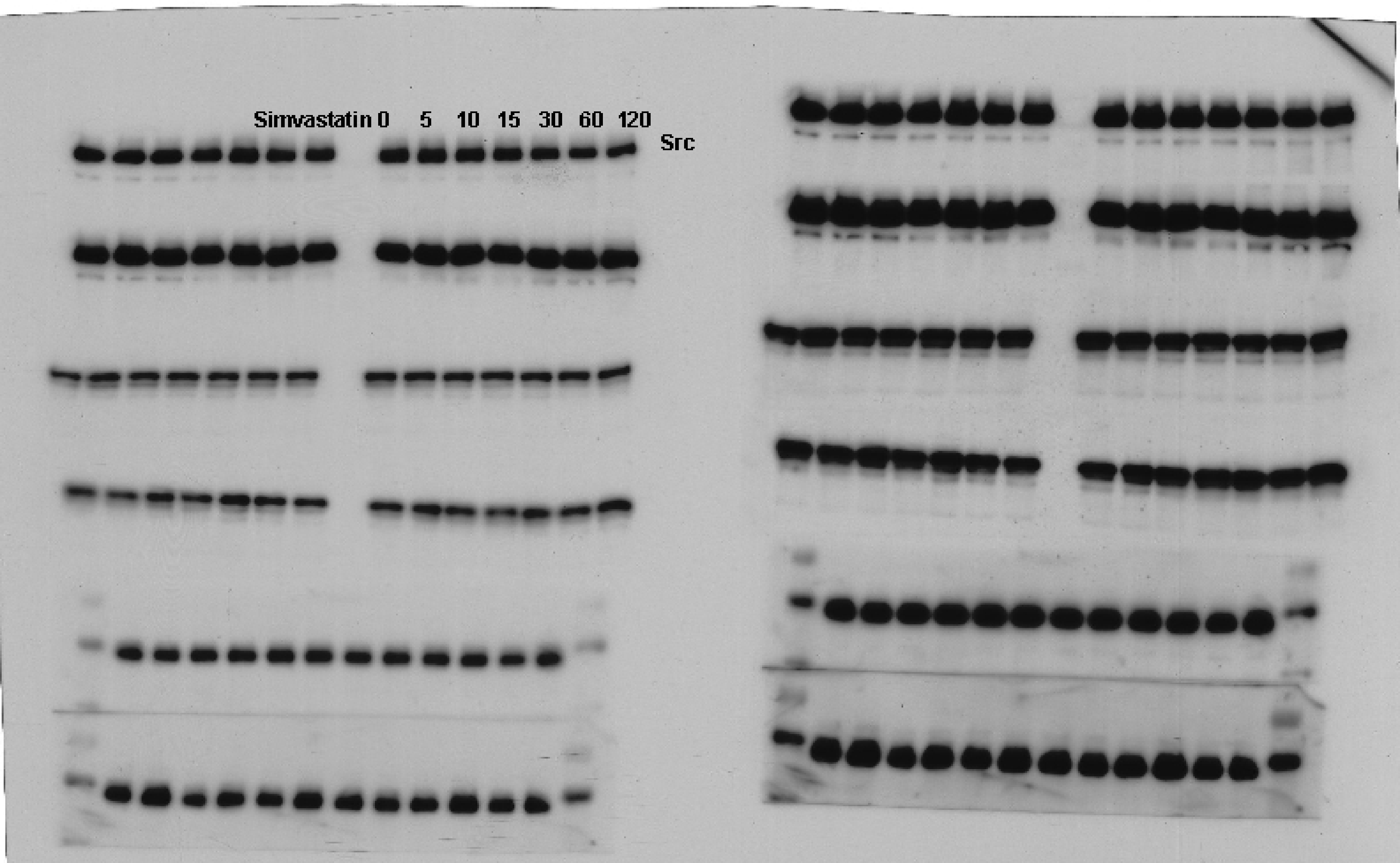
Simvastatin 0 5 10 15 30 60 120
Src

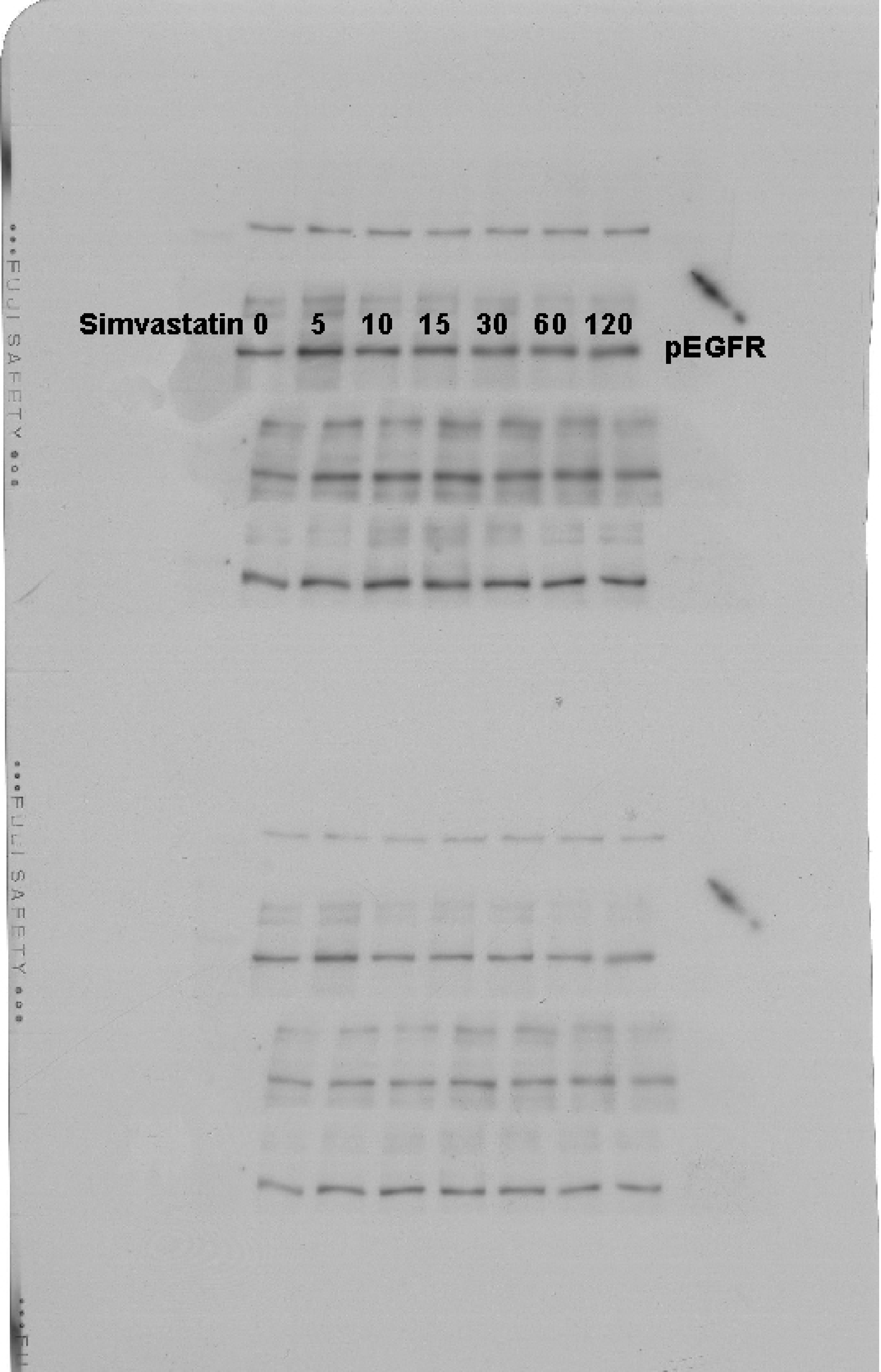
Simvastatin 0 5 10 15 30 60 120
pEGFR

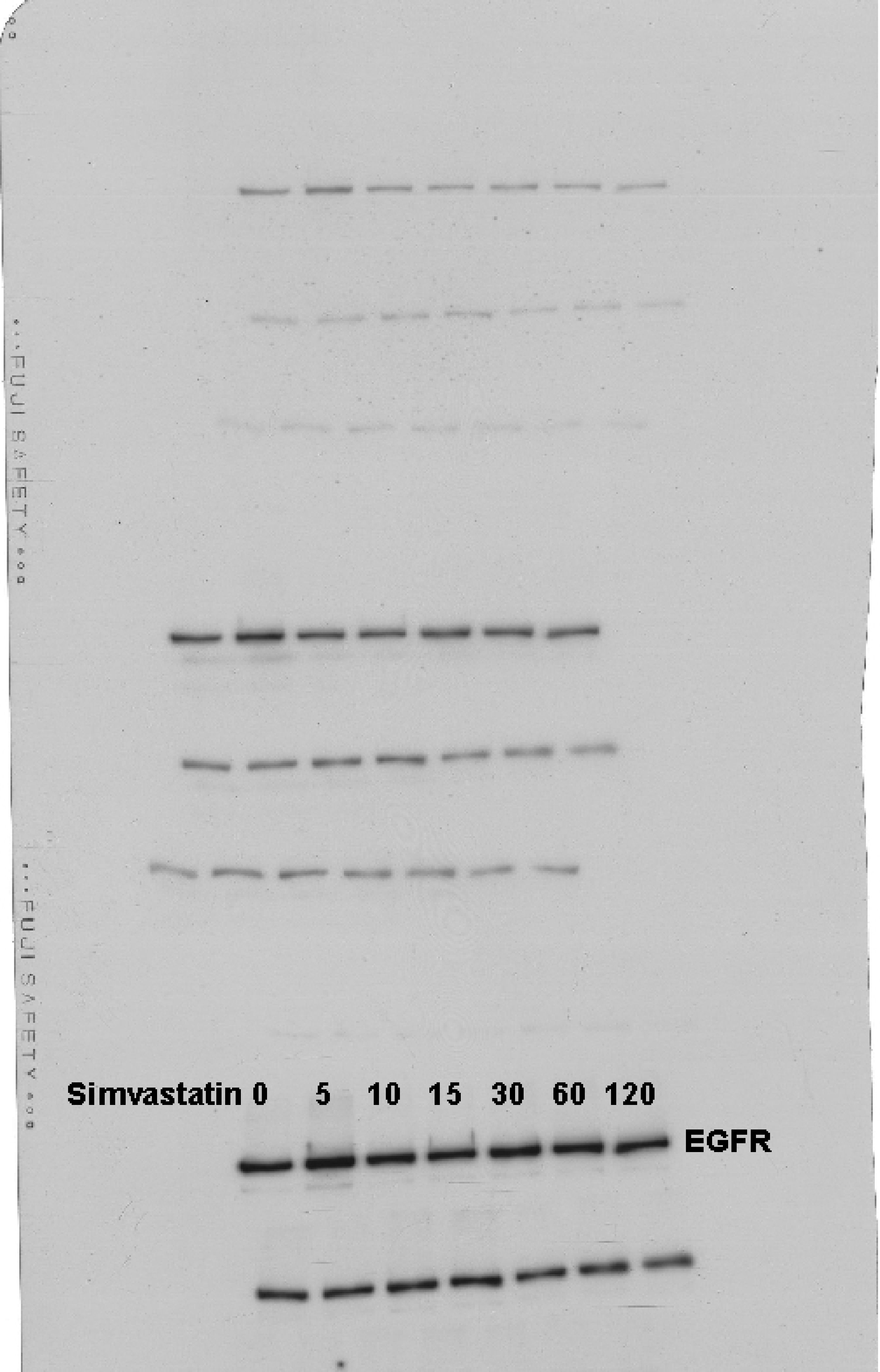
Simvastatin 0 5 10 15 30 60 120
EGFR

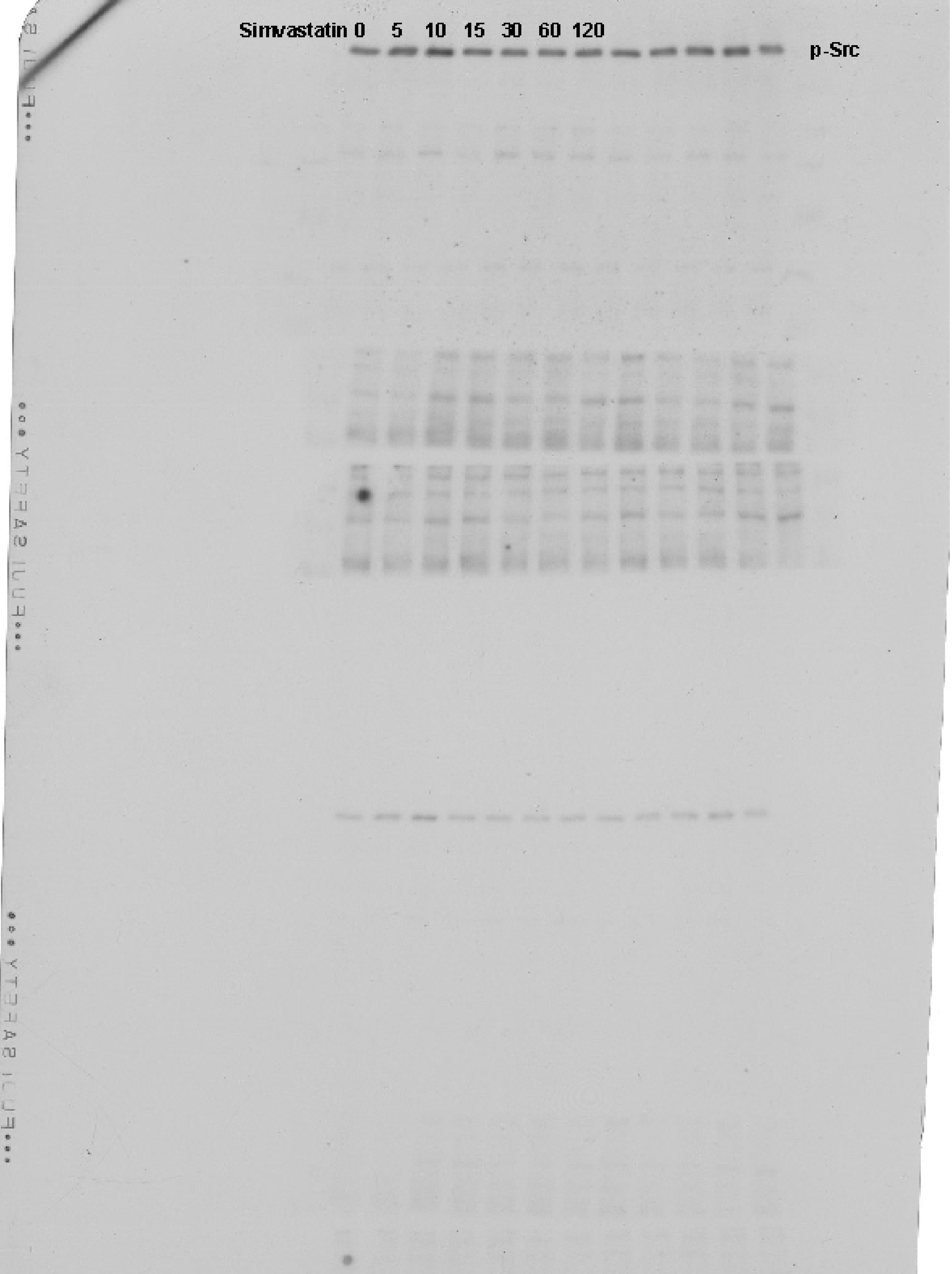
Simvastatin 0 5 10 15 30 60 120
p-Src

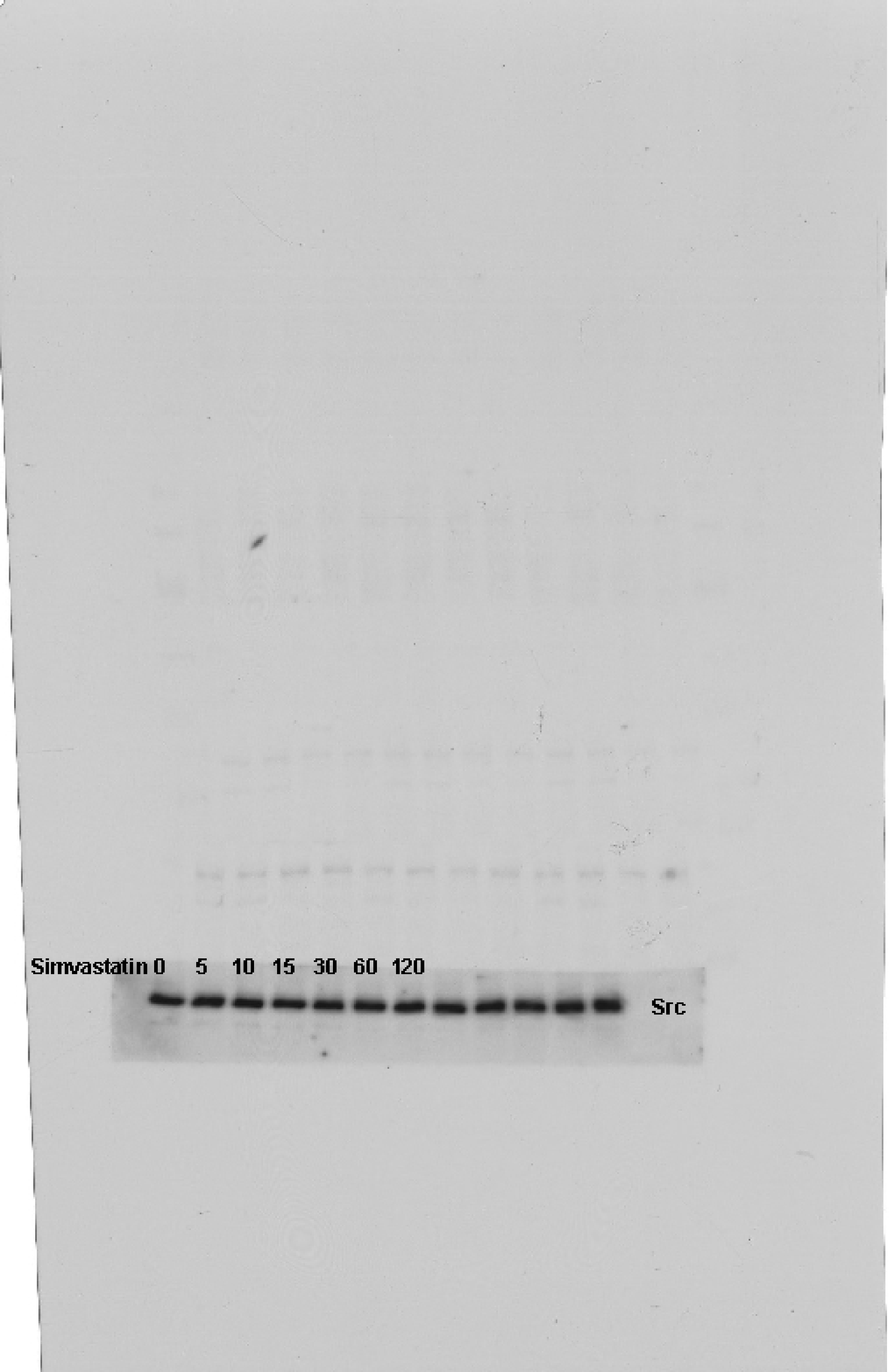

Simvastatin 0 5 10 15 30 60 120
Src

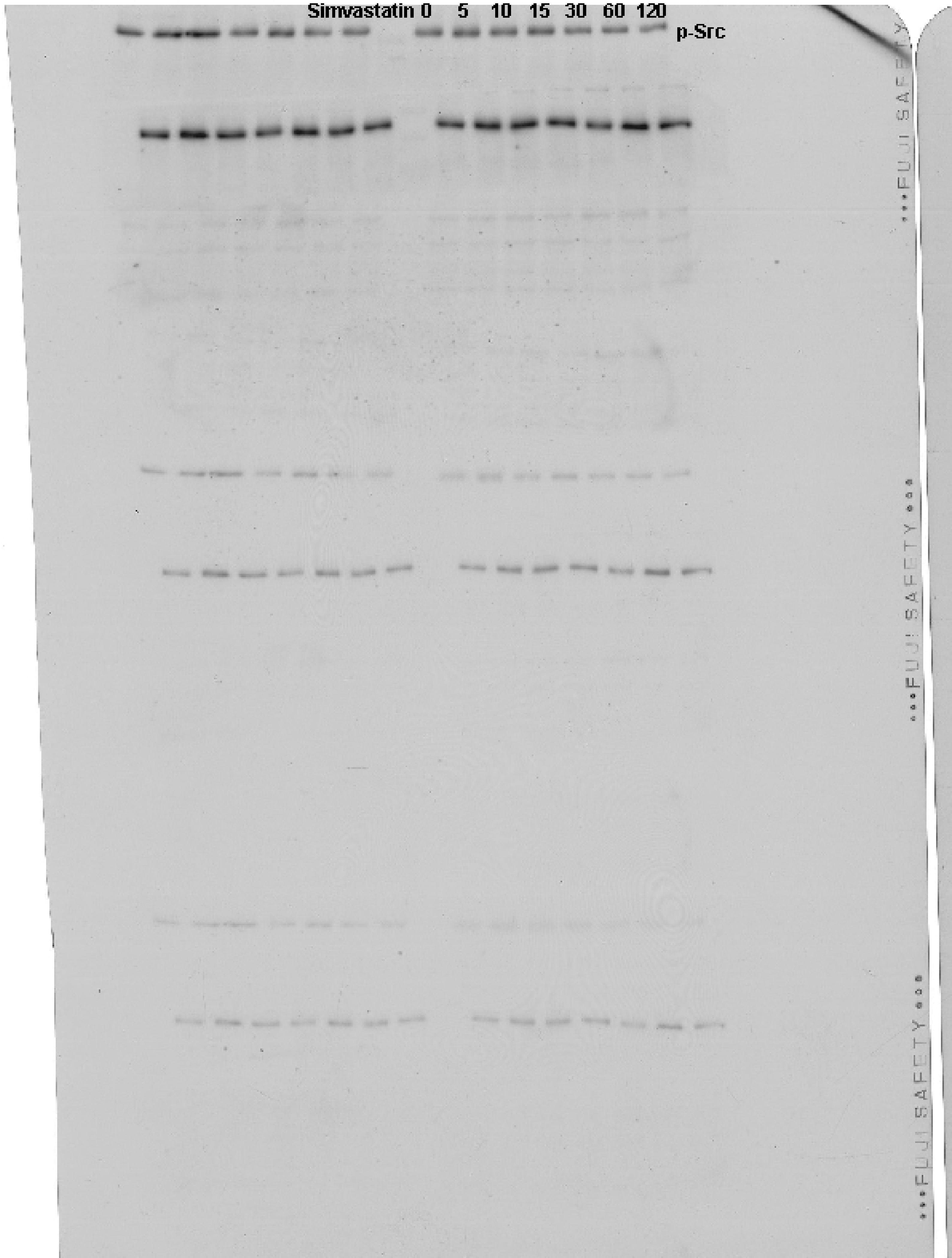
Simvastatin 0 5 10 15 30 60 120
p-Src
FUJI SAFETY

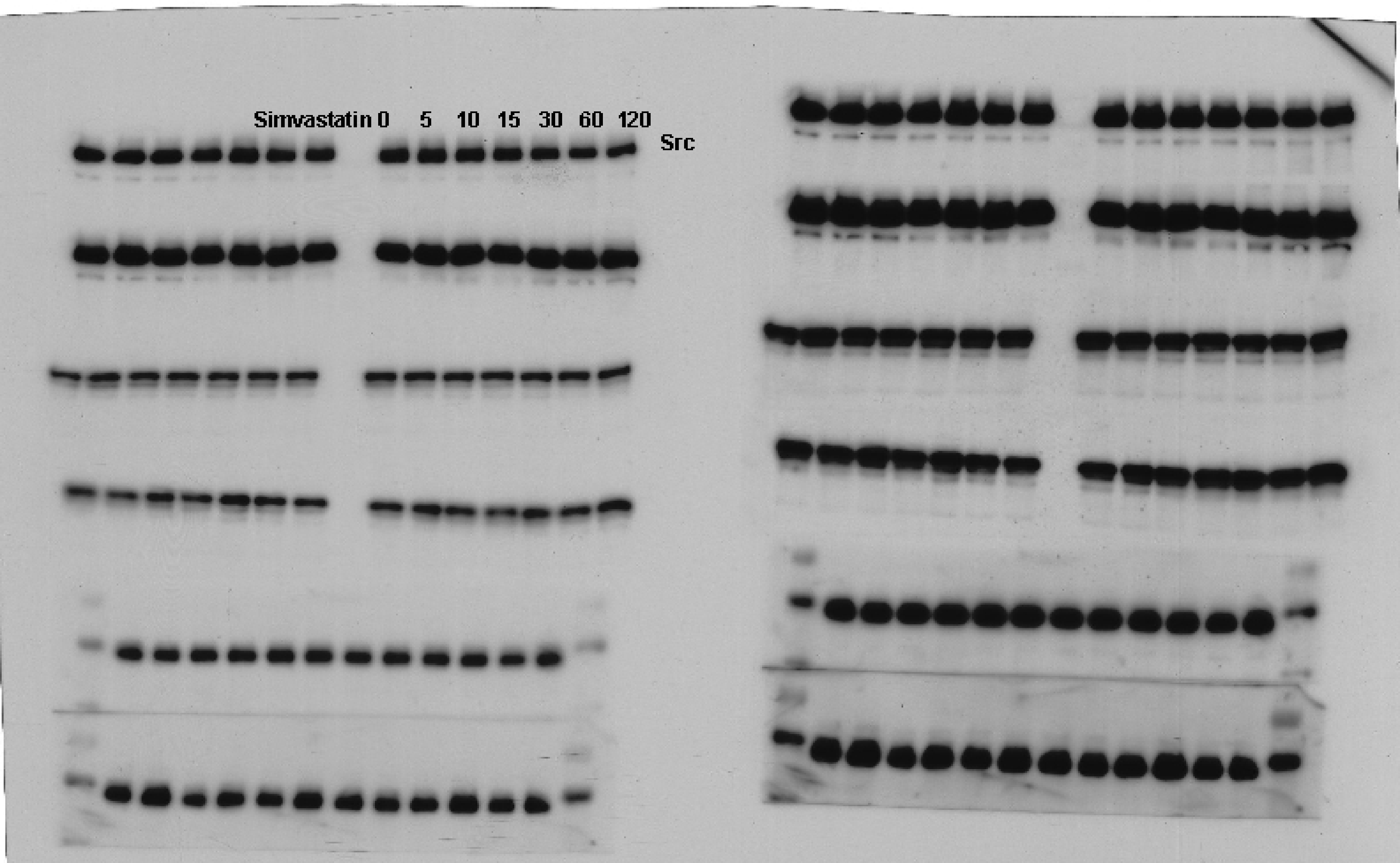
Simvastatin 0 5 10 15 30 60 120
Src

Fig S8. **c-Src signaling is essential for simvastatin-induced sEH tyrosine phosphorylation and the formation of an sEH–Akt–AMPK–eNOS complex.**

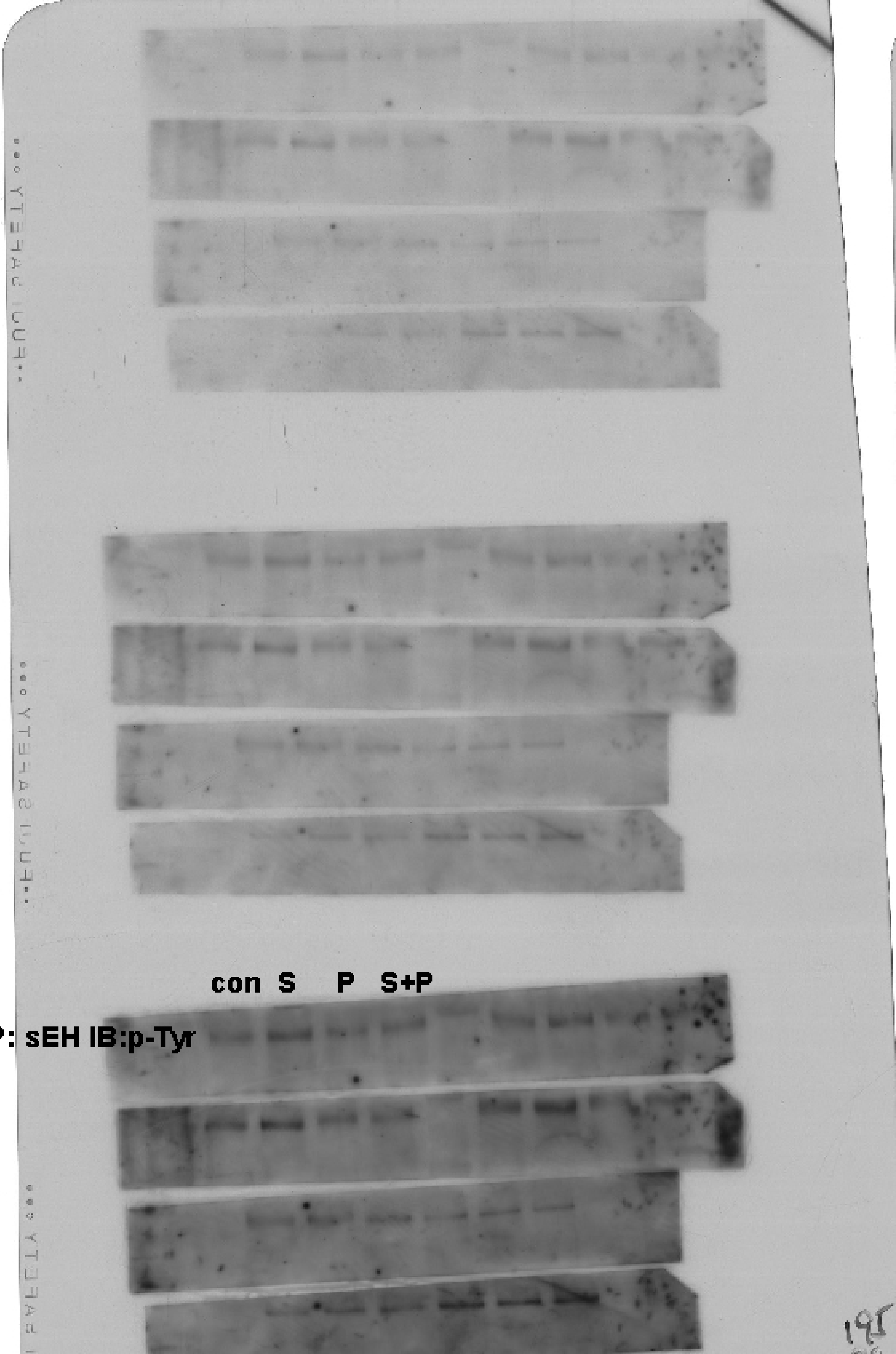
con S P S+P
IP: sEH IB:p-Tyr

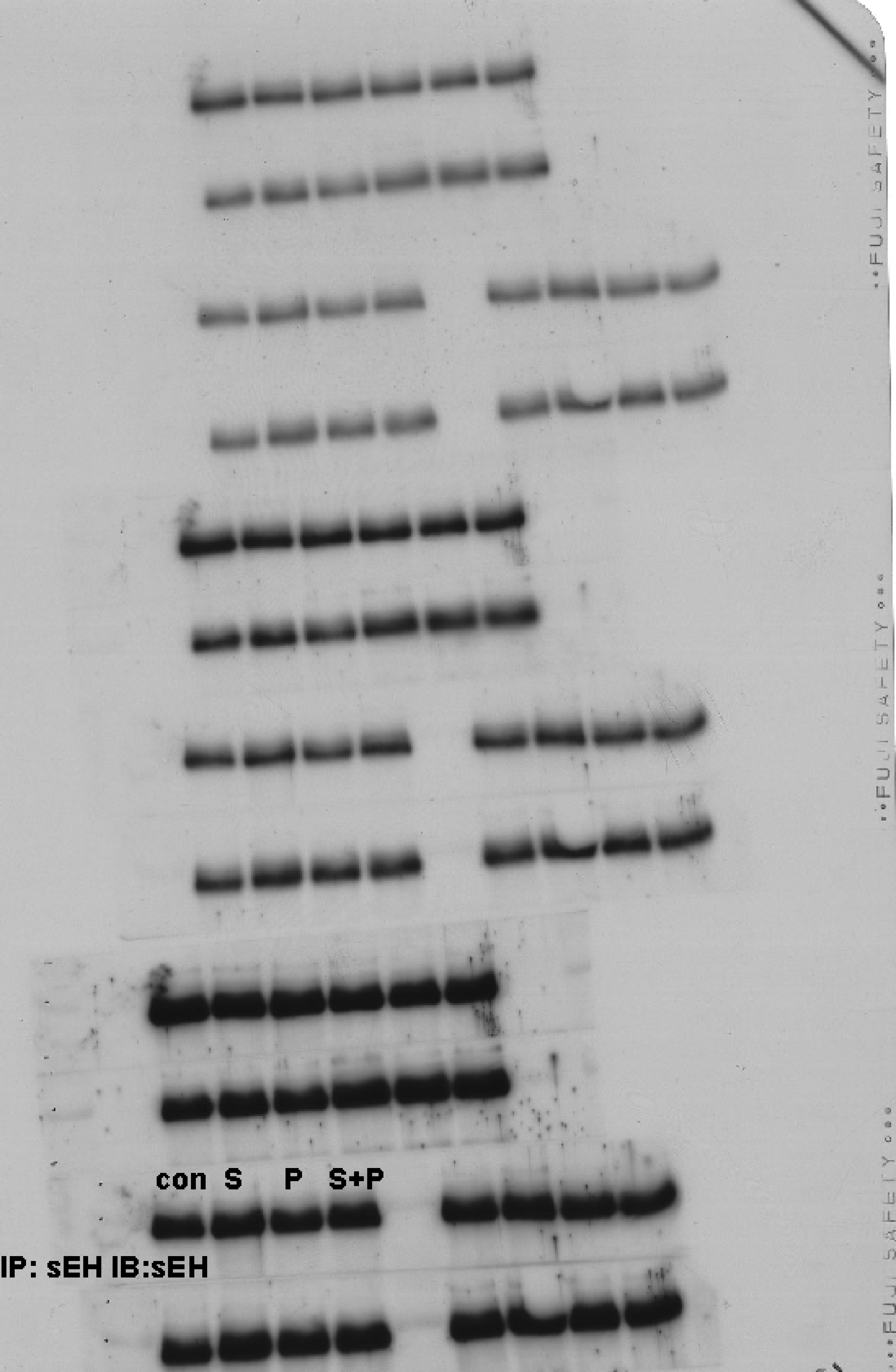
con S P S+P
IP: sEH IB:sEH

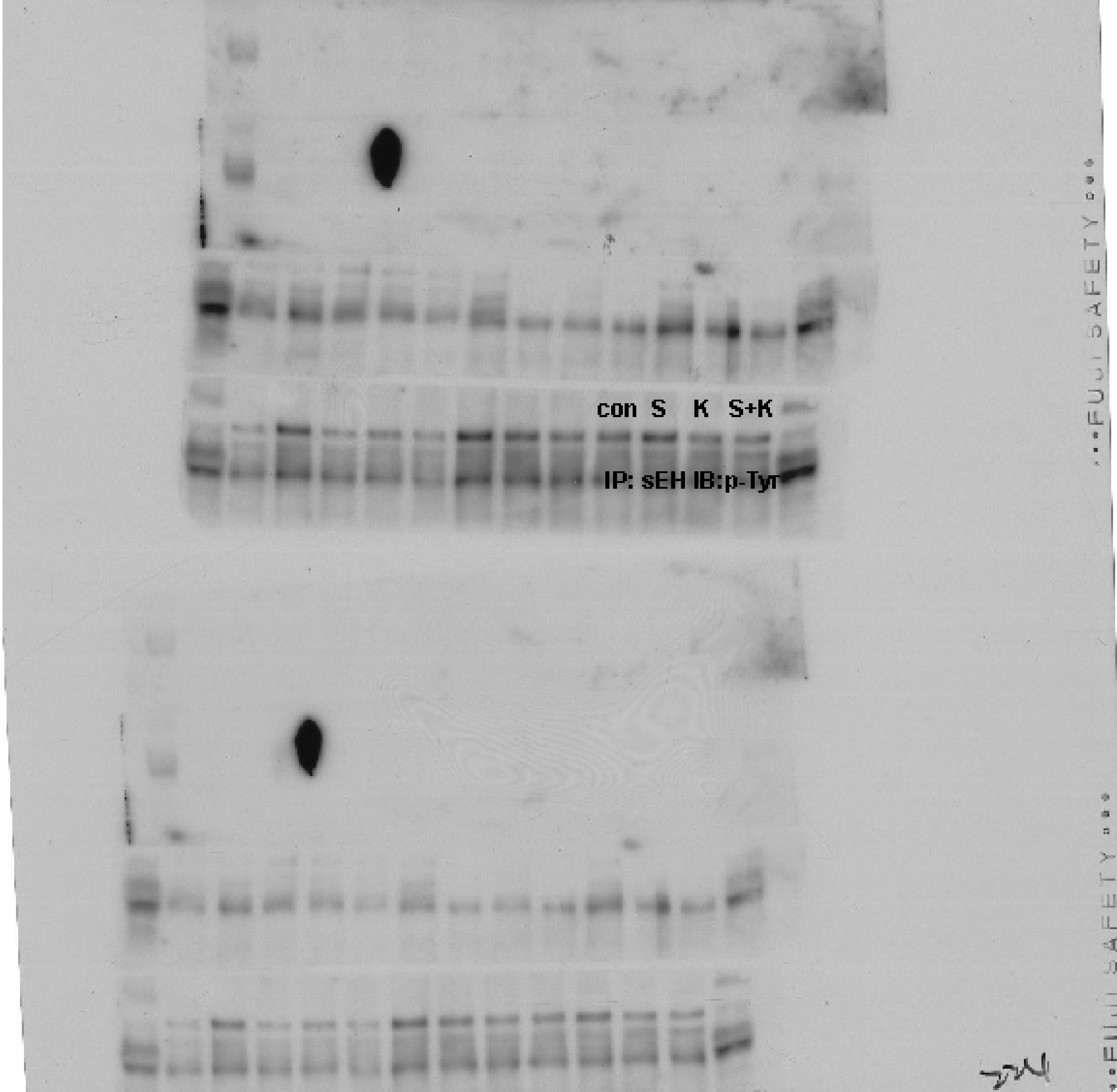
con S K S+K
IP: sEH IB:p-Tyr
FUJI SAFETY
FUJI SAFETY

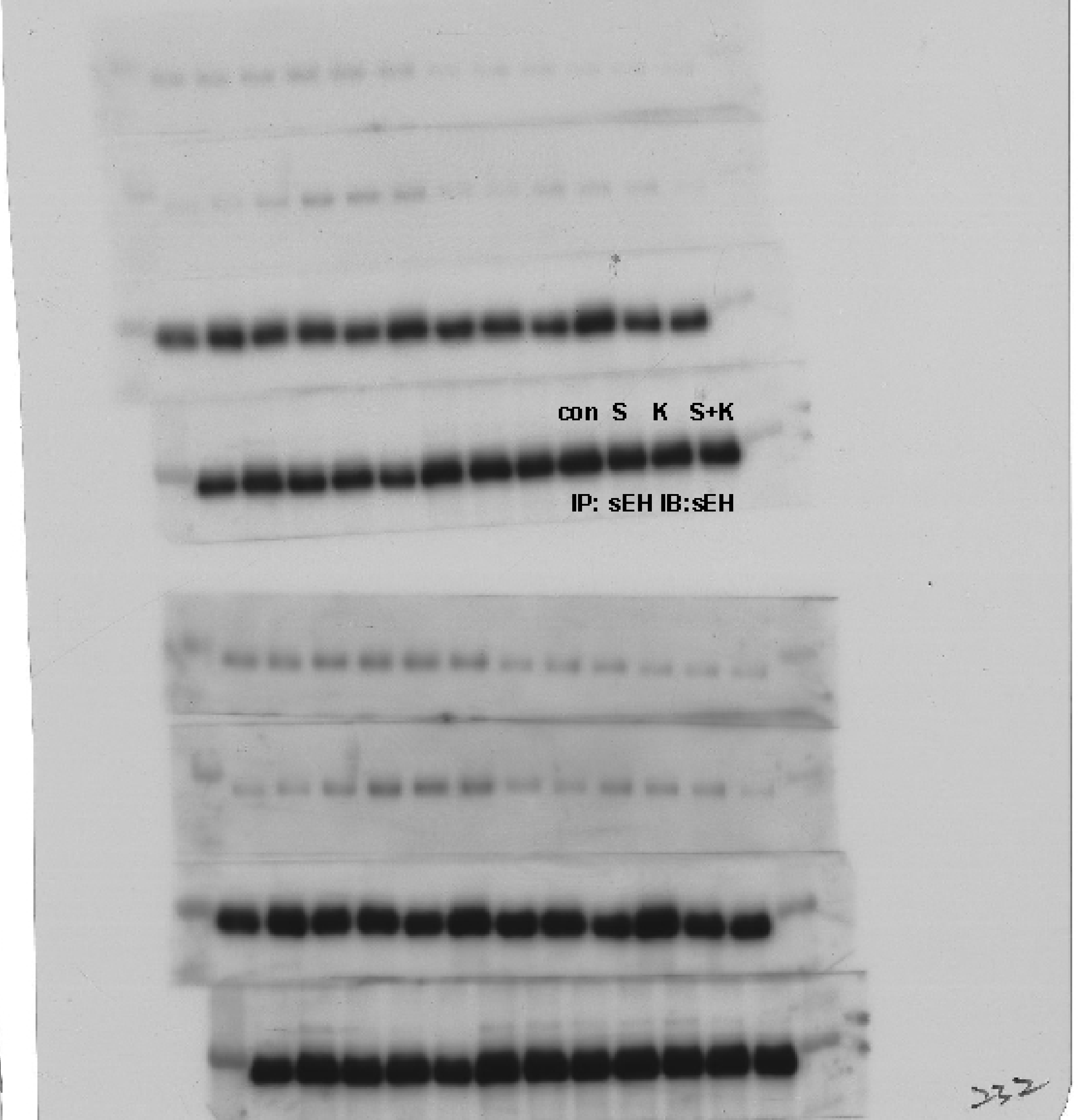

con S K S+K
IP: sEH IB:sEH

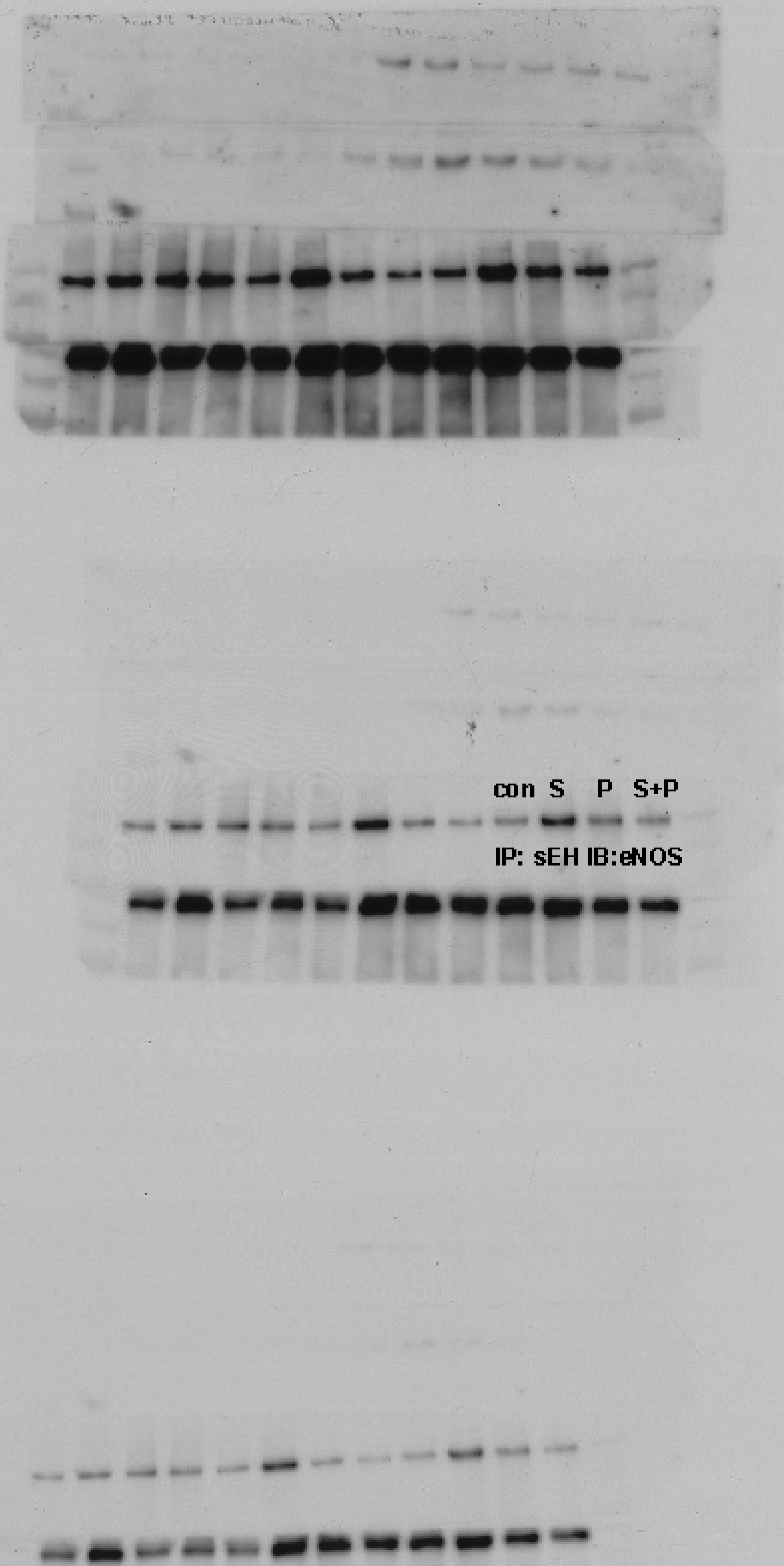
con S P S+P
IP: sEH IB:eNOS

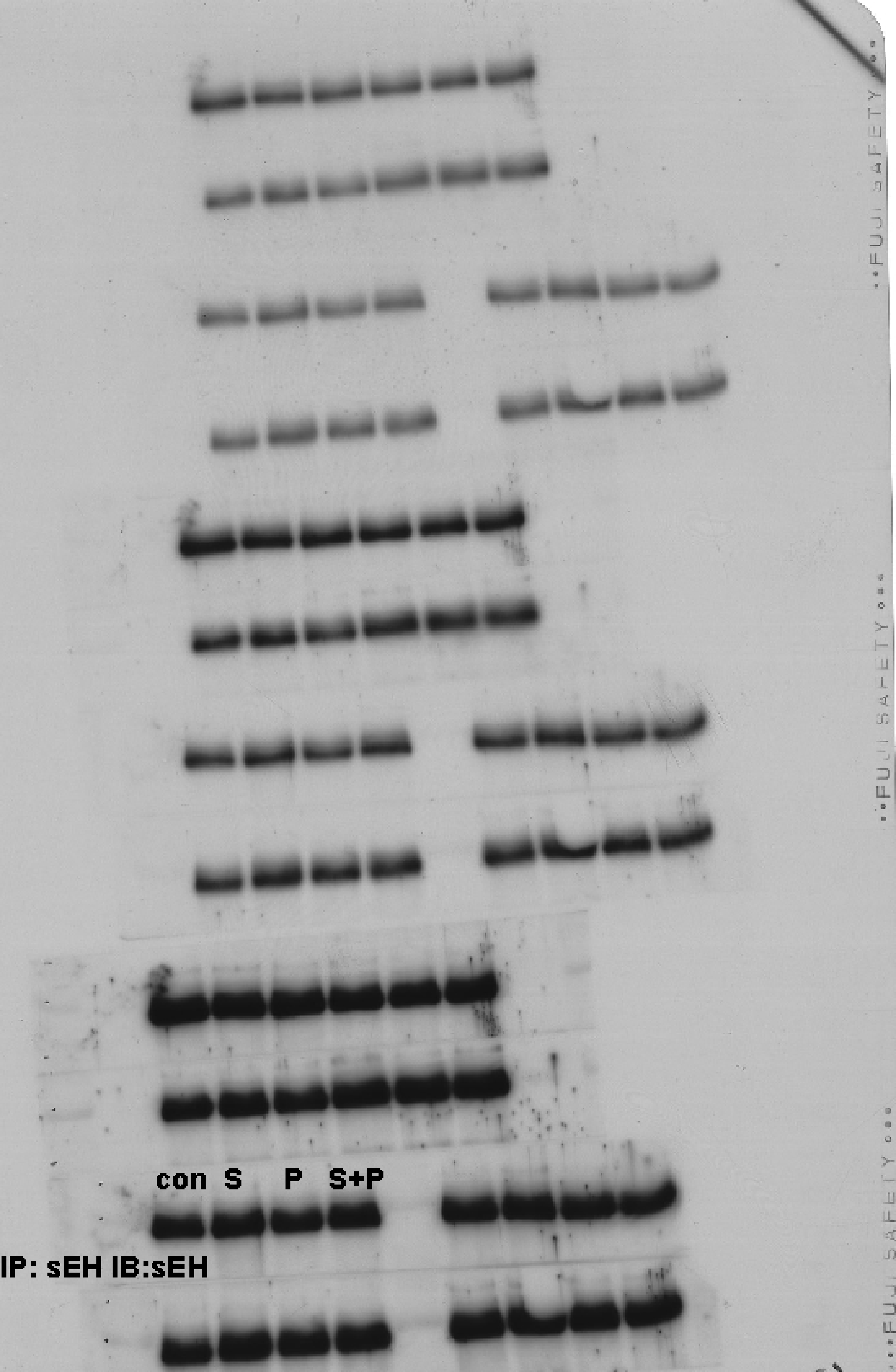
con S P S+P
IP: sEH IB:sEH
FUJI SAFETY

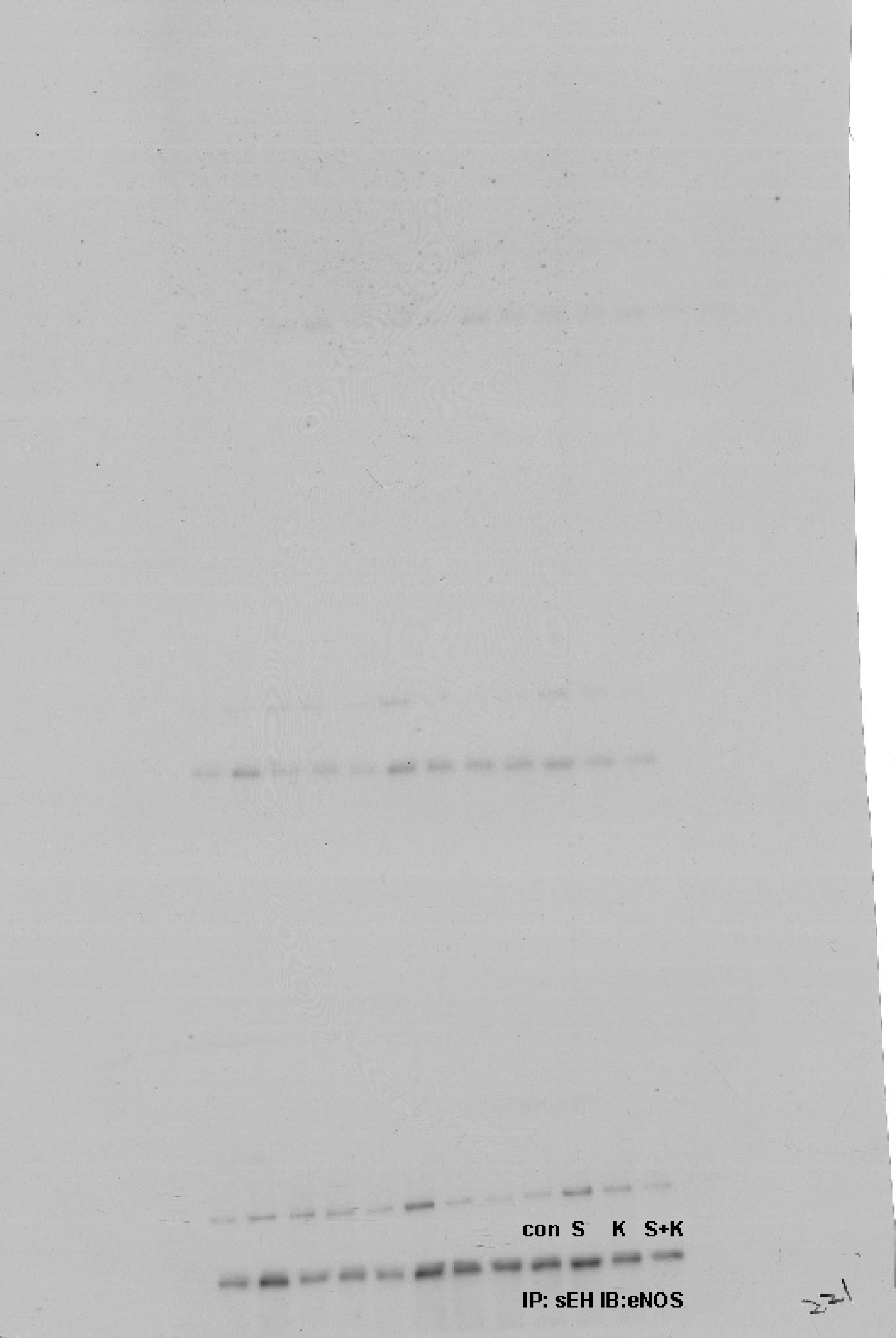
con S K S+K
IP: sEH IB:eNOS

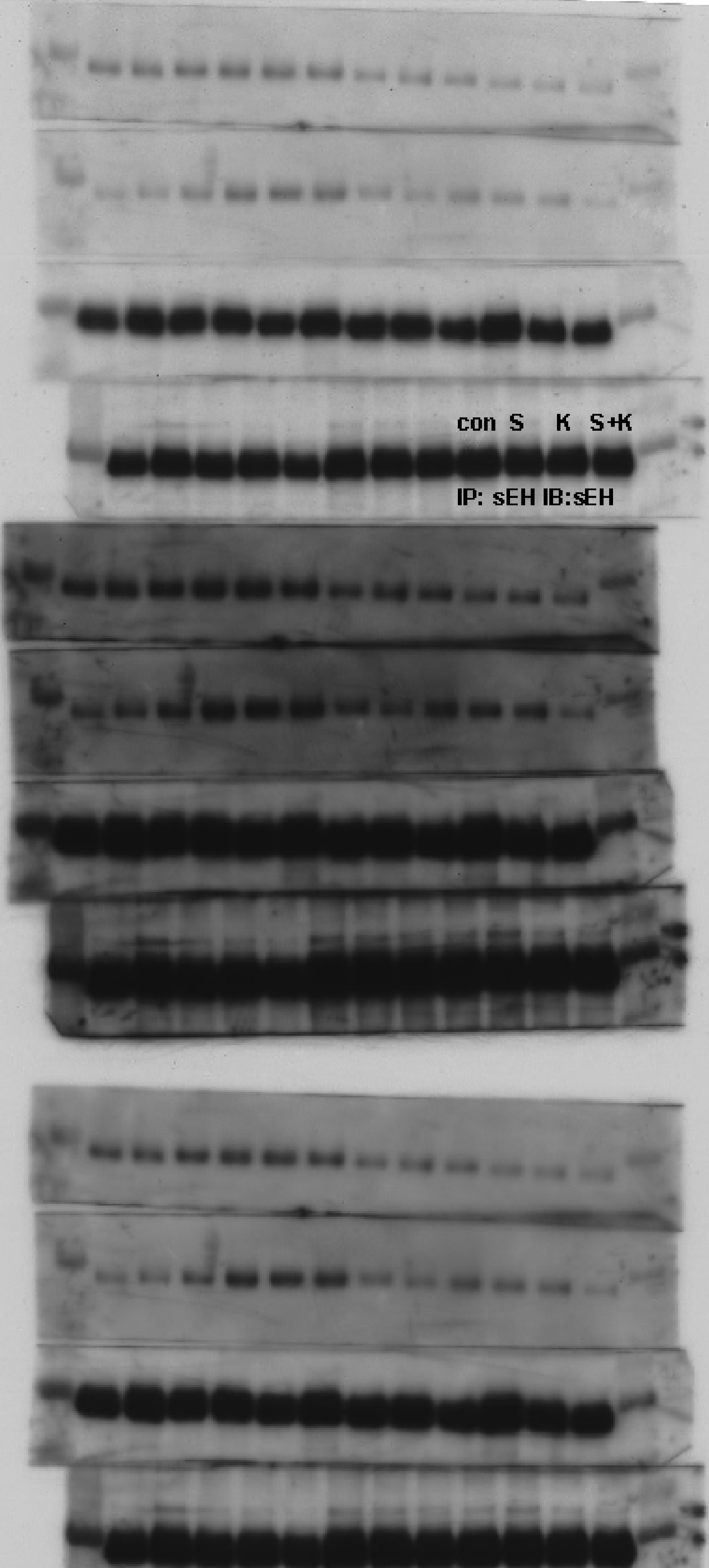
con S K S+K
IP: sEH IB:sEH

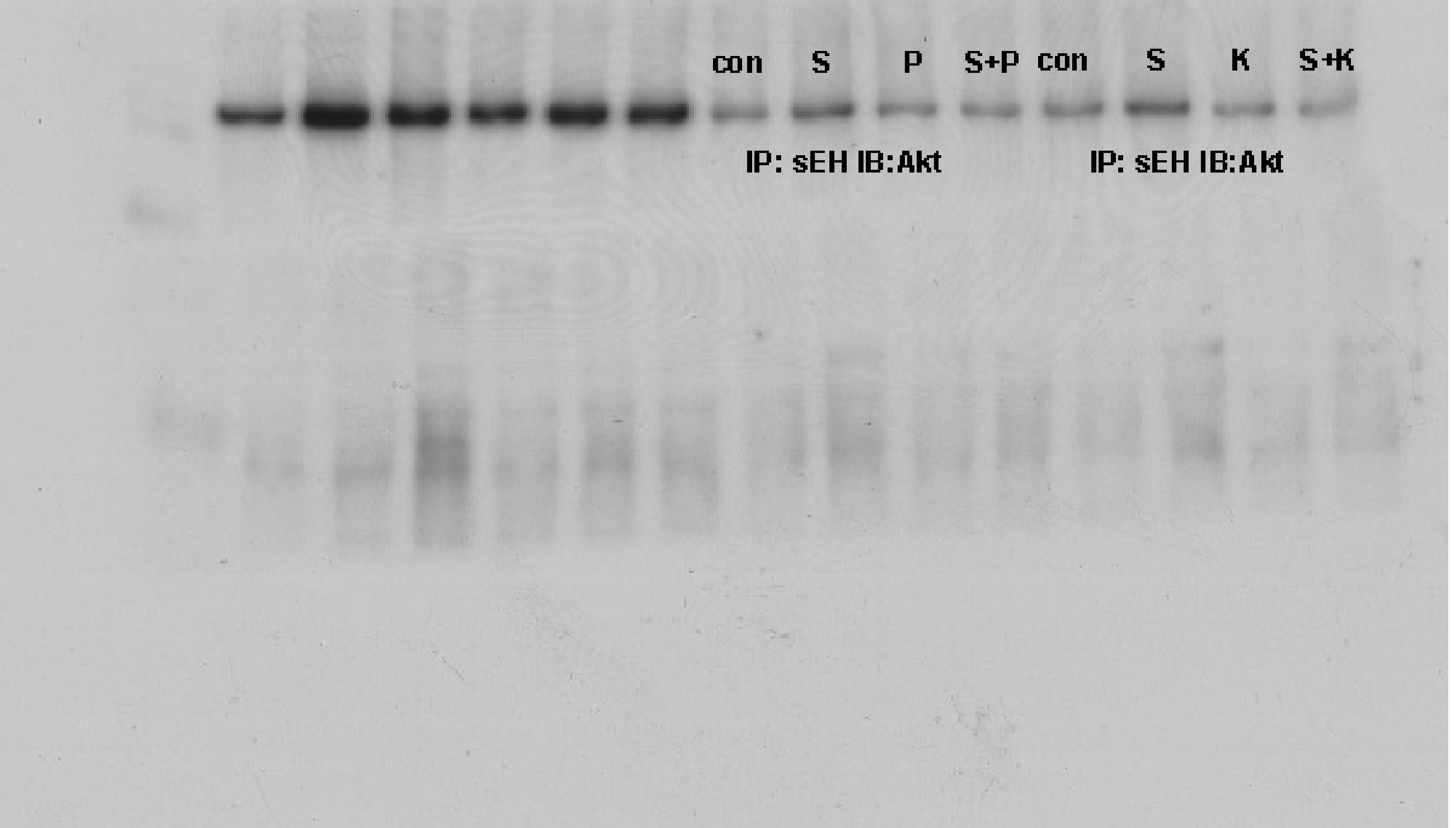
con S P S+P con S K S+K
IP: sEH IB:Akt
IP: sEH IB:Akt

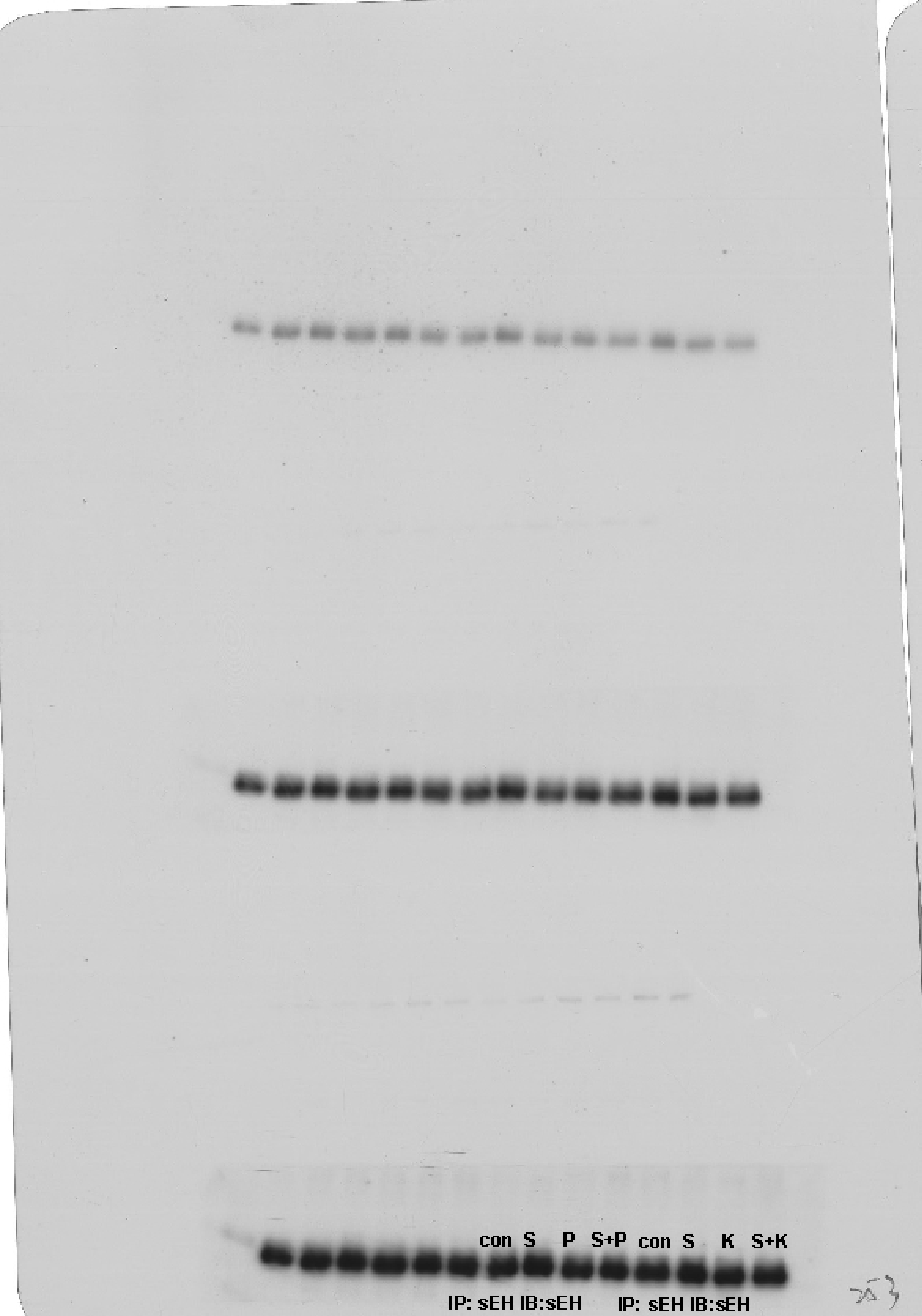
con S P S+P con S K S+K
IP: sEH IB:sEH
IP: sEH IB:sEH

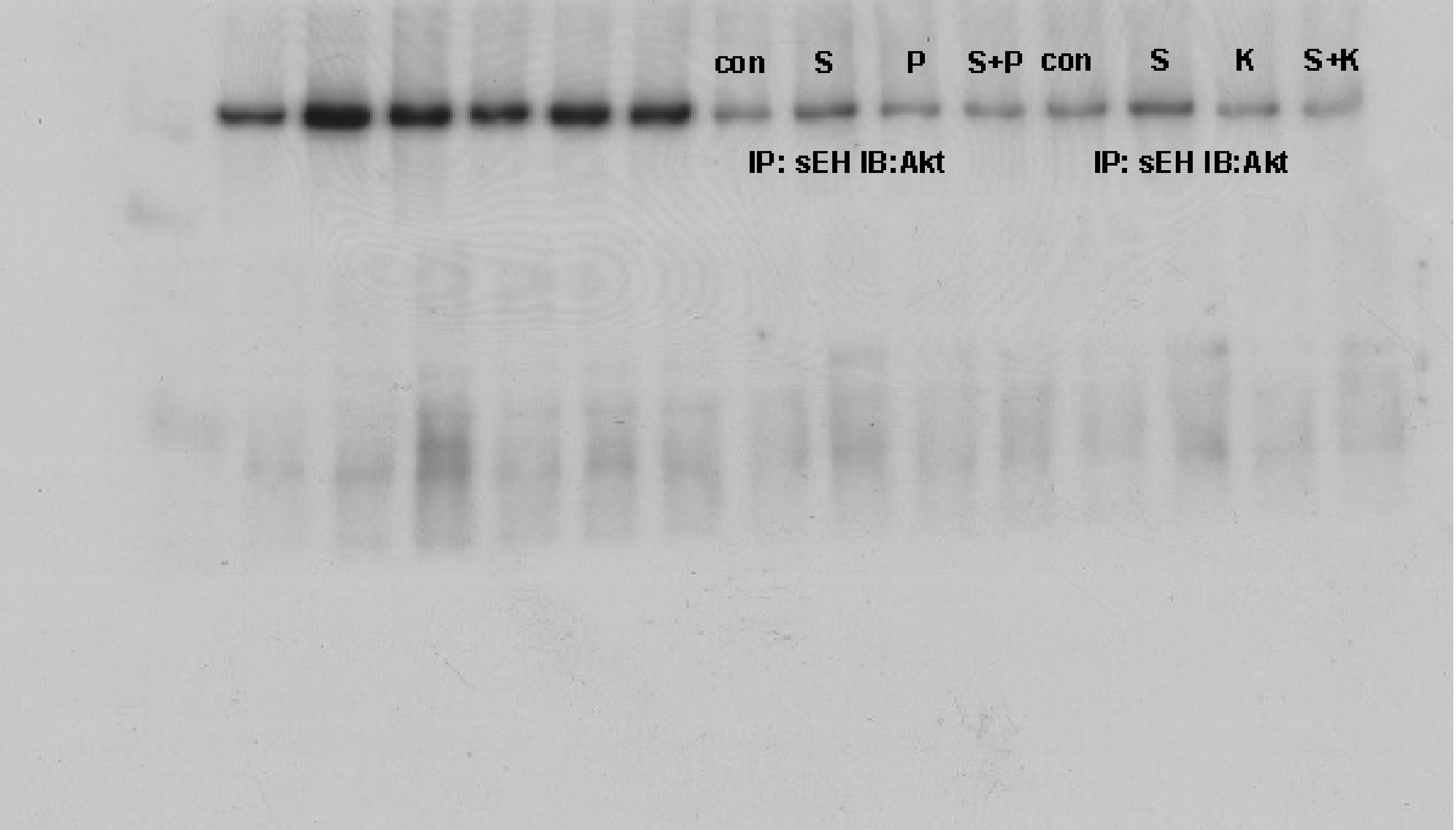
con S P S+P con S K S+K
IP: sEH IB:Akt
IP: sEH IB:Akt

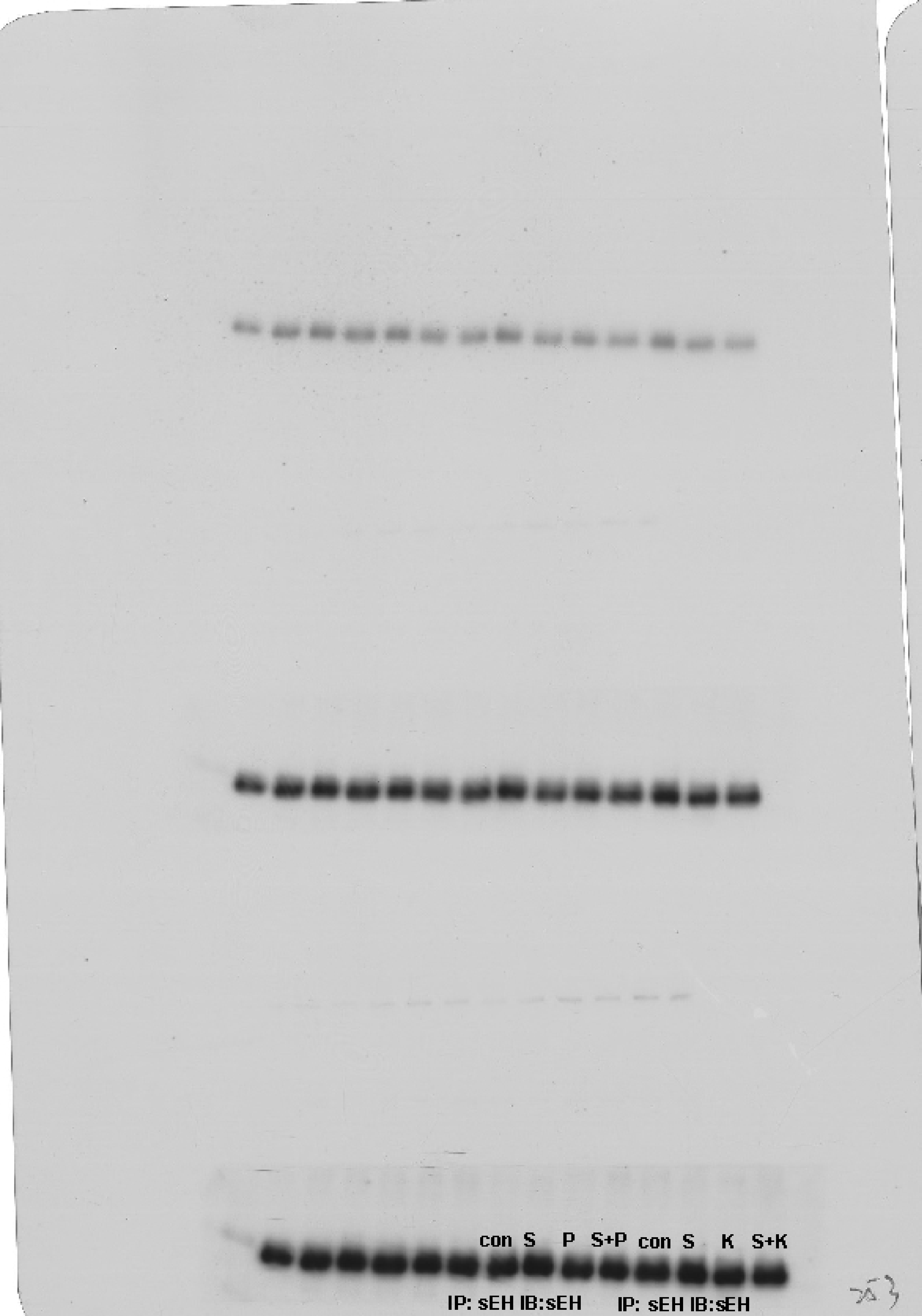
con S P S+P con S K S+K
IP: sEH IB:sEH
IP: sEH IB:sEH

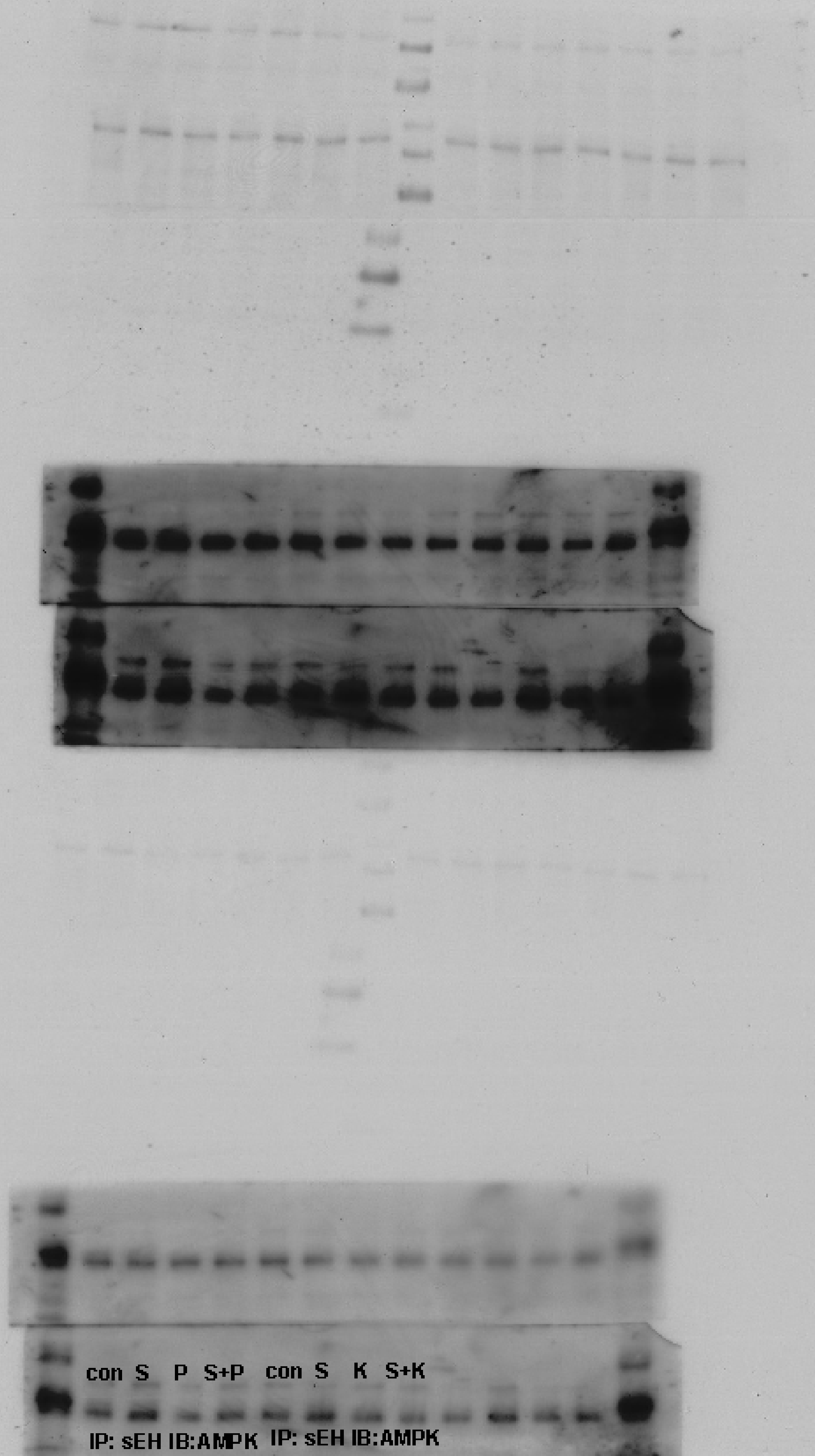
con S P S+P con S K S+K
IP: sEH IB:AMPK IP: sEH IB:AMPK

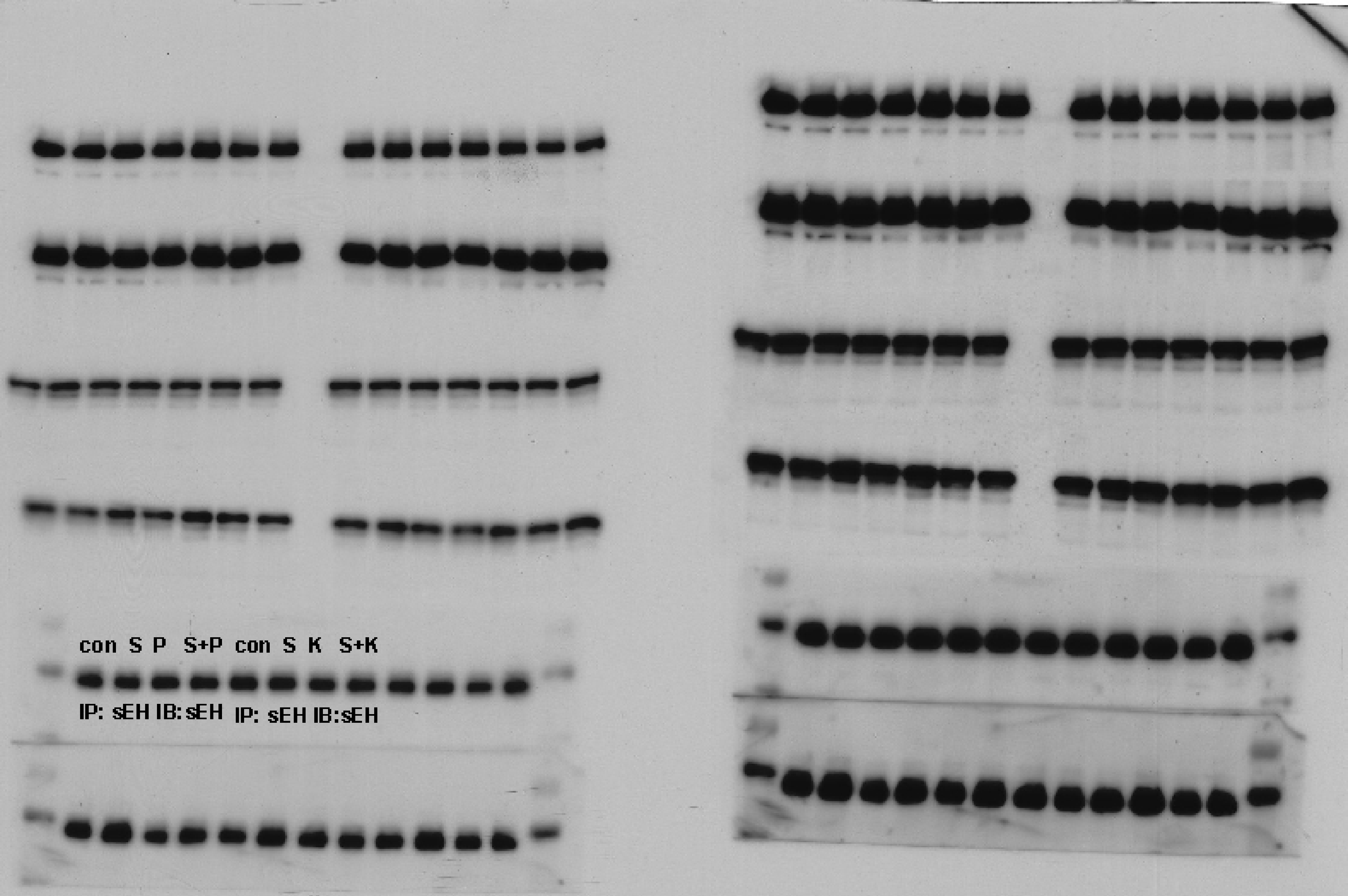
con S P S+P con S K S+K
IP: sEH IB: sEH IP: sEH IB:sEH

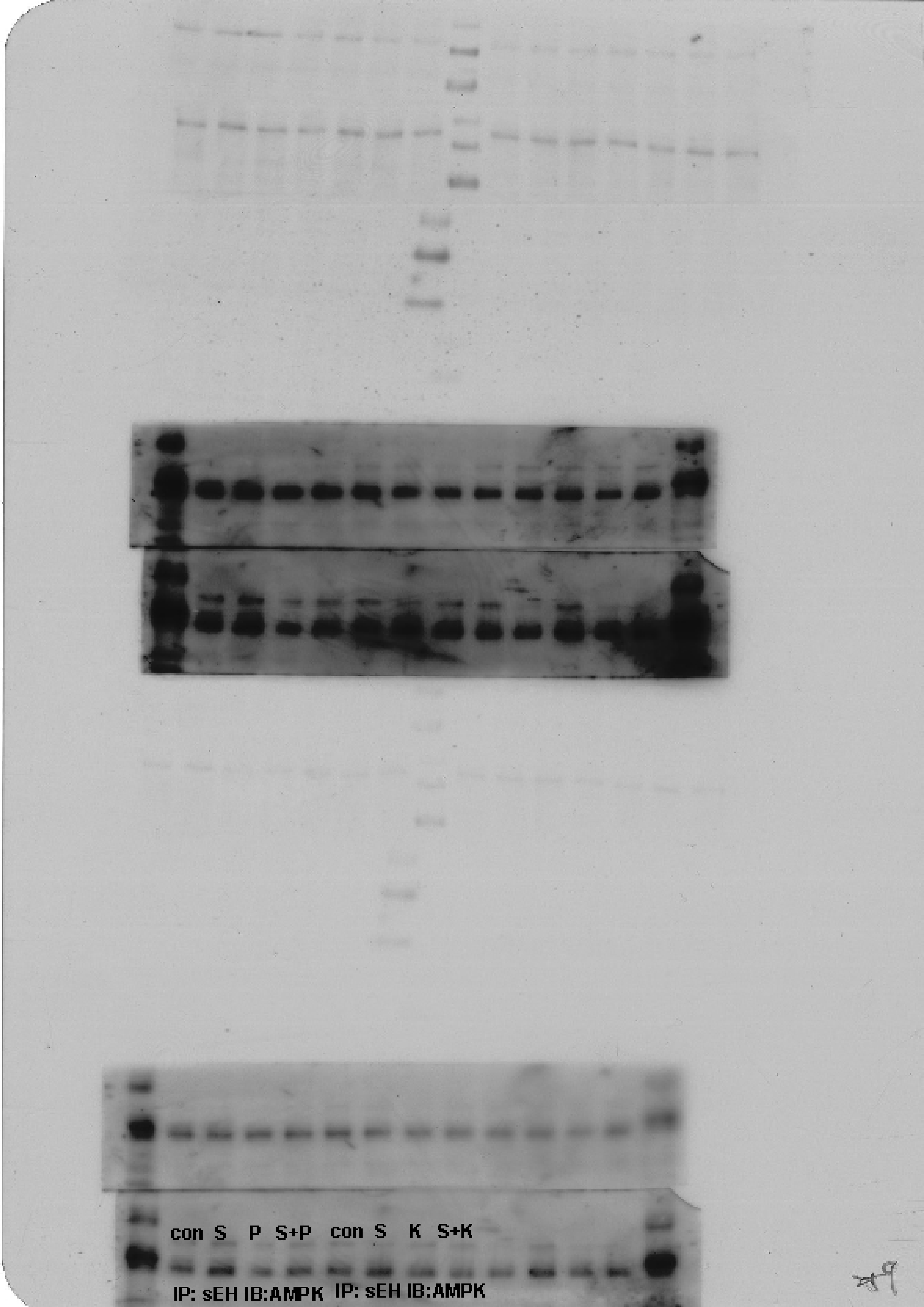
con S P S+P con S K S+K
IP: sEH IB:AMPK IP: sEH IB:AMPK

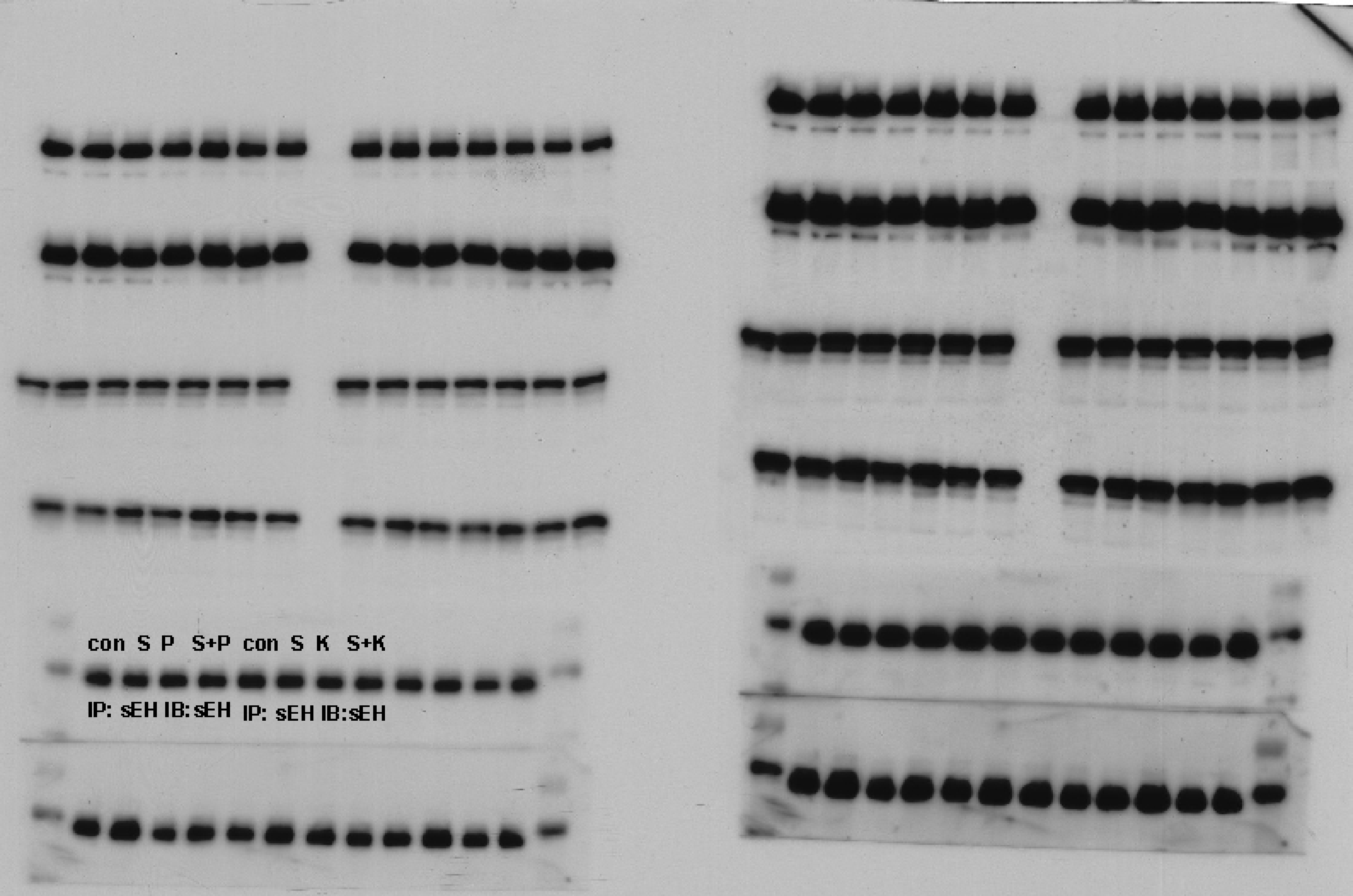
con S P S+P con S K S+K
IP: sEH IB:sEH
IP: sEH IB:sEH
